# Chromosome-level genome assembly and transcriptomes of the leaf insect *Cryptophyllium westwoodii* provide insights into the evolution of leaf-like masquerade

**DOI:** 10.1093/gigascience/giag022

**Published:** 2026-03-02

**Authors:** Chuyang Mao, Zhiwei Dong, Zihe Li, Botong Zhou, Yi Hu, Jun Li, Guichun Liu, Zheng Zhou, Jinwu He, Yuhan Wu, Wenting Wan, Haoran Gao, Ruoping Zhao, Wenhui Nie, Wen Wang, Xueyan Li

**Affiliations:** State Key Laboratory of Genetic Evolution & Animal Models, Kunming Institute of Zoology, Chinese Academy of Sciences, Kunming 650223, China; Kunming College of Life Science, University of Chinese Academy of Sciences, Kunming 650223, China; State Key Laboratory of Genetic Evolution & Animal Models, Kunming Institute of Zoology, Chinese Academy of Sciences, Kunming 650223, China; New Cornerstone Science Laboratory, Shaanxi Key Laboratory of Qinling Ecological Intelligent Monitoring and Protection, Department of Ecology and Environment, School of Life Science and Technology, Northwestern Polytechnical University, Xi’an 710072, China; New Cornerstone Science Laboratory, Shaanxi Key Laboratory of Qinling Ecological Intelligent Monitoring and Protection, Department of Ecology and Environment, School of Life Science and Technology, Northwestern Polytechnical University, Xi’an 710072, China; State Key Laboratory of Genetic Evolution & Animal Models, Kunming Institute of Zoology, Chinese Academy of Sciences, Kunming 650223, China; State Key Laboratory of Genetic Evolution & Animal Models, Kunming Institute of Zoology, Chinese Academy of Sciences, Kunming 650223, China; Kunming College of Life Science, University of Chinese Academy of Sciences, Kunming 650223, China; State Key Laboratory of Genetic Evolution & Animal Models, Kunming Institute of Zoology, Chinese Academy of Sciences, Kunming 650223, China; Department of Basic Medicine, College of Medicine, Xi’an International University, Xi’an 710077, China; State Key Laboratory of Genetic Evolution & Animal Models, Kunming Institute of Zoology, Chinese Academy of Sciences, Kunming 650223, China; New Cornerstone Science Laboratory, Shaanxi Key Laboratory of Qinling Ecological Intelligent Monitoring and Protection, Department of Ecology and Environment, School of Life Science and Technology, Northwestern Polytechnical University, Xi’an 710072, China; State Key Laboratory of Genetic Evolution & Animal Models, Kunming Institute of Zoology, Chinese Academy of Sciences, Kunming 650223, China; State Key Laboratory of Genetic Evolution & Animal Models, Kunming Institute of Zoology, Chinese Academy of Sciences, Kunming 650223, China; Kunming College of Life Science, University of Chinese Academy of Sciences, Kunming 650223, China; State Key Laboratory of Genetic Evolution & Animal Models, Kunming Institute of Zoology, Chinese Academy of Sciences, Kunming 650223, China; Department of Entomology, College of Plant Protection, Yunnan Agricultural University, Kunming 650223, China; State Key Laboratory of Genetic Evolution & Animal Models, Kunming Institute of Zoology, Chinese Academy of Sciences, Kunming 650223, China; State Key Laboratory of Genetic Evolution & Animal Models, Kunming Institute of Zoology, Chinese Academy of Sciences, Kunming 650223, China; State Key Laboratory of Genetic Evolution & Animal Models, Kunming Institute of Zoology, Chinese Academy of Sciences, Kunming 650223, China; New Cornerstone Science Laboratory, Shaanxi Key Laboratory of Qinling Ecological Intelligent Monitoring and Protection, Department of Ecology and Environment, School of Life Science and Technology, Northwestern Polytechnical University, Xi’an 710072, China; Kunming College of Life Science, University of Chinese Academy of Sciences, Kunming 650223, China; State Key Laboratory of Genetic Evolution & Animal Models, Kunming Institute of Zoology, Chinese Academy of Sciences, Kunming 650223, China; Kunming College of Life Science, University of Chinese Academy of Sciences, Kunming 650223, China

**Keywords:** Phylliidae, *Cryptophyllium westwoodii*, high-quality genome, comparative genomics, *Cuticle* genes, leaf-resembling morphology

## Abstract

**Background:**

Leaf insects in the family Phylliidae are regarded as nature’s ultimate masqueraders, evolving the leaf-resembling morphology to avoid predation. However, the lack of a high-quality reference genome for the leaf insects has hindered the exploration of the genetic mechanisms of leaf-like masquerade in insects.

**Results:**

We generated a chromosome-level genome assembly of *Cryptophyllium westwoodii* using Nanopore and Hi-C sequencing. 98.3% of the 4.12 Gb assembly (scaffold N50 = 256.9 Mb, 98.6% BUSCO completeness) was anchored onto 15 pseudo-chromosomes, including 13 autosomes, an X chromosome, and a putative B chromosome. Genome annotation predicted a total of 2.29 Gb repetitive sequences and 19,131 protein-coding genes. The chromosomal collinearity analysis indicated that many homologous gene fragments were detected between B chromosome and the other 14 A chromosomes in *C. westwoodii*, suggesting the B chromosome could have a mosaic origin based on homologous gene fragments from A chromosomes. Comparative genomic and transcriptomic analyses indicated that *resilin* gene with 24 copies expanded in *C. westwoodii*, of which 10 copies showed significantly different expression in the laterally leaf-like abdominal expansions at five developmental stages. These findings suggest that *Cuticle* genes, particularly *resilin*, may contribute to the leaf masquerade morphology in *C. westwoodii*, implying their possible role in the evolution of this adaptive trait.

**Conclusions:**

This study not only provides the first chromosome-level reference genome of leaf insects in Phylliidae, but also offers new insights into the leaf-like masquerade in leaf insects.

## Introduction

Insecta, as the largest class in the animal kingdom, often employ strategies like crypsis, masquerade, and mimicry through morphological changes across species or developmental stages to evade predators or enhance hunting efficiency due to their position at the food chain’s base [1–3]. Masquerade refers to the accurate imitation of the surrounding bark, leaves, or flowers, which widely exists in various insect groups [4]. For examples, the body and limbs of the orchid mantis (*Hymenopus coronatus*) have evolved the structures and colors similar to orchid petals [5, 6], and the wings of the dead-leaf butterfly (*Kallima inachus*) show the shape, veins, and color of withered leaves [7, 8].

**Figure 1:**
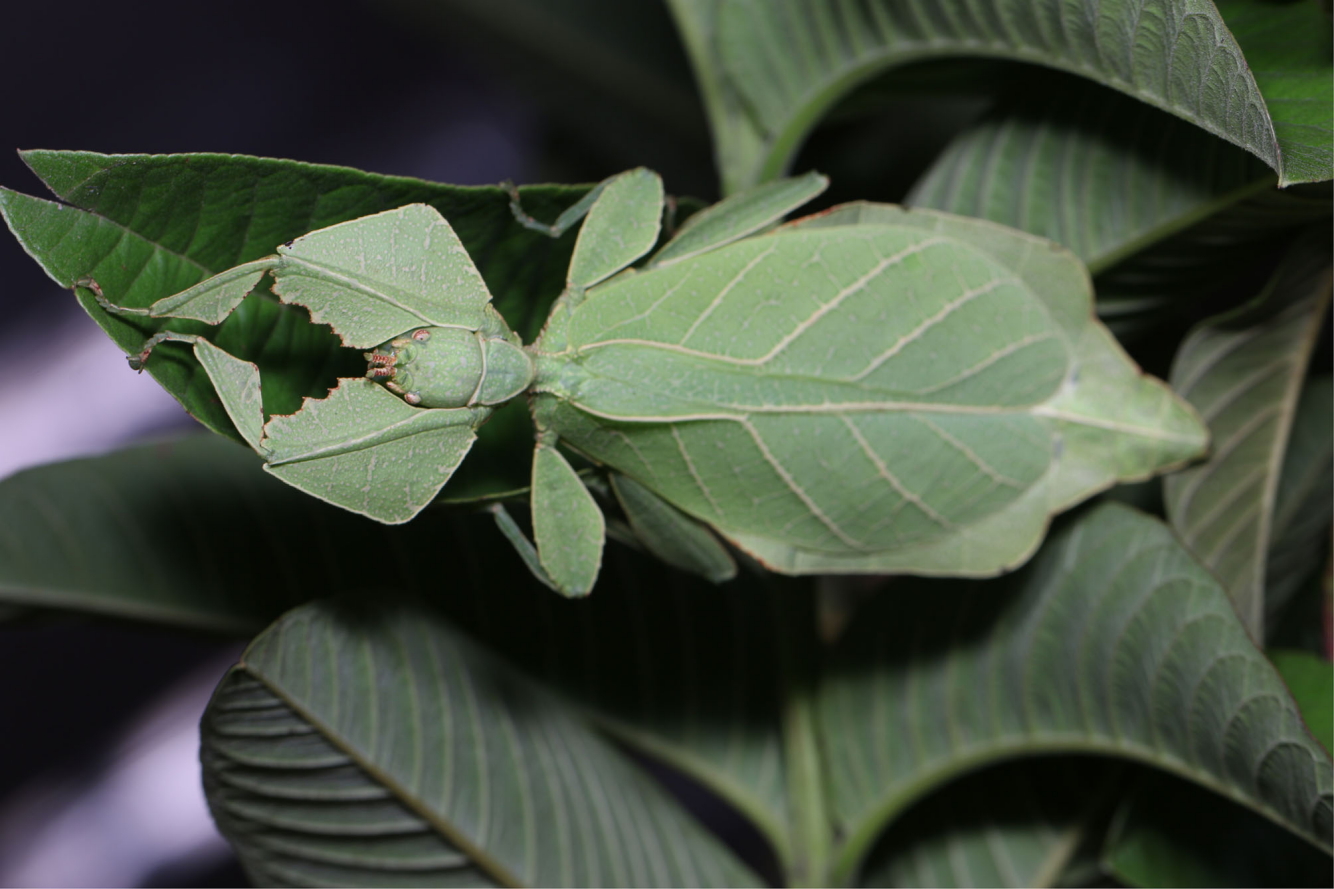
A female leaf insect (*Cryptophyllium westwoodii*). Photo by Zhiwei Dong.

A more fascinating example of masquerade is from leaf and stick insects in the order Phasmatodea, which mainly distribute in tropical regions and temperate regions [9, 10]. Phasmatodea species can mimic the shape of plants, disguising themselves as branches or leaves, making it difficult to detect them [11]. Very interestingly, among approximately 3,500 species of Phasmatodea (representing 21 families), the remarkable adaptation of simulating leaves is almost exclusively limited to the family Phylliidae, which contains just over 100 species, whereas most stick insects mimic branches [9, 12–14]. In 1889, Wallace reported on the phenomenon of leaf masquerade in insects, stating that “leaf insects (Phylliidae) can be considered the most perfect masquerade in the insect class” [3]. *Cryptophyllium westwoodii* (Fig. 1) is one of the representative species of the Phylliidae and disguises a nearly impeccable leaf masquerade with a leaf-like venation pattern and lobe-like extensions on the abdomen and legs [15, 16]. However, although the ecological and adaptive evolutionary significance of leaf-like masquerade is well known, little is known about the genetic basis of the origin and evolution of this complex phenotypic trait. Among them, in particular, the lack of reference genomes for representative species of leaf insects limits the exploration of the genetic mechanisms underlying this phenomenon.

In order to investigate the genomic basis of leaf morphology in leaf insects, we selected *C. westwoodii* (Fig. 1) as one representative species of Phylliidae to assemble its chromosome-level reference genome using Nanopore sequencing and Hi-C. Combined with karyotyping, we assembled 13 autosomes, 1 X sex chromosome, and 1 candidate B chromosome in this species. Combining comparative genomic and developmental transcriptome analyses, we found that some *Cuticle* genes, especially *resilin*, may play important roles in abdominal leaf-like development. In summary, the findings provide important genomic resource for investigating the evolution of the leaf-like masquerade and also offer new insights into the leaf-like masquerade in leaf insects.

## Methods

### Insects

*C. westwoodii* is from a population reared in Kunming, Yunnan, China, with *Rubus* sp. as host plant in our lab, and this population was established from eggs originally collected from Muang Fuang, Nang Ha, Laos, in 2017 [15]. 1 individual of the first instar was collected for sequencing on the Illumina platform for genome survey and correct errors. 1 male adult was sequenced on the Nanopore platform, and another male adult was collected for Hi-C sequencing for *de novo* chromosome-level genome assembly. Females and males were collected for karyotype analysis. The whole body of 1 female adult and 1 male adult and the laterally leaf-like abdominal expansions of 3 female individuals at 5 developmental stages were collected for transcriptomic sequencing.

### Karyotype analysis

Sexually mature individuals (both female and male) from *C. westwoodii* were used for the karyotype analysis, referring to the previously described method [17, 18] with some modifications. Colchicine (0.1%) was injected into the abdominal cavity of both female and male insects for 1 hour. Gonads were dissected from both female and male insects in 1× phosphate-buffered saline (PBS), with the surrounding connective tissue carefully removed, subjected to hypotonic treatment in 0.05% sodium citrate for 10 minutes, and fixed in Carnoy’s solution (methanol/acetic acid, 3:1) in 2 rounds, each lasting 25 minutes. After aspirating Carnoy’s solution, the fixed material was treated with a 60% solution of glacial acetic acid and then gently blown using a pipette tip to evenly distribute the tissues. Approximately 20–40 μL of turbid liquid was aspirated and quickly dropped onto a glass slide that had been stored frozen −4°C. The slides were dried in an oven at 42°C and then stained with 10% Giemsa solution for 1 hour. After discarding the dye solution, the slides were washed with ddH_2_O several times and air dried for 30 minutes. The slides were observed under a 630× light microscope (ZEISS, Axio Imager.D2), and the metaphase cells with well-dispersed chromosomes were selected for photography.

### Genome sequencing and survey

For Illumina sequencing, genomic DNA was isolated from the whole body of 1 larva individual using the Trelief Animal Genomic DNA Kit (TsingKe). Paired-end libraries with a 350-bp insert size were generated using NEB Next Ultra DNA Library Prep Kit and sequenced on the Illumina HiSeq4000 platform at Novogene. The raw reads containing >90% bases with a quality score below Q20 or >10% undetermined bases (Ns) were filtered out using Fastp (RRID:SCR_016962) (version 0.20.1) [19], and PCR duplicates within the paired-end reads were subsequently removed using FastUniq (RRID:SCR_000682) (version 1.1) [20]. The remaining clean reads were utilized for genome size estimation via *k*-mer (*k* = 17) analysis with kmerfreq (version 1.0) [21] and were subsequently employed for base-level polishing of the *de novo* genome assembly.

For Oxford Nanopore long-read sequencing, genomic DNA from another male adult was isolated to construct long DNA fragment libraries (NextOmics). Long DNA fragments were selected using the BluePippin system (Sage Science) and then attached to sequencing adapters using a Ligation Sequencing Kit (Oxford Nanopore, catalog number: SQK-LSK109). The quantified library fragments were then sequenced on a Nanopore PromethION sequencer (Oxford Nanopore Technologies) instrument at the Genome Center of Nextomics.

The sample treatment and the library construction for Hi-C sequencing followed the previously described protocol [22, 23]. The tissue samples from the whole body of a male adult were fixed, lysed, separated, and digested overnight with the restriction enzyme MboI. The Hi-C libraries with insert sizes of 200–300 bp were constructed by Covaris M220 and Dynabeads MyOne Streptavidin C1 (Thermo Fisher Scientific) and sequenced on the Illumina NovaSeq sequencing platform at Novogene.

### Transcriptome sequencing and transcriptomic analysis

For transcriptome sequencing, we collected the whole body of 1 female adult and 1 male adult, as well as the laterally leaf-like abdominal expansions of 3 female individuals at 5 developmental stages, including second, third, fifth, and seventh instar larvae and the eighth instar (adults). We extracted total RNA using the TRIzol reagent (Thermo Fisher Scientific). Paired-end libraries were constructed using the VAHTS RNA-seq V8 Library Prep Kit (Vazyme). These libraries were then sequenced on the Illumina NovaSeq 6000 platform for 150-bp paired-end reads. Trimmomatic (RRID:SCR_011848) (version 0.36) [24] was used to trim adapter sequences and filter low-quality bases from raw reads, with the following parameters: LEADING:3 TRAILING:3 SLIDINGWINDOW:4:15 MINLEN:40. The RNA sequencing clean reads was were aligned to the *C. westwoodii* genome by Hisat2 (RRID:SCR_015530) (version 2.2.1) [25] and then assembled using StringTie (RRID:SCR_016323) (version 2.1.7) [26]. The gene expression levels (transcripts per million; TPM) were quantified by StringTie (RRID:SCR_016323) (version 2.1.7) [26] based on the corresponding transcript annotation and the number of reads mapped to gene fragments. To cluster expression profiles over five developmental stages, all the differentially expressed genes (DEGs) were divided into the different clusters based on their mean normalized TPM values within the Mfuzz software (RRID:SCR_000523) (version 2.48.0) [27], and Gene Ontology (GO) enrichment analysis was performed for each cluster. The express trend line chart, the heatmap, and GO enrichment terms were visualized using the ClusterGVis package [28].

Genes differentially expressed in the laterally leaf-like abdominal expansions of female individuals at the five developmental stages were respectively identified as follows. Nonnormalized read counts for all detected genes were generated by StringTie (RRID:SCR_016323) (version 2.1.7) [26], and a read count table was subsequently compiled using the Python script “prepDE.py” from the StringTie package. Then, the differentially expressed genes (DEGs) were identified using DESeq2 (RRID:SCR_015687) (version 1.20.0) [29] based on its negative binomial generalized linear model. Finally, the DEGs in different groups were retained with a |log2(fold change)| > 2 and adjusted *P* < 0.05 (using the Benjamini–Hochberg algorithm).

### Genome assembly and chromosome construction

The draft contig assembly was generated using nextDenovo (RRID:SCR_025033) (version 2.5.0) [30] with Nanopore reads. To reduce redundancy, haplotigs and overlapping contigs were further purged based on read depth using purge_dups (RRID:SCR_021173) (version 1.2.3) [31]. Next, both the Illumina data and Nanopore reads were further used to polish the assembly using nextPolish (RRID:SCR_025232) (version 1.3.1) [32]. Then, the Hi-C paired-end reads were mapped to the polished assembly iteratively, and the resulting paired-end tags were filtered by JUICER (RRID:SCR_017226) (version 1.6) [33] based on the restriction fragment map. 3D-DNA software (RRID:SCR_017227) (version 180,922) [34] was used to order and orient contigs, yielding scaffolds. Finally, contig orientations were corrected and the suspicious fragments were moved to unanchored groups by manual visual inspection using JUICEBOX (version 1.11.08) [35].

### Quality assessment of genome assembly

The following 3 methods were used to evaluate the quality of the assembled genome. First, the Illumina reads and Nanopore reads were mapped to the chromosome-level genome assembly using BWA-men (RRID:SCR_010910) (version 0.7.11) [36] and minimap2 (RRID:SCR_018550) (version 5.1) [37]; then, the mapping ratio was calculated by SAMTOOLS (RRID:SCR_002105) (version 1.3.1) [38]. Second, the assembly indicators such as genome size, scaffold number, scaffold N50, GC content, and repetitive sequence content of the genome were calculated using custom scripts. Finally, BUSCO (RRID:SCR_015008) (version 5.2.2) [39] was run to assess the genome completeness based on the insecta_odb10 dataset [40].

### Repetitive sequence and protein-coding gene prediction

For repetitive sequence annotation, we used LTR_FINDER (RRID:SCR_015247) (version 1.05) [41] to identify long terminal repeat retrotransposons and TRF (RRID:SCR_022193) (version 4.09) [42] software to identify tandem repeats. Next, RepeatMasker (RRID:SCR_012954) (version 4.0.5) [43] was used to find transposable elements (TEs) by mapping sequences against the Repbase TE library at the DNA level, and RepeatProteinMask (RRID:SCR_012954) (version 4.0.6) [44] was used to identify TE-relevant proteins at the protein level. Subsequently, we used RepeatMasker (RRID:SCR_012954) (version 4.0.5) [43] through *de novo* repeat library build by RepeatModeler (RRID:SCR_015027) (version 1.0.4) [45] to *de novo* predict TEs.

We adopted a combination of homologous prediction, *de novo* prediction, and transcriptome prediction methods to annotate protein-coding genes. For homology-based predictions, the protein sequences of 3 termites (*Zootermopsis nevadensis* [GCA_000,696,155.1] [46], *Cryptotermes secundus* [GCA_002,891,405.2] [47], *Reticulitermes speratus* [GCA_021,605,165.1] [48]), 1 locust (*Schistocerca nitens* [GCA_023,898,315.2]), 2 model insects (*Drosophila melanogaster* [GCA_000,001,215.4] [49], *Tribolium castaneum* [GCA_000,002,335.3] [50]) from NCBI were aligned to the *C. westwoodii* genome using tblastn (RRID:SCR_011822) (version 2.2.26) [51] with an E-value cutoff of 1e-5, and the obtained BLAST hits were merged using Solar (RRID:SCR_000850) (version 0.9.6) [52] software. GeneWise (RRID:SCR_015054) (version 2.2.0) [53] was used to predict the complete gene structure based on the corresponding gene regions of each BLAST hit. For transcriptome prediction, the transcriptome of *C. westwoodii* was aligned using Hisat2 (RRID:SCR_015530) (version 2.2.1) [25] and then assembled using StringTie (RRID:SCR_016323) (version 2.1.7) [26]. The assembled transcriptome sequence was mapped to the genome for gene structural prediction using Transdecoder (RRID:SCR_017647) (version 5.5.0) [54] and PASA (RRID:SCR_014656) (version 2.3.3) [55]. For *de novo* prediction, the protein-coding gene sets from *C. secundus* and the assembled transcripts from *C. westwoodii* were used to train *ab initio* predicting models using Augustus (RRID:SCR_008417) (version 3.4.0) [56]. *De novo* predictions were performed using Augustus (RRID:SCR_008417) (version 3.4.0) [56] with *ab initio* models from *C. secundus, D. melanogaster*, and the transcriptome. A total of 10 gene sets from 3 prediction methods were merged to form a comprehensive and nonredundant gene set using EvidenceModeler (RRID:SCR_014659) (version 1.1.1) [57].

### Gene function annotation

The predicted protein-coding sequences were to aligned to 4 databases—TrEMBL (RRID:SCR_002380) [58], SwissProt (RRID:SCR_021164) [59], KEGG (RRID:SCR_012773) [60], and NR [61]—with an E-value cutoff of 1e-5 to obtain functional information using BLASTP (RRID:SCR_001010) (version 2.2.26) [51]. InterProScan (RRID:SCR_005829) (version 5.8.0) [62] software was used to search for known motifs and domains by mapping protein-coding sequences to Pfam (RRID:SCR_004726) (version 27.0), PRINTS (RRID:SCR_003412) (version 42.0), ProDom (RRID:SCR_006969) (version 2006.1), and SMART (RRID:SCR_005026) (version 6.2) databases.

### B chromosome structure and function analysis

To investigate the origin of the B chromosome in *C. westwoodii*, the collinearity relationship between 2 species (*C. westwoodii* and *Dryococelus australis* [GCA_029,891,345.1] [63]) and within *C. westwoodii* (B chromosome and other 14 A chromosomes) was established. According to the genome annotation file of *C. westwoodii* and *D. australis*, the protein sequences of all 15 chromosomes of *C. westwoodii* were aligned to the protein sequences of 17 chromosomes of *D. australis*, and the protein sequences of the B chromosome in *C. westwoodii* were aligned to the protein sequences of the other 14 A chromosomes in *C. westwoodii* by BLASTP (RRID:SCR_001010) (version 2.2.26) [51]. MCScanX (RRID:SCR_022067) (version 2.2.26) [64] software was searched for collinear blocks based on alignment information and annotated GFF files. The collinearity between 2 species (*C. westwoodii* and *D. australis*) and within *C. westwoodii* (B chromosome and the other 14 A chromosomes) was visualized using Circos (RRID:SCR_011798) (version 0.69–6) [65].

To compare the differences in structure and function between the B chromosome and the other 14 A chromosomes, the sequencing depth for each chromosome was calculated with a 100-kb sliding window using the BAM file generated from aligning the Nanopore reads to the assembly genome of *C. westwoodii*, and the proportion of repetitive sequence types for each chromosome was calculated. A scatterplot depicting the distribution of sequencing depth was generated based on depth values calculated for each chromosome using a 100-kb sliding window. GO enrichment analysis was performed using annotated genes on the B-chromosome, and the background gene set for this analysis was the complete set of annotated genes in *C. westwoodii*. Significantly enriched GO terms (false discovery rate [FDR]–adjusted *P* ≤ 0.05, Benjamini–Hochberg method) were retained for further interpretation.

### Identification of orthologous groups and phylogenetic analysis

To cluster families of protein-coding genes, we extracted protein sequences from *C. westwoodii* and the other 7 insects, including 2 stick insects (*Timema monikensis* [66], *D. australis* [GCA_029,891,345.1] [63]), 1 locust (*S. nitens* [GCA_023,898,315.2]), 1 cockroach (*Periplaneta americana* [GCA_025,594,305.2] [67]), 1 earwig (*Forficula auricularia* [68]), 1 aphid (*Aphis gossypii* [GCA_020,184,165.1] [69]), and 1 model insect (*D. melanogaster* [GCA_000,001,215.4] [49]). The protein sequences resulting from alternative splicing variants or containing premature stop codons were filtered from the set of protein-coding genes. OrthoFinder (RRID:SCR_017118) (version 2.5.2) [70] was used to search orthologous groups for protein sequences of 8 insect genomes. The protein sequences of 1:1 orthologs in all 8 species were aligned with MAFFT (RRID:SCR_011811) (version 7.487) [71] software, and then nonconservative and unreliable aligned areas were removed using trimAI (RRID:SCR_017334) (version 1.4) [72] with “-gt 0.5”. The filtered protein sequences of 1:1 orthologs were concatenated to generate a supermatrix (concatenated protein alignment). RAxML (RRID: SCR_006,086) (version 8.2.10) [73] software was used to construct a phylogenetic tree from the concatenated protein supermatrix of 8 species, using the “PROTGAMMAWAG” model with 100 bootstrap replicates. The mcmctree program of the PAML (RRID:SCR_014932) (version 4.8) [74] package was used to estimate species divergence times based on 4-fold degenerate synonymous sites (4dTV) extracted from 1:1 orthologs and 5 fossil calibration points from the TimeTree (RRID:SCR_021162) database [75] and fossil records [13, 76].

### Gene family, positively selected genes, and rapidly evolving genes

According to orthologous gene clusters and divergence times, Café (RRID:SCR_005983) (version 4.2.1) [77] was used to identify the expanded and contracted gene families in *C. westwoodii* with inputs from orthologous gene clusters identified by OrthoFinder (RRID:SCR_017118) (version 2.5.2) [70] and the phylogenetic tree with divergence times.

To analyze positively selected genes and rapidly evolving genes, the coding sequences of 1:1 orthologs in 8 species were extracted to align with PRANK (RRID:SCR_017228) (version 170,427) [78], and then aligned regions with gaps were removed using Gblocks (RRID:SCR_015945) (version 0.91b) [79] software with “-t=c -b5=n.” The PAML (RRID:SCR_014932) (version 4.8) [74] software, based on the branch-site and branch models, was used to identify positively selected genes and rapidly evolving genes, respectively. First, 1 ratio model was used to calculate the evolutionary rate ω (*Ka*/*Ks*) of each 1:1 ortholog in each species. The branch-site model was used to detect positive selection signals in genes of *C. westwoodii*. A likelihood ratio test (LRT) was used to compare the alternative hypothesis model that allowed sites to be under positive selection on the foreground branch (*C. westwoodii*) with a null hypothesis model that all sites were under purifying or neutral selection. The *P* value of each gene was calculated based on chi-square statistics, and genes with a *P* value less than 0.05 were defined as positively selected genes. Similarly, the branch model was used to detect rapid evolution genes. The LRT with *df* = 1 was performed to compare the fit of the null model, which assumes all branches have the same evolutionary rate, with the alternative model that allows the foreground branch to have a different evolutionary rate. Genes with *P* < 0.05 and a higher ω value for the foreground than the background branches were identified as rapidly evolving genes. KEGG and GO enrichment analyses were performed using DAVID (RRID:SCR_001881) (version 6.8) [80] and KOBAS (RRID:SCR_006350) (version 3.0) [81] for expanded gene families, contracted gene familie, positively selected genes, and rapidly evolving genes. For these enrichment analyses, the entire annotated genes of *C. westwoodii* served as the background gene set. Significant enriched Gene Ontology (GO) and Kyoto Encyclopedia of Genes and Genomes (KEGG) terms (FDR-adjusted *P* ≤ 0.05, Benjamini–Hochberg method) were retained.

### Genome-wide scanning identification of the *Cuticle* gene family

Genome-wide protein sequences of *C. westwoodii, T. monikensis, D. australis*, and *D. melanogaster* were extracted to scan for candidate *Cuticle* genes using hmmscan from HMMER (RRID:SCR_005305) (version 3.3.1) [82] software with the insect cuticle protein domain (pfam:PF00379.24). To distinguish and assign these candidate *Cuticle* genes to different *Cuticle* gene subfamilies, multiple alignments of protein sequences from the *Cuticle* genes were performed using MAFFT (RRID:SCR_011811) (version 7.487) [71], and the poorly aligned regions and partial gaps were removed with trimAI (RRID:SCR_017334) (version 1.4) [72] (gt = 0.5). Then, the filtered alignments were used to construct a phylogenetic tree by RAxML (RRID: SCR_006,086) (version 8.2.10) [73] software with options “-f a -x 12,345 -N 1000 -p 12,345 -m PROTGAMMAJTTX”. The phylogenetic tree was displayed and edited using FigTree (RRID:SCR_008515) (version 1.4.4) [83]. To further confirm the authenticity of candidate *Cuticle* genes, BLASTP (RRID:SCR_001010) [51] analyses on the NCBI webserver were used to examine functional information.

## Results

### Genome assembly

**Figure 2:**
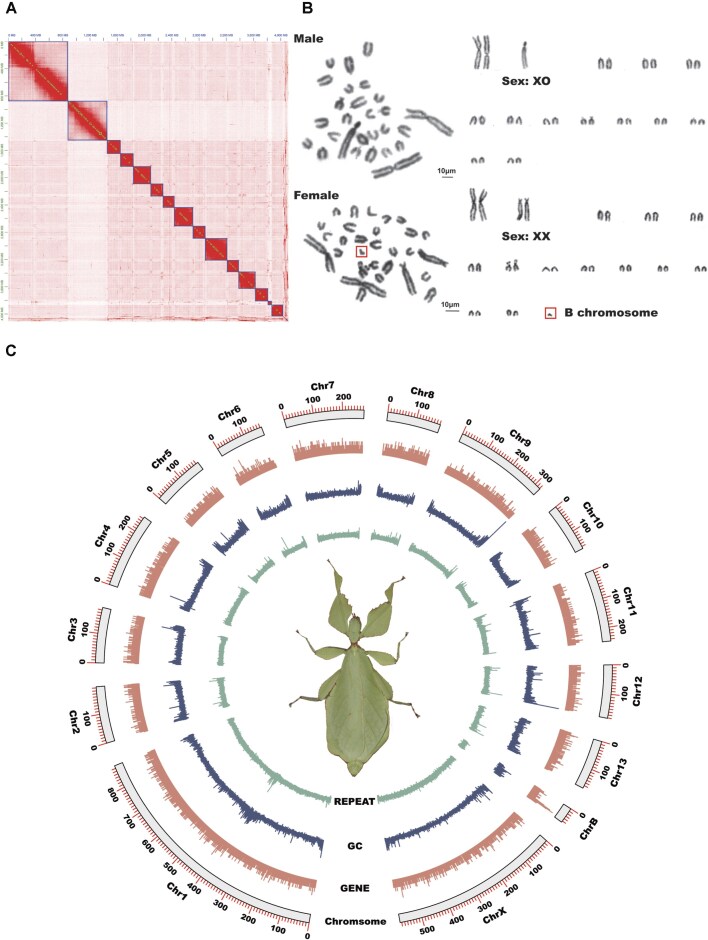
Genome description of *Cryptophyllium westwoodii*. (A) Hi-C interaction map produced by 3D-DNA. (B) The karyotype of female and male adults. During mitotic metaphase, 29 chromosomes (1 pair of X sex chromosomes, 1 B chromosome [denoted by red arrow], and 13 pairs of autosomes) were observed in females, and 27 chromosomes (1 X sex chromosome and 13 pairs of autosomes) were observed in males. (C) Circos plot of chromosome-level genome. Tracks represent the distribution of repetitive sequence density, GC density, gene density and chromosome length, respectively. Densities were calculated with a 100-kb sliding window.

A total of 246 Gb ONT long reads (61.5× coverage), 144 Gb Illumina short reads (36× coverage), and 716 Gb Hi-C reads (173× coverage) were generated to assembly a high-quality chromosome-level genome of *C. westwoodii* (Supplementary Table S1). Based on *k*-mer frequency analysis of Illumina short reads, the genome size was estimated to be 4.19 Gb with a heterozygosity rate of 0.7% (Supplementary Fig. S1; Supplementary Table S2). We first used ONT long reads for *de novo* assembly to obtain a draft contig-level assembly with a total size of 5.32 Gb, which consists of 5,039 contigs (N50 = 3.26 Mb) (Supplementary Table S3). Then, after removing the redundant sequences based on read depth of the draft contigs, we obtained an assembly with a reduced genome size of 4.09 Gb, comprising 1,665 contigs (N50 = 8.99 Mb) (Supplementary Table S3). After further polishing the haploid assembly twice with Illumina short reads and then scaffolding the contigs with Hi-C reads, we finally built a chromosome-level reference genome of *C. westwoodii*. Of the assembled genome sequences, 98.27% were successfully anchored in 15 pseudo-chromosomes, including 13 autosomes, 1 X sex chromosome, and 1 candidate B chromosome (Fig. 2A; Table 1). The cytogenetic analyses on cell metaphase confirmed the correctness of these anchored chromosomes and revealed the existence of 1 unpaired chromosome in the genome of this leaf insect (Fig. 2B). This chromosome-level assembly had a total length of 4.12 Gb with a scaffold N50 of 256.87 Mb, and and achieved 98.6% completeness of the BUSCO analysis, with 4.2% duplicated genes (Fig. 2C; Table 1; Supplementary Table S3). In addition, 93.56% and 99.94% of the chromosome-level genome were covered by Illumina reads (mapping rate: 94.75%) and by Nanopore reads (mapping rate: 99.98%), respectively (Table 1). These results indicate that the newly assembled genome of *C. westwoodii* has relatively high completeness and accuracy.

**Table 1:** The statistics of genome assembly and annotation in the *Cryptophyllium westwoodii* genome.

Features	*C. westwoodii*
Genome size (Gb)	4.12
Scaffold N50 (Mb)	256.76
Scaffold number	179
Chromosome number	15
Chromosome percent (%)	98.27
GC content (%)	40.51
Complete ratio of BUSCO (%)	98.6
Illumina reads mapping rate (%)	94.75
Nanopore reads mapping rate (%)	96.33
Repetitive sequences (%)	55.68
Number of protein-coding genes	19,131
Number of functional annotated genes	12,235

### Genome annotation

The annotation results for 2 classes of repetitive sequences (tandem repeats and TEs) were statistically analyzed, and a total of 2.29 Gb of repetitive sequences were annotated, accounting for approximately 55.68% of the assembled genome (Table 1). Among these, TEs in the *C. westwoodii* genome were composed of DNA transposons (DNA, 14.09%), long terminal repeat sequences (LTRs, 7.38%), long interspersed nuclear elements (LINEs, 5.15%), and short interspersed nuclear elements (SINEs, 3.40%) (Supplementary Table S5).

By integrating 10 gene sets obtained from 3 *ab initio* methods, 6 homology-based methods, and 1 transcriptome-based prediction method with different weight values, a set of the best gene structure was predicted, containing 19,131 protein-coding genes in the *C. westwoodii* genome (Table 1; Supplementary Table S6). The average lengths of genes, coding sequences (CDSs), exons, and introns were 70,747 bp, 1,366 bp, 203 bp, and 12,153 bp, respectively, while the average number of exons per gene was 6.71 (Supplementary Table S7). The BUSCO completeness of the annotated gene sets was 91.8% with 3.1% duplicated genes. For functional annotation, a total of 63.95% of predicted genes had informative matches in at least 1 of the functional protein databases (Table 1; Supplementary Table S8). Furthermore, 7,621 (49.78%) and 8,254 (53.92%) genes were successfully annotated with GO terms and mapped to KEGG pathways, respectively (Supplementary Table S8).

### The identification of B chromosome structure and function

**Figure 3:**
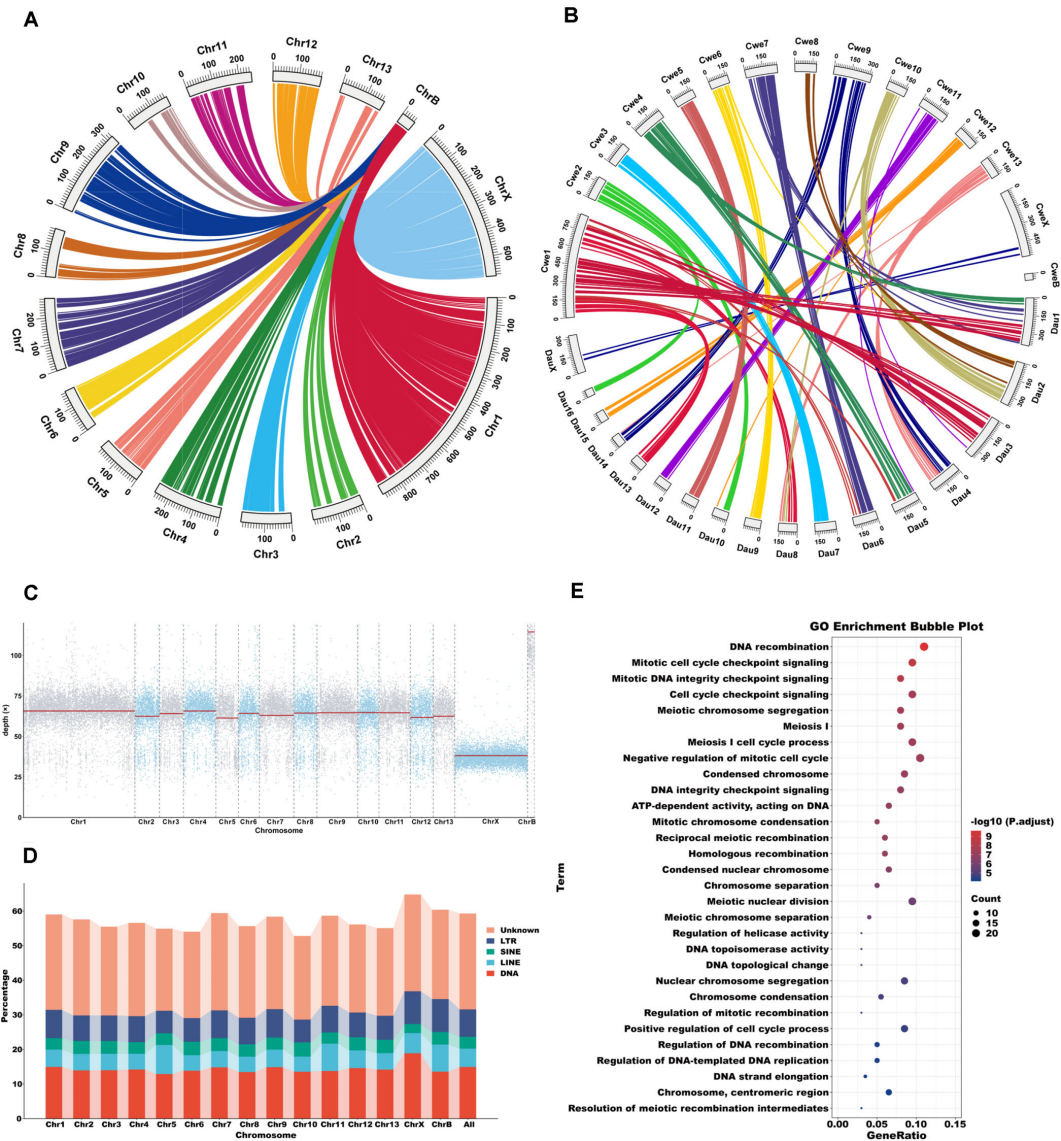
The structure and function of the B chromosome in *Cryptophyllium westwoodii*. (A) The synteny analysis between the B chromosome and the other 14 A chromosomes in the genome of *C. westwoodii*. (B) The chromosome synteny analysis between *C. westwoodii* (Cwe) and *D. australis* (Dau). (C) A scatterplot of the sequencing depth distribution for each chromosome. The plot was generated from Nanopore sequencing data of a male individual of *C. westwoodii* using a 100-kb sliding window. (D) The statistics of the proportion of repetitive sequence types for each chromosome in *C. westwoodii*. (E) GO enrichment analysis of the protein-coding genes on the B chromosome in *C. westwoodii*.

Our assembled data and cytogenetic analysis have suggested a candidate B chromosome in the *C. westwoodii* genome (Fig. 2A, B). To investigate the origin and evolution of the B chromosome, we first compared the collinearity relationship between the B chromosome and the other 14 A chromosomes in the *C. westwoodii* genome and detected many homologous gene fragments among them (Fig. 3A), suggesting that the B chromosome possibly originated from the 14 A chromosomes. We further compared the collinearity relationship of chromosomes between *C. westwoodii* and *D. australis* and found no any collinearity between the B chromosome of *C. westwoodii* and the chromosomes of *D. australis* (Fig. 3B).

We then investigated the structural characteristics of the B chromosome in the *C. westwoodii* genome. Our data showed that the mean depth of the B chromosome (107×) was nearly twice that of the autosomes (57–61×) (Fig. 3C; Supplementary Table S18), indicating the existence of multiple B chromosomes. Compared to the other A chromosomes, the B chromosome had the second highest proportion of repetitive sequences (60.41%) among all chromosomes, exceeded only by the X chromosome; moreover, LTR retrotransposons showed their highest proportion (9.53%) on the B chromosome (Fig. 3D; Supplementary Table S16). A total of 419 protein-coding genes were annotated on the B chromosome (Supplementary Table S19). GO enrichment analysis revealed that these genes were significantly enriched in functions related to DNA replication and recombination, cell cycle checkpoint signaling, and chromosome condensation (Fig. 3E; Supplementary Table S17), which are key processes for genome integrity and chromosome transmission.

### Phylogenetic analyses and ortholog identification

**Figure 4:**
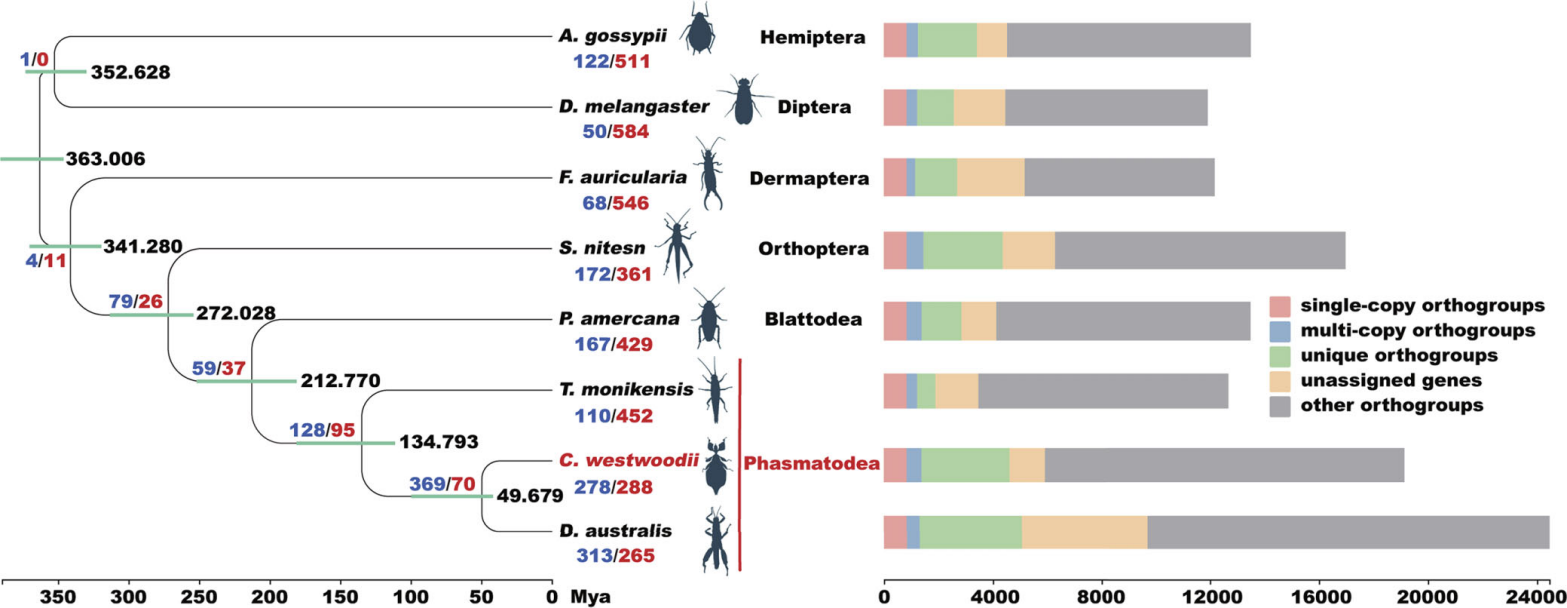
Phylogenetic and evolutionary analyses of the *Cryptophyllium westwoodii* genome. The left panel shows a phylogenetic tree with branch labels representing expanded gene families (blue), contracted gene families (red), and divergence times (black), respectively, for each clade. The right panel displays bar plots of orthogroup (gene families) counts, in which colors correspond to distinct categories.

Phylogenomic analysis was performed to test the phylogenetic position of *C. westwoodii*. We compared the protein-coding genes of *C. westwoodii* with the other 7 insects (2 stick insects, 1 locust, 1 cockroach, 1 earwig, 1 aphid, and 1 fruit fly). Using OrthoFinder, we identified a total of 124,281 genes among the 8 species, of which 108,069 were clustered into 12,724 orthogroups. We also summarized single-copy orthogroups, multi-copy orthogroups, species-specific orthogroups (unique orthogroups), unassigned genes, and other orthogroups for each species (Fig. 4). In total, 841 single-copy orthogroups and 3,634 multi-copy orthogroups were identified among 8 species (Supplementary Table S9). In the *C. westwoodii* genome, 1,299 genes lacking orthologous relationships with the other 7 species were clustered into 588 unique orthogroups (Supplementary Table S9).

To investigate the genomic evolution of *C. westwoodii*, we reconstructed a phylogenomic tree for the 8 species based on 841 single-copy orthologs (535,158 amino acids sites). The phylogenetic relationships of 8 species were well resolved, with all the nodes strongly supported (UFB/SH-aLRT = 100/100), indicating a high resolution in the phylogram (Supplementary Fig. S3). Meanwhile, divergence time estimation based on the 4-fold degenerate synonymous sites (4dTV) of the 841 single-copy orthologs and 5 fossil calibration points (Supplementary Table S20) indicated that the ancestors of *C. westwoodii* and *D. australis* diverged ~49.67 million years ago (Mya) (Fig. 4).

### Positively selected genes and rapidly evolving genes

We identified positively selected genes and rapidly evolving genes from 841 single-copy orthologs across these 8 species using the branch-site and branch models, respectively. In *C. westwoodii* genome, we found 153 positively selected genes and 354 rapidly evolving genes (*P* < 0.05), of which 97 genes were shared (Supplementary Tables S10–S11). Gene function annotation showed that these genes were involved in the regulation of signaling pathways (e.g., Wnt, EGFR, Notch, Hippo, and Hedgehog), epithelial cell polarization, the development and growth of muscle and eye pigment (Supplementary Tables S10–S11). Among them, *garnet* [84, 85] and *ruby* [86], related to pigment transport and deposition in compound eyes, were both positively selected and rapidly evolving genes. The proteins encoded by these 2 genes, together with those proteins encoded by *orange* and *carmine* genes, form the adapter protein 3 (AP-3) complex, which transports pigment particles into organelles within cells [87].

### Gene family expansion and contraction

**Figure 5:**
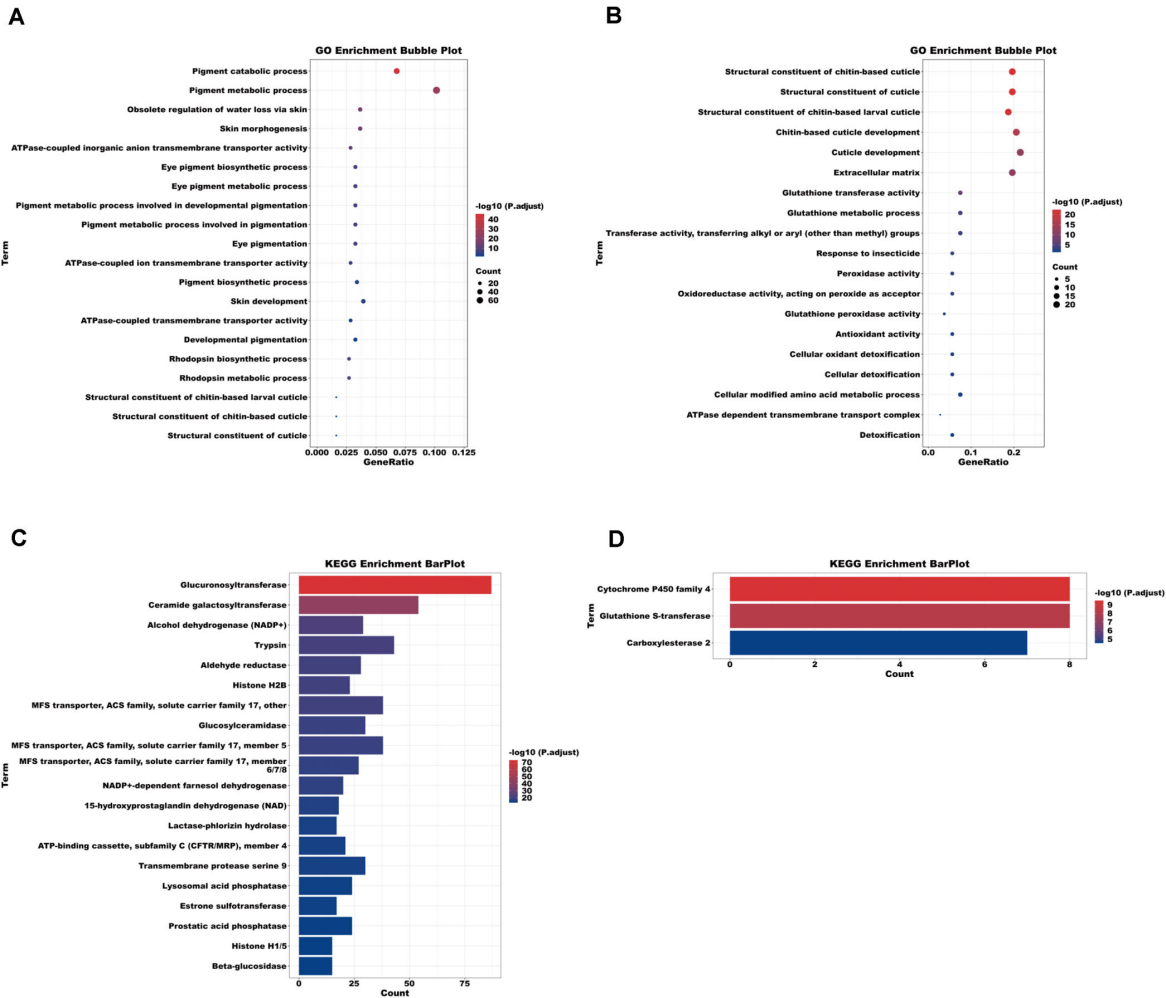
Enrichment analysis of gene families of *Cryptophyllium westwoodii*. (A) GO enrichment analysis of expanded gene families. (B) GO enrichment analysis of contracted gene families. (C) KEGG enrichment analysis of expanded gene families. (D) KEGG enrichment analysis of contracted gene families.

We used Café to study the expansions and contractions of gene families during the evolution of *C. westwoodii*. In the *C. westwoodii* genome, 278 expanded and 288 contracted gene families were identified (Fig. 4). GO enrichment analysis revealed that expanded gene families in *C. westwoodii* were enriched in the structural constituents of cuticle, skin development, eye pigmentation, and pigment biosynthetic process, while contracted gene families were enriched in extracellular matrix, detoxification, and cuticle development (Fig. 5A, B; Supplementary Tables S12–S13). In addition, the results of KEGG enrichment analysis showed that expanded gene families were enriched in glucuronosyltransferase, cytochrome P450 family 2 subfamily D, and cytochrome P450 family 2 subfamily J, while contracted gene families were enriched in cytochrome P450 family 4, glutathione S-transferase, and carboxylesterase 2 (Fig. 5C, D; Supplementary Tables S14–S15). These digestion- and detoxification-related KEGG classifications, such as cytochrome P450s, glucuronosyltransferase, and trypsin, have a direct effect on the digestion of plant cell walls, as well as detoxification and antibacterial properties [88–90].

### DEGs in the laterally leaf-like abdominal expansions of female individuals at 5 developmental stages

Transcriptome sequencing of the 15 samples at 5 developmental stages (second, third, fifth, seventh, and eighth [adults] instar) generated 101.88 Gb of clean data, with the Q30 scores ranging from 92.25% to 95.24%, GC content ranging from 43.68% to 50.77%, and mapping ratios ranging from 80.77% to 96.24% (Supplementary Table S21). Using the StringTie assembly program, we assembled 158,199 transcripts from the transcriptome data. Of these, 93,442 were predicted to have open reading frames by TransDecoder, yielding 29,238 genes, including 15,588 newly assembled ones.

**Figure 6:**
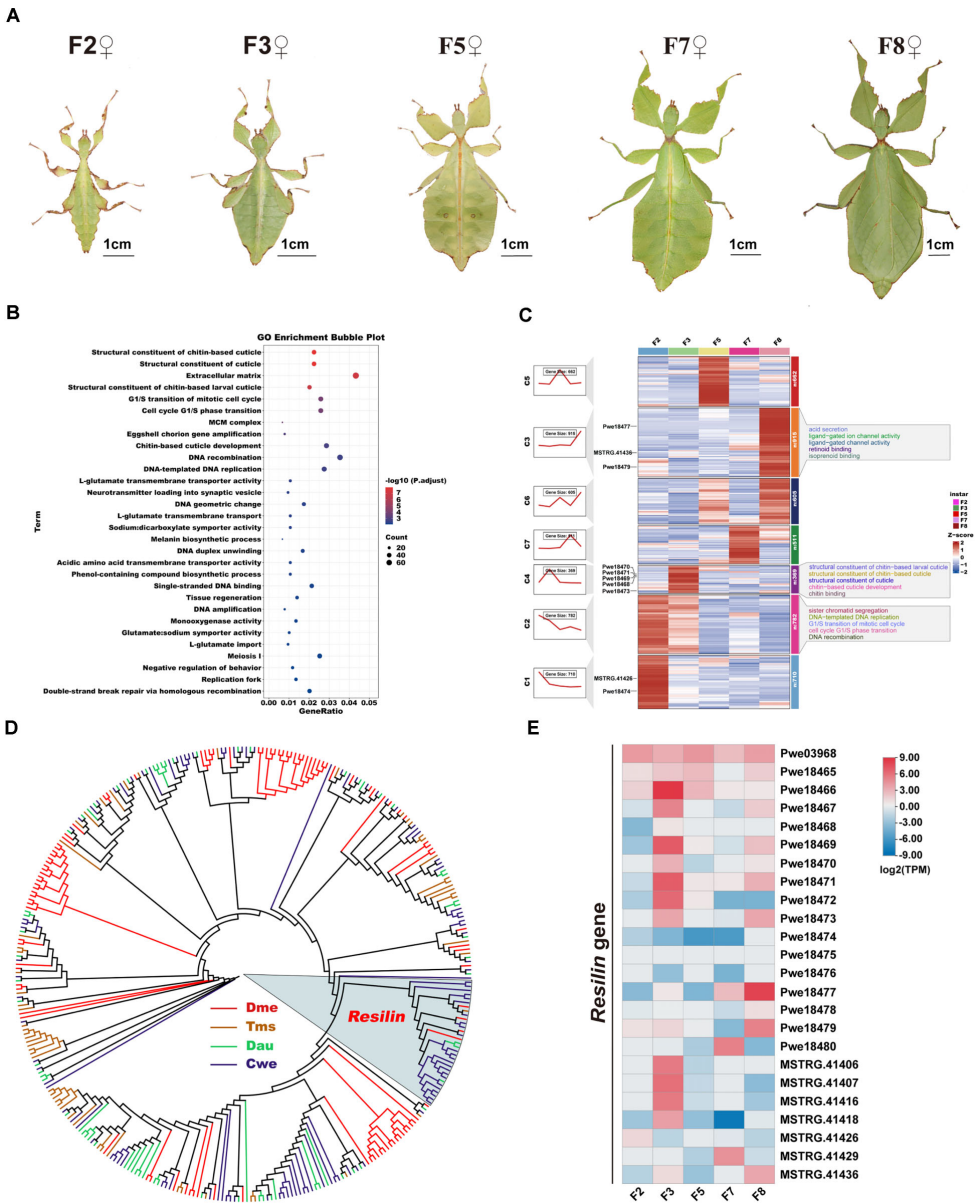
Differentially expressed genes at 5 different developmental stages of *Cryptophyllium westwoodi*. (A) Habitus of female individuals at the 5 different developmental stages for transcriptome sequencing. Photos by Zhiwei Dong. (B) GO enrichment analysis of 4,554 DEGs in the laterally leaf-like abdominal expansions of female individuals at the 5 different developmental stages in (A). (C) The clustering analysis of expression trends among 4,454 DEGs. The figure consists of a line chart, heatmap, and GO enrichment terms. The line chart and heatmap show the gene expression trend and level in each cluster. The heatmap is flanked by the IDs for the differentially expressed *resilin* copies on the left and the top 5 significant GO terms in 3 clusters on the right. (D) Phylogenetic analysis of *Cuticle* gene family among *D. melanogaster* (Dme), *T. monikensis* (Tms), *D. australis* (Dau), and *C. westwoodii* (Cwe). (E) Heatmaps of the *resilin* gene with 24 copies in the laterally leaf-like abdominal expansions at the 5 developmental stages. F2: second instar larvae; F3: third instar larvae; F5: fifth instar larvae; F7: seventh instar larvae; F8: eighth instar (adults). The red and blue colors in (C) and (E) indicate high and low expression levels, respectively.

To identify the DEGs in the laterally leaf-like abdominal expansions of female individuals at 5 developmental stages (second, third, fifth, seventh, and eighth [adults] instar), a total of 10 groups (pairwise comparisons) (Fig. 6A) were conducted, identifying a total of 4,554 differentially expressed genes. Among them, most DEGs were identified in the group of the second instar versus the eighth instar, while the fewest DEGs were detected in group of the second instar versus the third instar (Supplementary Fig. S4). GO enrichment analysis revealed that the top 10 GO terms of these DEGs are mainly related to the structural constituent of chitin-based cuticle and chitin-based cuticle development (Fig. 6B; Supplementary Table S22). These 4,554 DEGs were divided into 7 expression clusters (C1–C7) (Fig. 6C; Supplementary Table S23). Notably, cluster C4 (369 genes) was related to the structural constituent of chitin-based cuticle and chitin-based cuticle development, exhibiting a significant expression peak specifically during the third instar (Fig. 6C; Supplementary Table S24). Interestingly, expanded gene families and contracted gene families in the *C. westwoodii* genome were also enriched in the structural constituent of cuticle and cuticle development (Fig. 5A, B). The findings highlight a central role for *Cuticle* genes in the laterally leaf-like abdominal expansions of female individuals at the 5 developmental stages at both the genome and expression levels. To further investigate the role of *Cuticle* genes in morphological development of the leaf insect, we identified *Cuticle* genes in *C. westwoodii* through genome and transcriptome scanning. In total, 113 *Cuticle* genes were identified in the genome of the leaf insect (Fig. 6D). In these *Cuticle* genes, nearly half (53 genes) were identified to be differentially expressed at 5 developmental stages (Supplementary Tables S25–S26; Supplementary Fig. S5). In particular, the *Cuticle* gene called *resilin*, with 24 copies, was significantly expanded in *C. westwoodii* (Fig. 6D), and 10 copies of *resilin* were significantly differentially expressed at 5 developmental stages (Fig. 6E; Supplementary Tables S25–S26). Among 10 differentially expressed copies of *resilin*, 5 copies were divided into cluster 4 (C4), 3 copies into cluster 3 (C3), and 2 copies into cluster 1 (C1) (Fig. 6C). 2 copies of *resilin* were significantly upregulated from the second instar to the third instar during the abdominal expansion and swelling. Therefore, *Cuticle* genes, particularly *resilin*, exhibit stage-specific expression patterns during the developmental process of leaf masquerade in *C. westwoodii*.

## Discussion

Leaf insects in the family Phylliidae are the best model for studying the evolution of leaf masquerade because of their leaf-like bodies and extended legs. However, the lack of their genomic resources has limited investigation of genetic mechanism of this phenomenon. Here, we assembled a high-quality genome of *C. westwoodii*, representing the first chromosome-level assembly in the family Phylliidae. Compared with previously published genomes of walking sticks of the order Phasmatodea [63, 66, 91–94], the chromosome-level assembly of *C. westwoodii* represents the highest-quality assembly with the second highest N50 value (N50 = 256.87 Mb) and the lowest number of scaffolds (n = 179) (Supplementary Table S4). The assembled genome size of *C. westwoodii* is 4.09 Gb, which is the largest one in the published genomes of the order Phasmatodea (Supplementary Table S4). The repetitive elements occupy 55.86% of *C. westwoodii* genome, a proportion that falls within the range reported for the published genomes (49.29%–63.52%) (Supplementary Table S4). The gene features (the length of mRNA, CDSs, exons, and the number of exons) of the assembled *C. westwoodii* genome are similar to those of the other 4 stick insects (Supplementary Fig. S2), suggesting the reliability of gene annotation for *C. westwoodii*.

The B chromosome is a kind of extra or redundant or nonessential chromosome that lacks the ability to recombine and pair with autosomes (A chromosomes) [95]. It is estimated that approximately 15% of eukaryotic species harbor B chromosomes, with plants as major carriers, followed by insects [96, 97]. However, the complete and high-quality assembly of B chromosome in insects is still very rare. Interestingly, an unpaired single chromosome (54,774,726 bp) was assembled in the *C. westwoodii* genome (Fig. 1A), and combined with the results of karyotyping, we identified 1 unpaired single chromosome in the female (Fig. 1B), suggesting that B chromosomes have occurred in *C. westwoodii*. The B chromosome usually contains large repetitive sequences (DNA repeats and transposons) in essence [98]. Nevertheless, the proportion of repetitive sequences showed no significant difference between the B chromosome and other A chromosomes in *C. westwoodii*, suggesting that the proportion of repetitive sequences is not an indicator to distinguish the B chromosome from A chromosomes, but the high-density distribution area of repetitive and transposable sequences containing multiple candidate centromere regions within the B chromosome may be the evidence for mediating chromosome breakage and fusion to produce the B chromosome [99]. Besides, the B chromosome of *C. westwoodii* contains 419 genes, and the results of GO enrichment analysis showed that these genes were enriched in DNA replication and recombination, cell cycle checkpoint signaling, and chromosome condensation. In the B chromosomes of 2 locusts, *Abracris flavolineata* [100] and *Eyprepocnemis plorans* [101], genes directly involved in chromosome formation and cell cycle regulation have also been identified. These functional genes are not limited to insects; they also have been identified in mammals such as red fox and Chinese raccoon dog [102], lizard (*Anolis carolinensis*) [103], and fishes, including *Astyanax mexicanus, Astyanax correntinus*, and *Astatotilapia latifasciata* [100, 104, 105]. The studies have shown that these genes encoding cell cycle and chromosome-related functions (e.g., histones, DNA binding, and packaging proteins) may affect B chromosome precursor DNA derived from A chromosome genomic DNA through transposition, replication, or rearrangement events to form B chromatin and its reorganization [98–100]. Therefore, these genes may be critical for the survival and transmission of the B chromosome at the early evolutionary stage. In this study, we found some genes that have not been reported on the B chromosome, such as glycoside hydrolase family 28 (the plant cell wall digestion enzymes) [106, 107], gustatory receptor 26b (gustatory receptor) [108, 109], and cytochrome P450 6j1 (detoxification) [89] (Supplementary Table S19). This finding suggests that most genes on B chromosomes across different species are functionally enriched for cell cycle and chromosome condensation, but each species can also harbor a unique subset of genes with distinct functions.

Regarding the evolutionary origin of the B chromosome, a concept proposed based on cytogenetic methods is that the B chromosome is derived from A chromosomes. In *D. melanogaster*, B chromosome is thought to have originated from a single A chromosome [110], possibly triggered by a mitotic error involving chromosome 4 [111]. In contrast, most genes on the B chromosome of *C. westwoodii* were highly homologous to those in A chromosomes but were not aligned to any genes on the chromosomes of *D. australis*. This result is consistent with the results of maize [112, 113], rye [114], and *Phragmites australis* [99, 115], indicating that the B chromosome may have a mosaic origin based on homologous gene fragments from A chromosomes.

Like other leaf insects in the family Phylliidae, *C. westwoodii* have a unique abdominal structure with extensions on both terga and sterna [116]. Fossil evidence has indicated that the Middle Jurassic stick insect (*Aclistophasma echinulatum*) [117] and the Mesozoic stick insect (*Elasmophasma stictum*) [118] both showed abdominal extensions on terga, which is considered an initial manifestation of leaf-like extension in stick insects. In addition, the first fossil leaf insect (*E. messelense*) [119] presented extensions on the abdominal terga and sterna, similar to those of modern leaf insects [120]. Furthermore, extant leaf insect *C. westwoodii* has not only abdominal extensions but also thoracic and legged extensions [116]. These data indicate that the origin of abdominal extensions predates other modifications in Phasmatodea [117]. Thus, we focused on abdominal expansions at 5 developmental stages to explore the genetic mechanism of the leaf-like masquerade by integrating genomic and transcriptomic data. Interestingly, our comparative genomics demonstrated expanded and contracted gene families were functionally enriched in the structural constituent of cuticle and skin development, which may affect the development of the cuticle and epidermis in *C. westwoodii*. (Fig. 5A, B; Supplementary Tables S12–S13). Furthermore, transcriptomic data indicated that DEGs at different developmental stages were mainly enriched in the structural constituent of chitin-based cuticle and chitin-based cuticle development (Fig. 6B; Supplementary Table S22). These findings suggest that *Cuticle* genes may play important roles in the evolution of leaf-like abdominal extension at both the genome and expression levels. Cuticular proteins (CPs), as the principal components of the insect cuticle, interact with chitin fibers to form a Bouligand-like structure, playing a crucial role in shaping the body morphology during insect development and the construction of the external important parts and organs of the insect body [121–123]. For example, in orchid mantis, the cuticle is the major structural constituent of petal-like femoral lobes on the femur of the mid and hind legs, and the extensions of ventral femur are regulated by *Cuticle* genes through the Wnt signaling pathway [124]. Thus, we further scrutinized *Cuticle* genes in the genome of *C. westwoodii*. Our data revealed that 53 of 113 *Cuticle* genes in the genome of *C. westwoodii* showed significantly different expression at 5 developmental stages.

In particular, the *Cuticle* gene called *resilin* was significantly expanded in *C. westwoodii*, with 24 copies identified, of which 10 were significantly differentially expressed in the laterally leaf-like abdominal expansions at 5 developmental stages. Among 10 differentially expressed copies of *resilin*, 5 copies were divided into cluster 4 (C4), suggesting that the third instar could be an important time node for *Cuticle* genes. At this stage from the second to the third instar, 2 copies of *resilin* were significantly differentially upregulated, alongside the marked enlargement of abdominal extensions and the initial establishment of the leaf-like morphology. *Resilin* is a structural elastic protein widely distributed in insect exoskeletons, playing a crucial role in their movement and ecological adaptability [125, 126]. A resilin-bearing extensor ligament of legs, wing hinge, and wings is involved in jumping and flight [127, 128]. Meanwhile, *resilin* mutations decrease the attachment ability of adhesive pads and lead to slip, which is extremely detrimental to survival adaptability [129]. In addition, resilin-containing exoskeleton structures also exist in the abdominal cuticle of honey ant workers and queen termites, which seem to assist in the extension of the abdomen of queen termites during physogastry and support the expansion and contraction of the abdomen of *Myrmecocystus mexicanus* during fluid storage [130, 131]. These data suggest that *resilin* may promote the abdominal extensions and the formation of leaf-like bodies.

## Supplementary Material

giag022_Supplemental_Material

giag022_Authors_Response_To_Reviewer_Comments_original_submission

giag022_GIGA-D-25-00406_original_submission

giag022_GIGA-D-25-00406_Revision_1

giag022_Reviewer_1_Report_original_submission Reviewer 1 -- 10/23/2025

giag022_Reviewer_1_Report_revision_1 Reviewer 1 -- 1/11/2026

giag022_Reviewer_2_Report_original_submission Reviewer 2 -- 11/24/2025

giag022_Reviewer_2_Report_revision_1 Reviewer 2 -- 1/25/2026

## Data Availability

Genomic assembly sequences and the raw sequencing data (including Nanopore long reads, Illumina short reads, Hi-C reads, and RNA-seq reads) of *C. westwoodii* in this study have been submitted to the NCBI Database and can be accessed with Bioproject numbers PRJNA1314718 and PRJNA682332. All additional supporting data are available in the *GigaScience* repository, GigaDB [132].

## Additional Files

**Supplementary Table S1**. The statistics of sequencing data used for the *Cryptophyllium westwoodii* genome assembly.

**Supplementary Table S2**. Estimation of genome size using kmerfreq software.

**Supplementary Table S3**. The statistics for the *Cryptophyllium westwoodii* genome assembly based on different kinds of sequencing data.

**Supplementary Table S4**. The comparison of assembly metrics among *Cryptophyllium westwoodii* and the other 5 stick insects of the order Phasmatodea.

**Supplementary Table S5**. The statistics of annotated repetitive sequences in the *Cryptophyllium westwoodii* genome.

**Supplementary Table S6**. The evaluation of different types of annotated gene sets in EVM under weight values.

**Supplementary Table S7**. The statistics of protein-coding genes in the *Cryptophyllium westwoodii* genome.

**Supplementary Table S8**. The statistics of functional annotation of the predicted protein-coding genes in the *Cryptophyllium westwoodii* genome.

**Supplementary Table S9**. The statistics of 5 orthogroup categories across 8 species.

**Supplementary Table S10**. The statistics of positively selected genes of *Cryptophyllium westwoodii*.

**Supplementary Table S11**. The statistics of rapidly evolving genes of *Cryptophyllium westwoodii*.

**Supplementary Table S12**. GO enrichment analysis of expanded gene families of *Cryptophyllium westwoodii*.

**Supplementary Table S13**. GO enrichment analysis of contracted gene families of *Cryptophyllium westwoodii*.

**Supplementary Table S14**. KEGG enrichment analysis of expanded gene families of *Cryptophyllium westwoodii*.

**Supplementary Table S15**. KEGG enrichment analysis of contracted gene families of *Cryptophyllium westwoodii*.

**Supplementary Table S16**. The statistics of annotated repetitive sequences among 15 chromosomes in the *Cryptophyllium westwoodii* genome.

**Supplementary Table S17**. GO enrichment terms significantly among the genes on the B-chromosome.

**Supplementary Table S18**. The statistical data of each chromosome covered by Nanopore reads sequenced from a male individual in the genome of *Cryptophyllium westwoodii*.

**Supplementary Table S19**. The statistics of 419 genes on the B-chromosome.

**Supplementary Table S20**. Information of the calibration nodes used for calculating the divergence time.

**Supplementary Table S21**. The statistics of quality of RNA-seq raw data.

**Supplementary Table S22**. GO enrichment terms among the significantly differentially expressed genes in the laterally leaf-like abdominal expansions of female individuals at 5 developmental stages.

**Supplementary Table S23**. The statistics of expression trend clustering among 4,554 DEGs at 5 developmental stages.

**Supplementary table S24**. GO enrichment terms among the genes in Cluster2, Cluster3, and Cluster4.

**Supplementary Table S25**. Expression profiles of *Cuticle* genes at the 5 developmental stages.

**Supplementary Table S26**. Significantly differentially expressed *Cuticle* genes at the 5 developmental stages.

**Supplementary Fig. S1**. The statistics of *k*-mer analysis of the *Cryptophyllium westwoodii* genome. The first peak appearing at a depth of 20-fold is a heterozygous peak, whereas the third peak appearing at a depth of 74-fold corresponds to a repeated peak. The second peak appearing at a depth of 48-fold is homozygosity, and the predicted genome size is 4,199.09 Mb, with repetitive sequences accounting for 75.16% and heterozygosity accounting for 0.7%. The x-axis is depth (×), and the y-axis is the ratio of the frequency at each depth to the total frequency across all depths.

**Supplementary Fig. S2**. Comparisons of gene features among the genomes of 5 species in order Phasmatodea.

**Supplementary Fig. S3**. Phylogenetic relationship among the 8 species inferred by the amino acid sequences of the 841 single-copy genes.

**Supplementary Fig. S4**. The number of differentially expressed genes (DEGs) in the laterally leaf-like abdominal expansions of female individuals at the 5 different developmental stages.

**Supplementary Fig. S5**. The number of differentially expressed *Cuticle* genes in the laterally leaf-like abdominal expansions of female individuals at the 5 different developmental stages.

## Abbreviations

BLAST: Basic Local Alignment Search Tool; BUSCO: Benchmarking Universal Single-Copy Ortholog; CDS: coding sequence; DEG: differentially expressed gene; EGFR: epidermal growth factor receptor; 4dTV: 4-fold degenerate synonymous sites; Gb: gigabase; GO: Gene Ontology; Hi-C: high-throughput chromosome conformation capture; KEGG: Kyoto Encyclopedia of Genes and Genomes; LINE: long interspersed nuclear element; LTR: long terminal repeat sequence; Mb: megabase; Mya: million years ago; NCBI: National Center for Biotechnology Information; NR: nonredundant protein database; PBS: phosphate-buffered saline; SINE: short interspersed nuclear element; TE: transposable element; Wnt: wingless.
