# Supplementary material for: Chromosome-level genome assembly and transcriptomes of the leaf insect Cryptophyllium westwoodii provide insights into the evolution of leaf-like masquerade
Source: Gigascience. 2026 Mar 2;15:giag022. doi: 10.1093/gigascience/giag022 (PMC13108252; doi:10.1093/gigascience/giag022)
Supplement: giag022_Supplemental_Material [file giag022_supplemental_material.zip › GIGA-D-25-00406-R3_Supplementary Figure S1-S5.docx]

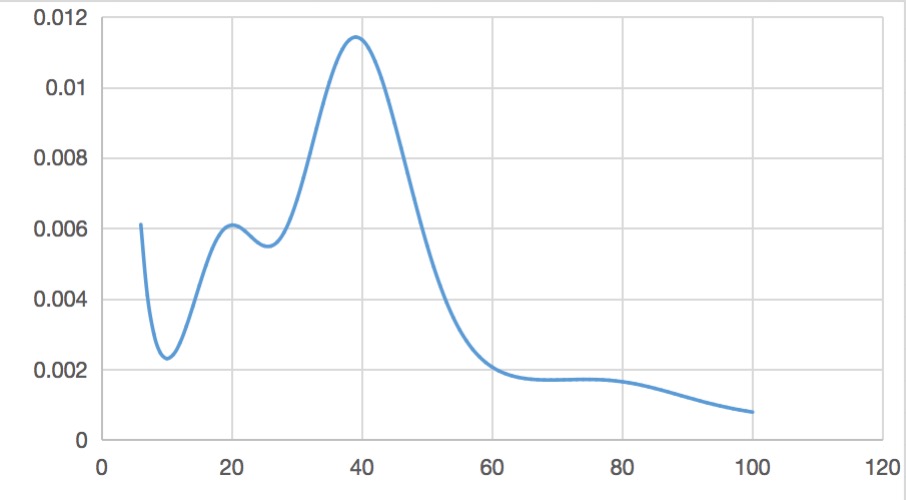


**Figure S1. The statistics of *k-mer* analysis of** ***Cryptophyllium westwoodii* genome.** The first peak appearing at a depth of 20-fold is a heterozygous peak whereas the third peak appearing at a depth of 74-fold corresponds to a repeated peak. The second peak appearing at a depth of 48-fold is homozygosity, and the predicted genome size is 4199.09 Mb, with repetitive sequences accounting for 75.16% and heterozygosity accounting for 0.7%. The x-axis is depth (×), and the y-axis is the ratio of the frequency at each depth to the total frequency across all depths.


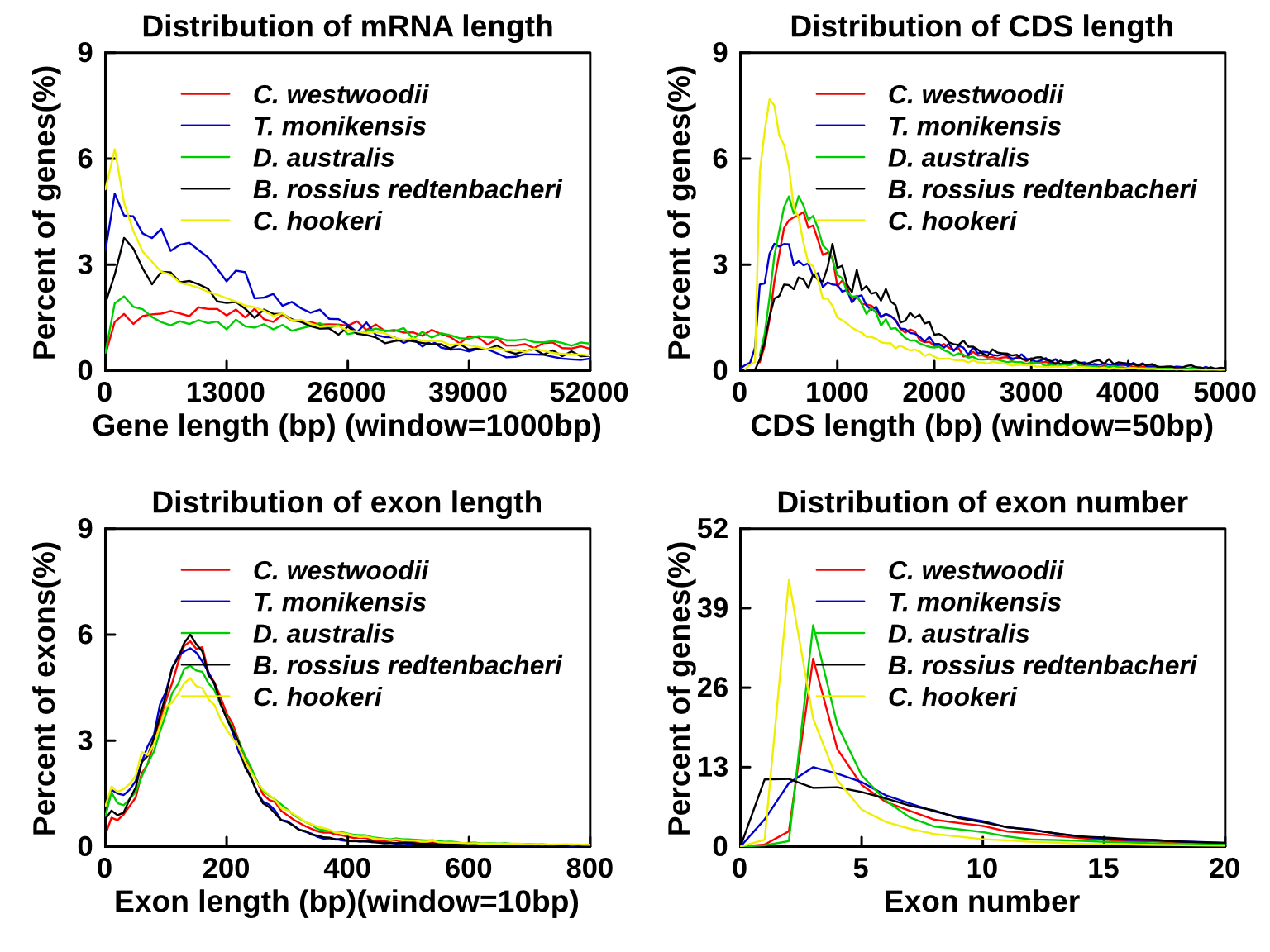


**Figure S2. Comparisons of gene features among the genomes of five species in order Phasmatodea.**

**
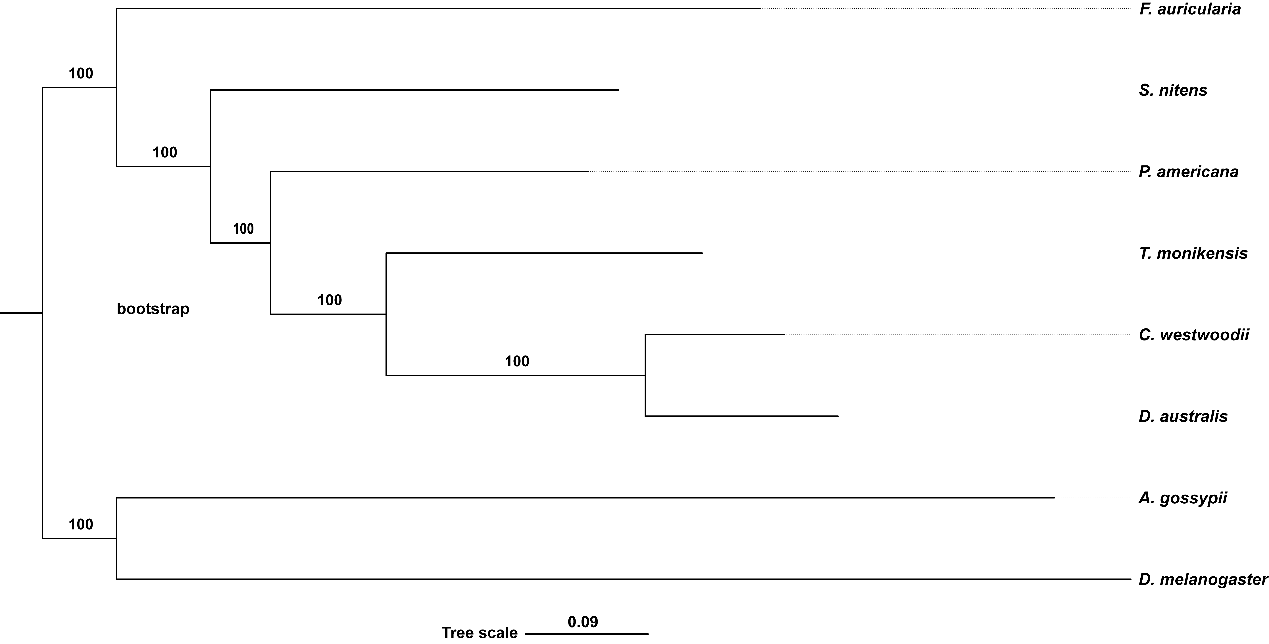
**

**Figure S3. Phylogenetic relationship among the eight species inferred by the amino acid sequences of the 841 single-copy genes.**

**
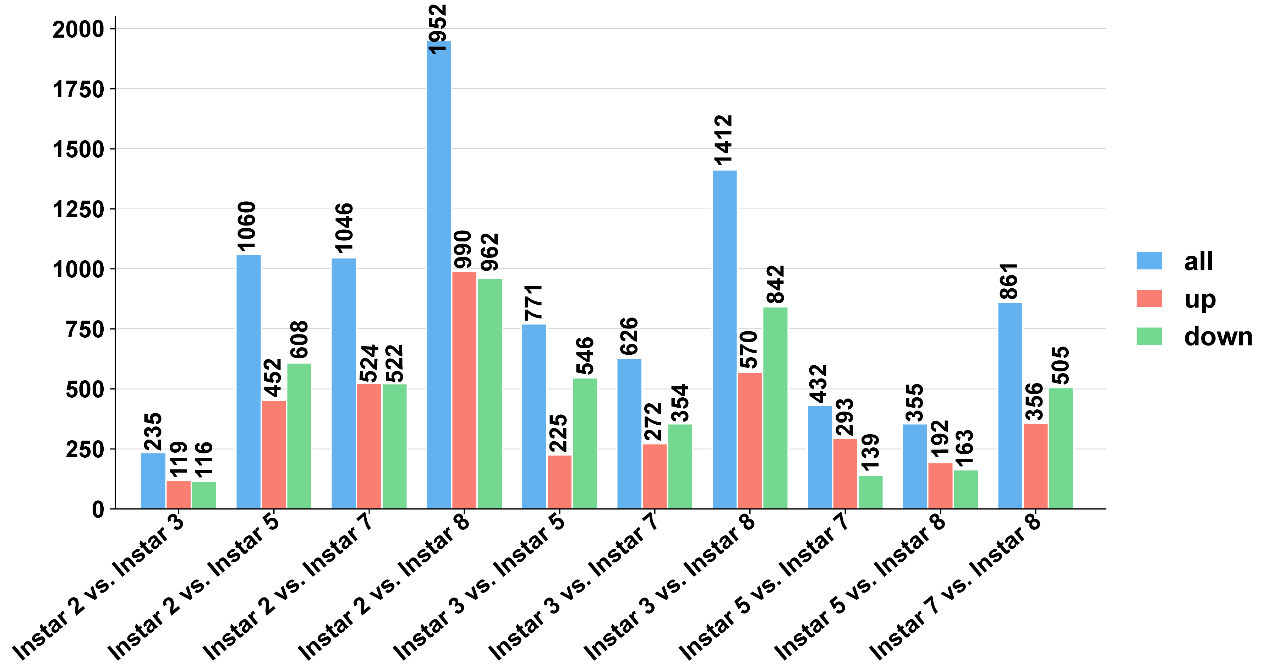
**

**Figure S4.** **The number of differentially expressed genes (DEGs)** **in the laterally leaf-like abdominal expansions of female individuals at the five different developmental stages.**

**
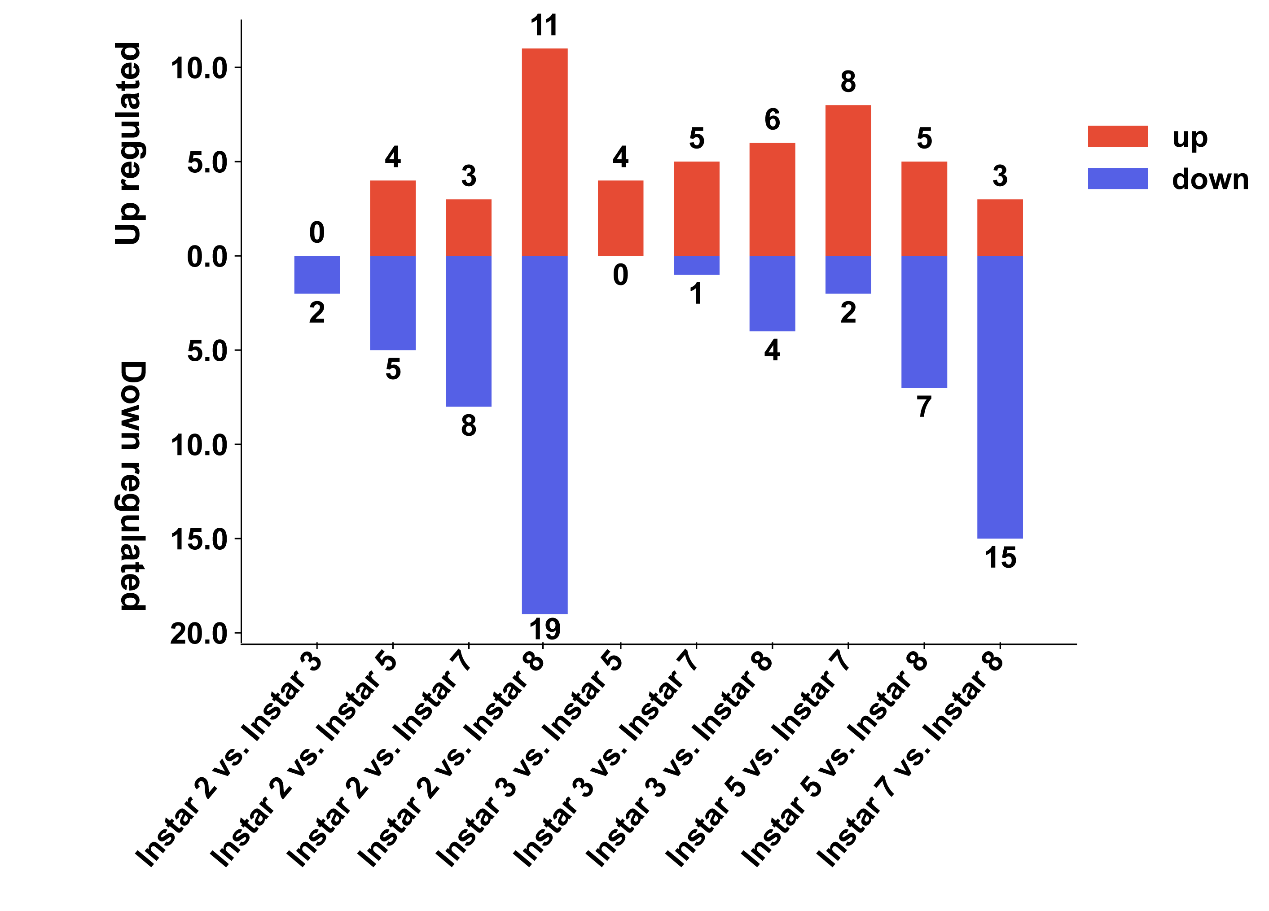
**

**Figure S5. The number of differentially expressed *Cuticle* genes in the laterally leaf-like abdominal expansions of female individuals at the five different developmental stages.**
