# Supplementary material for: Chromosome-level genome assembly and transcriptomes of the leaf insect Cryptophyllium westwoodii provide insights into the evolution of leaf-like masquerade
Source: Gigascience. 2026 Mar 2;15:giag022. doi: 10.1093/gigascience/giag022 (PMC13108252; doi:10.1093/gigascience/giag022)
Supplement: giag022_GIGA-D-25-00406_Revision_1 [file giag022_giga-d-25-00406_revision_1.pdf]

GigaScience

Chromosome-level genome assembly and transcriptomes of the leaf insect Cryptophyllium westwoodii provide insights into the evolution of leaf-like masquerade.

--Manuscript Draft--

**Manuscript Number:**	GIGA-D-25-00406R1
**Full Title:**	Chromosome-level genome assembly and transcriptomes of the leaf insect Cryptophyllium westwoodii provide insights into the evolution of leaf-like masquerade.
**Article Type:**	Research
**Funding Information:**	Yunnan Provincial Science and Technology Department (202401BC070017) — Dr. Xueyan Li<br>Yunnan Provincial Science and Technology Department (202105AC160039) — Dr. Xueyan Li<br>West Light Foundation, Chinese Academy of Sciences — Dr. Xueyan Li
**Abstract:**	Background: Leaf insects in the family Phylliidae are regarded as nature's ultimate masqueraders, evolving the leaf-resembling morphology to avoid predation. However, the lack of a high-quality reference genome for the leaf insects has hindered the exploration of the genetic mechanisms of leaf-like masquerade in insects.<br>Results: We generated a chromosome-level genome assembly of Cryptophyllium westwoodii using Nanopore and Hi-C sequencing. 98.3% of the 4.12 Gb assembly (scaffold N50 = 256.9 Mb, 98.6% BUSCO completeness) was anchored onto 15 pseudo-chromosomes, including 13 autosomes, an X chromosome, and a putative B chromosome. Genome annotation predicted a total of 2.29 Gb repeat sequences and 19,131 protein-coding genes. The chromosomal collinearity analysis indicated that many homologous gene fragments were detected between B-chromosome and other 14 A-chromosomes in C. westwoodii, suggesting B-chromosome could have a mosaic origin based on homologous gene fragments from A-chromosome. Comparative genomic and transcriptomic analyses indicated that resilin gene with 24 copies expanded in C. westwoodii, of which 10 copies showed significantly different expression in the laterally leaf-like abdominal expansions at five developmental stages. These findings suggest that Cuticle genes, particularly resilin, may contribute to the leaf masquerade morphology in C. westwoodii, implying their possible role in the evolution of this adaptive trait.<br>Conclusions: This study not only provides the first chromosome-level reference genome of leaf insects in Phylliidae, but also offers new insights into the leaf-like masquerade in leaf insects.<br>Keywords: Phylliidae, Cryptophyllium westwoodii, high quality genome, comparative genomics, Cuticle genes, leaf-resembling morphology.
**Corresponding Author:**	Xueyan Li, Ph.D<br>KIZ CAS: Kunming Institute of Zoology Chinese Academy of Sciences<br>Kunming, CHINA
**Corresponding Author Secondary Information:**	
**Corresponding Author's Institution:**	KIZ CAS: Kunming Institute of Zoology Chinese Academy of Sciences
**Corresponding Author's Secondary Institution:**	
**First Author:**	Chuyang Mao
**First Author Secondary Information:**	
**Order of Authors:**	Chuyang Mao<br>Zhiwei Dong

Powered by Editorial Manager® and ProduXion Manager® from Aries Systems Corporation

	Zihe Li
	Botong Zhou
	Yi Hu
	Jun Li
	Guichun Liu
	Zheng Zhou
	Jinwu He
	Yuhan Wu
	Wenting Wan
	Haoran Gao
	Wenhui Nie
	Ruoping Zhao
	Wen Wang
	Xueyan Li, Ph.D
**Order of Authors Secondary Information:**	
**Response to Reviewers:**	Dear Prof. Fan,<br>We are submitting our revised manuscript entitled “Chromosome-level genome assembly and transcriptomes of the leaf insect Cryptophyllium westwoodii provide insights into the evolution of leaf-like masquerade” (Manuscript ID: GIGA-D-25-00406). We appreciate all the positive comments and valuable suggestions from you and reviewers.<br><br>Based on reviewers’ comments and editor’s instructions, we have endeavored to improve our manuscript mainly as follows. (1) We have added a discussion comparing the gene content of the leaf insect B-chromosome to its counterparts in other insects and animals as suggested by reviewer#1. (2) We have extended of the methods used for on the functional enrichment analysis and added a bit more detail about the background gene set and the Benjamini-Hochberg false discovery rate (FDR) correction to the p-value as suggested by reviewer#1. (3) We have tried to incorporate three additional species (Medauroidea extradentata, Clitarchus hookeri, and Bacillus rossius redtenbacheri) into phylogenetic analyses as suggested by reviewer#2. Our result show that the addition of these species don’t affect the topology of tree. To maintain the focus on the core functional genomics objectives, we retained the original in-depth comparative framework centered on Timema monikensis, Dryococelus australis, and C. westwoodii. And we only discussed above species in the Discussion section. (4) We have modified keywords based on the comment of reviewer#2. Additionally, we checked the whole manuscript carefully, corrected typos and improved details of the language and writing. All the changes were highlighted in yellow in the revised version.<br><br>We provide detailed point-by-point responses to the reviewers’ comments as follows.<br><br>Thank you very much for your consideration.<br><br>Xueyan Li, Ph.D<br>State Key Laboratory of Genetic Evolution & Animal Models, Kunming Institute of Zoology, Chinese Academy of Sciences (CAS), Kunming, Yunnan 650223, China<br>Email: lixy@mail.kiz.ac.cn, Tel: 86-871-68125339, Fax: 86-871-68125338<br><br>Wen Wang, Ph.D<br>New Cornerstone Investigator<br>New Cornerstone Science Laboratory<br>School of Ecology and Environment, Northwestern Polytechnical University, Xi’an 710072, China<br>Adjunct Professor of State Key Laboratory of Genetic Evolution & Animal Models, KIZ,

Powered by Editorial Manager® and ProduXion Manager® from Aries Systems Corporation

CAS, Kunming 650223, China
Email: wenwang@nwpu.edu.cn; wwang@mail.kiz.ac.cn

Point-by-Point Responses
Reviewer #1: Dear Dr. Thomas Buckley
The authors have produced a high quality genome assembly. This is a welcome addition to insect genomics. Lead insects are a very interesting taxon and this genome will facilitate a lot of biology.
The finding of the B-chromosome is particularly interesting. This has been known in stick insects previously, but its gene content has not been examined. This is a novel report in that regard.

The analyses are sound, but I have a few questions about functional enrichment and testing.
R: We sincerely thank you for the positive comments on this work. Based on the reviewer's suggestion, we improved this manuscript as shown in the following.

The finding of the cuticle gene expansion and differential expression is also very interesting. The data at this point suggests a connection between this gene family and the leaf insect form, but this is only a correlation. And there are very few stick insect genomes available. I recommend toning down the statement in the abstract and making it clear this is still a hypothesis.
R: Thank you very much for your suggestion. As suggested, we have revised the statement in the abstract to make it more reasonable: "These findings suggest that cuticle genes, particularly resilin, may contribute to the leaf masquerade morphology in C. westwoodii, implying their possible role in the evolution of this adaptive trait" (Page 2, Lines 13-15).

Finally, I recommend referring to the order as "Phasmatodea" as this is what most researchers use nowadays (e.g., Catalogue of Life). Phasmida is used in older texts, a couple of web sites and a small number of authors.
R: Thank you very much. We have corrected the order name to "Phasmatodea" in the revised manuscript, except for the cited literature

Line 16-18. This sentence needs rewriting for clarity.
R: Thank you very much for the comment. In revised manuscript, we have rewritten this sentence to make it clear: "Very interestingly, among approximately 3500 species of Phasmatodea (representing 21 families), the remarkable adaptation of simulating leaves is almost exclusively limited to the family Phylliidae, which contains just over 100 species, whereas the majority of stick insects mimic branches [9, 12-14]" (Page 3, Lines 16-19).

Line 21. Phyllidae should start with "P" as it is a proper noun.
R: Thank you very much for pointing this. We have corrected it as "Phyllidae" in revised manuscript: "Cryptophyllium westwoodii (Fig. 1) is one of the representative species of the Phylliidae and disguises a nearly impeccable leaf masquerade with a leaf-like venation pattern and lobe-like extensions on the abdomen and legs [15, 16]" (Page 3, Line 22; Page 4, Lines 1-2).

Page 4, line 9. "Combined"
R: Thank you for pointing this. We have corrected it as "Combined" in revised manuscript:. "Combined with karyotyping, we assembled 13 autosomes, one X sex-chromosome and one candidate B-chromosome of this species." (Page 4, Lines 11-12).

Page 4, line 20. "….which is erected based on eggs…" Not sure of meaning here.
R: Thank you for these comments. In revised manuscript, we have rewritten this sentence to make it clear: "C. westwoodii is from a population reared in Kunming, Yunnan, China with Rubus sp. as host plant in our lab, and this population was established from eggs originally collected from Muang Fuang, Nang Ha, Laos in 2017 [15]." (Page 4, Lines 21-22; Page 5, Line 1).

Page 4, line 21. Individual rather than "larva".
R: Thank you for the comment. We have corrected it as "individual" in revised

manuscript: “One individual of the first-instar was collected for sequencing on the Illumina platform for genome survey and correct errors.” (Page5, Lines 1-2).

Page 4, line 22. "…sequencing on the Illumina platform…"
R: Thank you very much. We have corrected it as suggested: “One individual of the first-instar was collected for sequencing on the Illumina platform for genome survey and correct errors.” (Page5, Lines 1-3).

Page 6, line 3. No need to refer to this technology as "next-generation".
R: Thank you very much. We modified it as suggested: “For Illumina sequencing, genomic DNA was isolated from whole body of one larva individual using TreliefTM Animal Genomic DNA Kit (TsingKe, China).” (Page 6, Line 6).

Page 6, line 13. Refer to this as "Oxford Nanopore long read sequencing". This is the underlying technology, where as PromethION is the machine.
R: Thank you for the comment. We modified it as suggested: “For Oxford Nanopore long read sequencing, genomic DNA from another male adult was isolated to construct long DNA fragment libraries (NextOmics, China).” (Page 6, Line 16).

It probably would have been better to use tissue from the same individual as was sequenced with ONT for the polishing.
R: Thank you for your insightful and constructive comment regarding the use of polishing data. We fully agree with you that the optimal strategy for polishing a de novo genome assembly is to use short-read data from the exact same individual as the long-read (ONT) data. In our study, an individual was initially sequenced using short-read technology to perform genome survey. But unfortunately, the DNA yield from this individual was not enough for subsequent Oxford Nanopore long read sequencing. Thus, we collected another individual from the same batch with a highly similar genetic background for Oxford Nanopore long read sequencing.
To ensure the reliability and accuracy of our final assembly, we performed rigorous, multi-faceted quality assessments. The results (Table 1) indicate the assembly is high-quality (completeness, High contiguity, and consistency) and reliable for subsequent analyses. :
•Completeness: The final assembly shows high BUSCO completeness with 98.27%, indicating comprehensive gene space representation.
•High Contiguity: The assembly has a fragment N50 length of 256.76 Mb, demonstrating excellent continuity and a significant reduction in fragmentation.
•Consistency: The Illumina reads used for polishing mapped back to our final assembly with high mapping rates (94.75%), confirming the internal consistency of the consensus sequence.
In summary, based on the above evaluation of genome quality, even though our short-read and the long-read (ONT) data were not derived from the same individual, the impact on the genome assembly results is minimal. We appreciate the reviewer for understanding this.
Table 1: The statistics of genome assembly and annotation in Cryptophyllium westwoodii genome.

Page 7, line. What is meant exactly by "abdominal tissues". This is a very large part of the body with many tissue types. Be specific about exactly what was sampled. This is important for interpreting the gene expression results and for replication.
R: We sincerely thank the reviewer for raising this important point regarding the specificity of our tissue sampling. We agree that precise anatomical description is crucial for the interpretation of gene expression data. In this study, we specifically dissected and sequenced the highly modified part of abdomen, i.e. the laterally leaf-like abdominal expansions, which are the key morphological innovation underlying the leaf masquerade in this species. We have modified the expression into “the laterally leaf-like abdominal expansions”, instead of “abdominal tissues” in the main text, the legends of pictures and charts in the revised manuscript.

Page 9, line 9. "JUICEBOX" not "JUICERBox".
R: Thank you for pointing this. We have corrected it in revised manuscript: “Finally, contig orientation was corrected and the suspicious fragments were moved into unanchored groups by visual exploration of Hi-C heatmaps manually with JUICEBOX (version 1.11.08) [35].” (Page 9, Lines 10-12).

Powered by Editorial Manager® and ProduXion Manager® from Aries Systems Corporation

Page 12, line 22. Give a bit more detail on the functional enrichment analysis. What was the background gene set used for the enrichment test?
R: Thank you for these comments. We would like to clarify that we did perform FDR correction in our original analysis and added a bit more detail about the background gene set and the Benjamini-Hochberg false discovery rate (FDR) correction to the p-value: “GO enrichment analysis was performed using annotated genes on the B chromosome, and the background gene set for this analysis was the complete set of annotated genes in C. westwoodii. Significantly enriched GO terms (FDR-adjusted p-value ≤ 0.05, Benjamini-Hochberg method) were retained for further interpretation.” (Page 13, Lines 2-6).

Page 15, line 1. I think you need a false discovery rate correction here. Also, in this section what was the background gene set for functional enrichment tests?
R: Thank you for these comments. We would like to clarify that we did perform FDR correction in our original analysis and added a bit more detail about the background gene set and the Benjamini-Hochberg false discovery rate (FDR) correction to the p-value: “KEGG and GO enrichment analyses were analyzed by DAVID (RRID:SCR_001881) (version 6.8) [80] and KOBAS (RRID:SCR_006350) (version 3.0) [81] for expansion and contraction of gene families, positive selection genes, and rapidly evolving genes. For these enrichment analyses, the entire annotated genes of C. westwoodii served as the background gene set. Significant enriched GO and KEGG terms (FDR-adjusted p-value ≤ 0.05, Benjamini-Hochberg method) were retained.” (Page15, Lines 11-17).

Page 20, line 8. Diverged from Drycocelus lineage?
R: We thank the reviewer for this question and apologize for the lack of clarity in our original manuscript. We have improved it as “the ancestors of C. westwoodii and D. australis diverged ~49.67 million years ago (Mya) (Fig. 4).” (Page 20, Lines 15-16).

Page 21, lines 10-12. This is a conclusion and needs to be moved to "Discussion" section. Nevertheless, I'm not convinced you can ascribe these gene family expansions to the leaf insect body form as there are so many biological differences between leaf and stick insects. Furthermore, you are only comparing one leaf insect and one stick insect genome. I think you can propose this as a hypothesis that needs testing with more data.
R: Thank you much for these comments. We agree with all points raised and have revised the manuscript accordingly to better reflect the preliminary nature of this finding and to speculate that it is related to the development of the cuticle and epidermis. As suggested, we moved the sentence into "Discussion" section and improved them as the followings: “Interestingly, our comparative genomics demonstrated expanded and contracted gene families were functionally enriched in structural constituent of cuticle and skin development, which may affect the development of the cuticle and epidermis in C. westwoodii.” (Page 27, Lines 14-17).

Page 22, line 22. This is a conclusion and needs to move to the Discussion section. Also,
R: We thank the reviewer for these comments. We agree that the original phrasing of line 22 was overly conclusive for the Results section. We have opted to rephrase the sentence to better reflect a summary of our findings at this stage in result Part as the following: “The findings highlight a central role for Cuticle genes in the abdomen tissue of female individuals at the five developmental stages at both genome level and expression level.” (Page 23, Lines 5-8). At the same time, we also made a conclusion in the discussion section of original manuscript: “These findings suggest that Cuticle genes may play important roles in the evolution of leaf-like abdominal extension at both genome-level and expression-level.” (Page 27, Lines 20-22).
We thank the reviewer for this suggestion (line 15-18). We fully agree with the principle of moving explanatory and detailed explanations to the "Discussion" section. Based on your feedback, we have reorganized lines 15-18 and related paragraphs to ensure the purity of the “Results” section: “Notably, cluster C4 (369 genes) related to structural constituent of chitin-based cuticle and chitin-based cuticle development, exhibited a significant expression peak specifically during the 3rd instar (Fig. 6C; Supplementary Table S24).” (Page 23, Lines 1-3). In the "Discussion" section, we have discussed this part of the content: “Among ten differentially expressed copies of resilin, five copies

Powered by Editorial Manager® and ProduXion Manager® from Aries Systems Corporation

were divided into cluster 4 (C4) where the 3rd instar can be an important time node for cuticle genes. At this stage from the 2nd instar to the 3rd instar, two copies of resilin were significantly differentially upregulation while the abdominal extensions presented obviously enlargement a lot and leaf-like body initially formed." (Page 28, Lines 13-17).

Pages 25-26. The discussion on the B chromosome is interesting. How does the gene content of the leaf insect B-chromosome differ from other insects (if known) or animals?
R: Thank you very much for your positive feedback and suggestive comments. Based on your comment, we have analyzed the functions of genes on B-chromosome in different animals, which is summarized in an additional table (Supplementary Table S19). The analysis show that that the gene content of the B chromosome in the leaf-stick insect exhibits dual characteristics: on the one hand, it is conserved in core functions compared to other animals, and on the other hand, it carries unique genes with disparate functions. We now have added the related description in Discussion section as the following: "In the B chromosomes of two locusts Abracris flavolineata [100] and Eyprepocnemis plorans [101], genes directly involved in chromosome formation and cell cycle related functions have also been identified. Thess functional genes are not limited to insects; they has also been also found in the mammals red fox and Chinese raccoon dog [102], lizard Anolis carolinensis [103] and fishes including Astyanax mexicanus, Astyanax correntinus and Astatotilapia latifasciata [100, 104, 105]." (Page 25, Lines 15-21) and " In this study, we found some genes that have not been reported on the B chromosome, such as glycoside hydrolase family 28 (the plant cell wall digestion enzymes) [106, 107], Gustatory receptor 26b (Gustatory receptor) [108, 109] and cytochrome P450 6j1 (disintoxication) [89] (Supplementary Table S19). This finding suggests that the majority of genes on B-chromosomes across different species are functionally enriched for cell cycle and chromosome condensation, but each specie also can harbor a unique subset of genes with disparate functions" (Page 26, Lines 5-11) and added an additional table (Supplementary Table S19) to analyze the functions of genes on B-chromosome.

Reviewer #2:
Here, the authors generated and now present the first genome of a leaf insect (Phasmatodea: Phylliidae), which is indeed a worthwhile endeavor and provides valuable genetic resources for numerous future studies on the development and evolution of these charismatic creatures. While publication of this kind of results needs to be descriptive to a certain point, the authors do their best to provide intriguing information when presenting their results, among them the assumption that the cuticle gene resilin plays a crucial role in evolution of leaf masquerade. The methodical approaches all appear to be state-of-the-art with necessary and nice figures provided. There are a few shortcomings nevertheless. Besides minor typos etc. (phasmida must be Phasmida; E. messelensis must be E. messelense; keywords must NOT repeat words from the title!), there are also a few major aspects that need to be addressed.
R: We sincerely thank the reviewer for the positive comments and constructive criticisms. We have improved the manuscript based on your invaluable suggestions as shown in the following.
We have corrected "phasmida" into the proper order name "Phasmatodea", "E. messelensis" into "E. messelense" in the whole manuscript: "In addition, the first fossil leaf insect (E. messelense) [119] presented extensions on the abdominal terga and sterna as in modern leaf insects [120]" (Page 27, Lines 7-9).
We have also revised the list of keywords to ensure no repeat words from the title. The new keywords in revised version are "Phylliidae, Cryptophyllium westwoodii, high quality genome, comparative genomics, Cuticle genes, leaf-resembling morphology" (Page 2, Lines 19-20).

Lines 7-9: "The assembled genome size of C. westwoodii is 4.09 Gb, which is the largest one in the published genomes of the order Phasmida …"
This statement is not correct: Clitarchus hookeri, a stick insect from New Zealand, has a larger genome with 4.2 Gb published by Wu et al. (BMC Genomics (2017) 18:884 (DOI 10.1186/s12864-017-4245-x "Assembling large genomes: analysis of the stick insect (Clitarchus hookeri) genome reveals a high repeat content and sexbiased genes associated with reproduction ") a paper, which has obviously been overlooked by the authors and needs to be thoroughly discussed and probably included in the analyses provided here.

	R: We thank the reviewer for the valuable comment. We carefully checked the reference Wu et al., 2017 (on Clitarchus hookeri), and cited it in our revised manuscript as reference [92] as shown in the following: "Compared with previously published genomes of walking sticks of the order Phasmatodea [63, 66, 91-94], the chromosome-level assembly of C. westwoodii represents the highest-quality assembly with the second highest N50 value (N50=256.87 Mb) and the lowest number of scaffolds (n=179) (Supplementary Table S4)." (Page 24, Lines 6-10).. It is noted that the initial survey assembly by Wu et al. (2017) estimated a size of 4.2 Gb for Clitarchus hookeri, but a chromosome-level genome assembly for C. hookeri later published by Choi et al (Molecular Ecology (2023):17422. DOI: 10.1111/mec.17422 "Multiple hybridization events and repeated evolution of homoeologue expression bias in parthenogenetic, polyploid New Zealand stick insects", which we had already cited in our original manuscript as reference [91]) has revised the genome size down to approximately 2.8 Gb. This refinement of Clitarchus hookeri genome resulted from improved genome assembly technologies which resolve complex repetitive regions more accurately. Therefore, our conclusion that the C. westwoodii genome (4.09 Gb) is the largest currently published in Phasmatodea is appropriate when referencing the latest and highest-quality assemblies. We have already compiled information on each published genome of walking sticks of the order Phasmatodea in the original manuscript, please refer to Supplementary Table S4.<br>Supplementary Table S4. The comparison of assembly metrics among Cryptophyllium westwoodii and other five stick insects of the order Phasmatodea. Statistical indicators include genome size, Scaffold N50 length and number, GC cnontent, Repeat content and BUSCO rate.<br><br>There is also another genome paper not mentioned/evaluated: Kraus et al. PLoS One. 2009 Sep 29;4(9): e7223. doi: 10.1371/journal.pone.0007223 "The Genome of the Stick Insect Medauroidea extradentata Is Strongly Methylated within Genes and Repetitive DNAs" but I am not sure how relevant this paper might be for the current study.<br>R: We sincerely thank the reviewer for the comment. We carefully read the paper of Kraus et al. (2009) on the methylome of Medauroidea extradentata, and appreciate the important contribution of this work to the genomic characterization of this species. Nevertheless, considering our current study focused on the genome assembly and annotation, rather than on DNA methylation patterns, we didn't cite this aricle, but we have cited the more recent genome assembly of M. extradentata by Brand et al. G3: Genes, Genomes, Genetics. 2018;8(5):1403–1408 "The draft genome of the invasive walking stick, Medauroidea extradendata, reveals extensive lineage-specific gene family expansions of cell wall degrading enzymes in phasmatodea" as reference [94] in our main text as follows: "Compared with previously published genomes of walking sticks of the order Phasmatodea [63, 66, 91-94], the chromosome-level assembly of C. westwoodii represents the highest-quality assembly with the second highest N50 value (N50=256.87 Mb) and the lowest number of scaffolds (n=179) (Supplementary Table S4)." in our initial manuscript (Page24, Line 6-10). This paper focus on genome structure and gene family evolution, which is more immediately relevant to the current study. To compare the quality of the genome we assembled, we have already compiled information on each published genome of walking sticks of the order Phasmatodea including M. extradentata in the original manuscript, please refer to Supplementary Table S4.<br>Supplementary Table S4. The comparison of assembly metrics among Cryptophyllium westwoodii and other five stick insects of the order Phasmatodea. Statistical indicators include genome size, Scaffold N50 length and number, GC cnontent, Repeat content and BUSCO rate.<br><br>Beyond those studies, I think that most relevant papers are referenced. Not so the leaf insect paper by Bank and colleagues ("A tree of leaves: Phylogeny and historical biogeography of the leaf insects (Phasmatodea: Phylliidae" Communications Biology 2021, doi.org/10.1038/s42003-021-02436-z) which should be consulted whether it bears further valuable information on these insects.<br>R: We thank the reviewer for the suggestion. In the revised version of our manuscript, we have now cited this paper in the Introduction section as reference [9]: "Very interestingly, among approximately 3500 species of Phasmatodea (representing 21 families), the remarkable adaptation of simulating leaves is almost exclusively limited to the family Phylliidae, which contains just over 100 species, whereas the majority of

	stick insects mimic branches [9, 12-14]" (Page 3, Lines 16-19) and "A more fascinating example of masquerade is from leaf and stick insects in the Order Phasmatodea, which mainly distribute in tropical regions and temperate regions [9-10]." (Page 3, Lines 12-13).<br><br>I summary, when the studies omitted only need to be discussed in the present manuscript, it can be published after minor revision. If the data from these studies need to be included in the analyses, a major revision is definitely needed. The phylogeny presented is nearly meaningless given the few taxa included and would benefit from inclusion of Clitarchus and Medauroidea for sure.<br>R: We sincerely thank you for providing all these constructive comments for our manuscipt. We have carefully considered all of your points and have revised the manuscript accordingly. Our point-by-point responses are detailed below. Regarding the omitted studies, we have already compiled information on each published genome of walking sticks of the order Phasmatodea including M. extradentata, C. hookeri and Bacillus rossius redtenbacheri in the original manuscript. These information are used to compare the quality of genome assembly in Discussion section: "Compared with previously published genomes of walking sticks of the order Phasmatodea[63, 66, 91-94], the chromosome-level assembly of C. westwoodii represents the highest-quality assembly with the second highest N50 value (N50=256.87 Mb) and the lowest number of scaffolds (n=179) (Supplementary Table S4). The assembled genome size of C. westwoodii is 4.09 Gb, which is the largest one in the published genomes of the order Phasmatodea (Supplementary Table S4)." (Page 24, Lines 9-15)."<br>Supplementary Table S4. The comparison of assembly metrics among Cryptophyllium westwoodii and other five stick insects of the order Phasmatodea. Statistical indicators include genome size, Scaffold N50 length and number, GC cnontent, Repeat content and BUSCO rate.<br><br>In order to check the phylogeny with inclusion of Clitarchus and Medauroidea, we added three species (M. extradentata, C. hookeri and B. rossius redtenbacheri) to perform phylogenetic analysis again. We reconstructed a phylogenomic tree of 11 species based on 841 single-copy orthologs. The phylogenetic relationships of 11 species were well recovered, with all the nodes being strongly supported (UFB/SH-aLRT = 100/100) (R_Figure_1), and show the same topology as that of Figure 4. Considering that inclusion of three species didn't effect the topology of tree, and that the primary objective of this study was not to discuss phylogenetic relationships, so we only discussed those omitted species in the Discussion section as mentioned above. We have retained our original, focused analytical framework for the core genomic comparisons. This approach allows us to maintain a clear and in-depth focus on the functional genomic questions that are the central thrust of our manuscript, without shifting its scope. We hope these revisions thoughtfully address the reviewer's point by acknowledging the limitation, providing the necessary context, and clarifying the intent of our analysis, all while preserving the focused narrative and conclusions of our study.<br>R_Figure_1. Phylogenetic relationship among the eleven species inferred by the amino acid sequences of the 258 single-copy genes.<br>Figure 4: Phylogenetic and evolutionary analyses of Cryptophyllium westwoodii genome. In the left panel, blue and red numbers on the branch show the number of expanded and contracted gene families for each clade. The black numbers are divergence times. In the right panel, the numbers of gene families (orthogroups) are shown as barplots. Orthogroups of different categories are in different colors.
**Additional Information:**	
**Question**	**Response**
Are you submitting this manuscript to a special series or article collection?	No
**Experimental design and statistics**<br><br>Full details of the experimental design and statistical methods used should be given in the Methods section, as detailed in our	Yes; Yes

Powered by Editorial Manager® and ProduXion Manager® from Aries Systems Corporation

Minimum Standards Reporting Checklist. Information essential to interpreting the data presented should be made available in the figure legends.<br><br>Have you included all the information requested in your manuscript?	
**Resources**<br><br>A description of all resources used, including antibodies, cell lines, animals and software tools, with enough information to allow them to be uniquely identified, should be included in the Methods section. Authors are strongly encouraged to cite Research Resource Identifiers (RRIDs) for antibodies, model organisms and tools, where possible.<br><br>Have you included the information requested as detailed in our Minimum Standards Reporting Checklist?	Yes; Yes
**Availability of data and materials**<br><br>All datasets and code on which the conclusions of the paper rely must be either included in your submission or deposited in publicly available repositories (where available and ethically appropriate), referencing such data using a unique identifier in the references and in the "Availability of Data and Materials" section of your manuscript.<br><br>Have you have met the above requirement as detailed in our Minimum Standards Reporting Checklist?	Yes; Yes
GigaScience has policies and guidelines in place for the use of generative AI-writing tools such as ChatGPT. If you have used such writing tools to assist with writing the manuscript this must be declared and cited in the text. Authors should not list AI-writing tools and other	No; No

AI-assisted technologies as an author or co-author and should acknowledge that they are fully responsible for text generated or refined by AI-writing tools.<p><br><br>A summary of use (particularly in the introduction or among methods) needs to be included at the end of the paper, and the outputs should also be included as a supplementary file hosted in GigaDB or other open repositories. Please read our guidelines for more information. <p><br><br>By submitting to GigaScience, you are aware of the journal's AI-writing tools policy, and if you have declared use of such tools below, you have acknowledged this where appropriate in your manuscript and have made a summary of use and outputs available. <p><br><b>AI-assisted writing tools have been used in the preparation of this manuscript?	

Powered by Editorial Manager® and ProduXion Manager® from Aries Systems Corporation

Manuscript-R1

Click here to access/download;Manuscript;GIGA-D-25-00406-R1.docx

Chromosome-level genome assembly and transcriptomes of the leaf insect *Cryptophyllium westwoodii* provide insights into the evolution of leaf-like masquerade

Chuyang Mao[1,3,†], Zhiwei Dong[1,†], Zihe Li[2,†], Botong Zhou[2], Yi Hu[1], Jun Li[1,3], Guichun Liu[1,5], Zheng Zhou[1,2], Jinwu He[1], Yuhan Wu[1,3], Wenting Wan[1], Haoran Gao[4], Ruoping Zhao[1], Wenhui Nie[1], Wen Wang[1,2,3,*], Xueyan Li[1,3,*]

[1] State Key Laboratory of Genetic Evolution & Animal Models, Kunming Institute of Zoology, Chinese Academy of Sciences, Kunming 650223, China

[2] New Cornerstone Science Laboratory, Shaanxi Key Laboratory of Qinling Ecological Intelligent Monitoring and Protection, School of Ecology and Environment, Northwestern Polytechnical University, Xi'an 710072, China

[3] Kunming College of Life Science, University of Chinese Academy of Sciences, Kunming 650223, China

[4] College of Plant Protection, Yunnan Agricultural University, Kunming 650223, China

[5] College of Medicine, Xi'an International University, Xi'an, 710077, China

[*]Correspondence address. lixy@mail.kiz.ac.cn (X.Y.L.); wenwang@nwpu.edu.cn (W.W.)

[†] These authors contributed equally.

Abstract

Background: Leaf insects in the family Phylliidae are regarded as nature's ultimate masqueraders, evolving the leaf-resembling morphology to avoid predation. However, the lack of a high-quality reference genome for the leaf insects has hindered the exploration of the genetic mechanisms of leaf-like masquerade in insects.

Results: We generated a chromosome-level genome assembly of *Cryptophyllium westwoodii* using Nanopore and Hi-C sequencing. 98.3% of the 4.12 Gb assembly (scaffold N50 = 256.9 Mb, 98.6% BUSCO completeness) was anchored onto 15 pseudo-chromosomes, including 13 autosomes, an X chromosome, and a putative B chromosome. Genome annotation predicted a total of 2.29 Gb repeat sequences and 19,131 protein-coding genes. The chromosomal collinearity analysis indicated that many homologous gene fragments were detected between B-chromosome and other 14 A-chromosomes in *C. westwoodii*, suggesting B-chromosome could have a mosaic origin based on homologous gene fragments from A-chromosome. Comparative genomic and transcriptomic analyses indicated that *resilin* gene with 24 copies expanded in *C. westwoodii*, of which 10 copies showed significantly different expression in the laterally leaf-like abdominal expansions at five developmental stages. These findings suggest that *Cuticle* genes, particularly *resilin*, may contribute to the leaf masquerade morphology in *C. westwoodii*, implying their possible role in the evolution of this adaptive trait.

Conclusions: This study not only provides the first chromosome-level reference genome of leaf insects in Phylliidae, but also offers new insights into the leaf-like masquerade in leaf insects.

Keywords: Phylliidae, *Cryptophyllium westwoodii*, high quality genome, comparative genomics, *Cuticle* genes, leaf-resembling morphology.

Introduction

Insecta, as the largest class in the animal kingdom, often employ strategies like crypsis, masquerade, and mimicry through morphological changes across species or developmental stages to evade predators or enhance hunting efficiency due to their position at the food chain's base [1-3]. Masquerade refers to the accurate imitation of the surrounding bark, leaves or flowers, which widely exists in various insect groups [4]. For examples, the body and limbs of the orchid mantis (*Hymenopus coronatus*) have evolved the structures and colors similar to orchid petals [5, 6], and the wings of the dead-leaf butterfly (*Kallima inachus*) show the shape, veins, and color of withered leaves [7, 8].

A more fascinating example of masquerade is from leaf and stick insects in the Order Phasmatodea, which mainly distribute in tropical regions and temperate regions [9, 10]. Phasmatodea species can match the shape of the plant with their own body shape, dress up as the imitated plant, or branch or leaf, making it difficult to detect their existence [11]. Very interestingly, among approximately 3500 species of Phasmatodea (representing 21 families), the remarkable adaptation of simulating leaves is almost exclusively limited to the family Phylliidae, which contains just over 100 species, whereas the majority of stick insects mimic branches [9, 12-14]. In 1889, Wallace reported on the phenomenon of leaf masquerade in insects, stating that "leaf insects (Phylliidae) can be considered the most perfect masquerade in the insect class" [3]. *Cryptophyllium westwoodii* (**Fig. 1**) is one of the representative species of the Phylliidae

and disguises a nearly impeccable leaf masquerade with a leaf-like venation pattern and lobe-like extensions on the abdomen and legs [15, 16]. However, although the ecological and adaptive evolutionary significance of leaf-like masquerade is well-known, little is known about the genetic basis of the origin and evolution of this complex phenotypic trait. Among them, in particular, the lack of reference genomes for representative species of leaf insects limits the exploration of the genetic mechanisms underlying this phenomenon.

In order to investigate the genomic basis of leaf morphology in leaf insects, we selected *C. westwoodii* (**Fig. 1**) as one representative species of Phylliidae to assemble its chromosome-level reference genome using Nanopore sequencing and Hi-C. Combined with karyotyping, we assembled 13 autosomes, one X sex-chromosome and one candidate B-chromosome of this species. Combining comparative genomic and developmental transcriptome analyses, we found that some *cuticle* genes especially *resilin* may play important roles in abdominal leaf-like development. In summary, the findings provide important genomic resource for investigating the evolution leaf-like masquerade, and also offer new insights into the leaf-like masquerade in leaf insects.

Methods

Insects

C. westwoodii is from a population reared in Kunming, Yunnan, China with *Rubus* sp. as host plant in our lab, and this population was established from eggs originally

collected from Muang Fuang, Nang Ha, Laos in 2017 [15]. One individual of the first-instar was collected for sequencing on the Illumina platform for genome survey and correct errors. One male adult was sequenced on the Nanopore platform and another male adult was collected for Hi-C sequencing for *de novo* chromosome-level genome assembly. Females and males were collected for karyotype analysis. The whole body of one female adult and one male adult, and the laterally leaf-like abdominal expansions of three female individuals at five developmental stages were collected for transcriptomic sequencing.

Karyotype analysis

Sexually mature individuals (both female and male) from *C. westwoodii* were used for the karyotype analysis referring to previously described method [17, 18] with some modifications. Firstly, inject colchicine (0.1%) into the abdominal cavity of both female and male insects for one hour. Gonads were dissected with shaving off the surrounding connective tissue from these female and male insects in 1 × PBS, subjected to hypotonic treatment in 0.05% sodium citrate for 10 min, and then transferred to Carnoy's solution (a mixture solution of methanol: acetic acid 3:1) for fixation two times, each lasting 25 minutes. After aspirating the Carnot solution, the fixed material was treated with a 60% solution of glacial acetic acid and then blown with the tip of a pipette to evenly distribute the tissue. The approximately 20–40 μl turbid liquid were taken to quickly drop onto a glass slide which was -4 °C frozen storage. The slides were dried in an oven at 42 °C and then stained with 10% Giemsa solution for one hour. After dumping the

dye solution, the slides were washed by ddH_2O several times and air dried for 30 min. The slides were observed under a 630× light microscope (ZEISS, Axio Imager.D2) and the metaphase cells with well-dispersed chromosomes were selected for photography.

Genome sequencing and survey

For Illumina sequencing, genomic DNA was isolated from whole body of one larva individual using TreliefTM Animal Genomic DNA Kit (TsingKe, China). Paired-end libraries with 350 bp insert size were generated using NEB Next® Ultra DNA Library Prep Kit for Illumina HiSeq4000 platform at Novogene (Tianjin, China). The raw reads containing > 90% bases with a quality <Q20 or more than 10% of Ns were filtered using Fastp (RRID:SCR_016962) (version 0.20.1) [19] and duplicates in paired reads were filtered using FastUniq (RRID:SCR_000682) (version 1.1) [20]. The rest clean reads were used to estimate genome size based on the 17 k-mer size using kmerfreq (version 1.0) [21] and were also used to correct errors of de novo assembled genomes at the base level.

For Oxford Nanopore long read sequencing, genomic DNA from another male adult was isolated to construct long DNA fragment libraries (NextOmics, China). Long DNA fragments were selected using the BluePippin system (Sage Science, USA) and then attached to sequencing adapters using a Ligation Sequencing Kit (Oxford Nanopore, catalog number: SQK-LSK109). The quantified library fragments were then sequenced on a Nanopore PromethION sequencer (Oxford Nanopore Technologies, UK) instrument at the Genome Center of Nextomics (Wuhan, China).

The sample treatment and the library construction for Hi-C sequencing followed the previously described protocol [22, 23]. The tissue samples from the whole body of a male adult were fixed, lysed, separated, and digested with restriction enzyme MboI overnight. The Hi-C libraries with fragments of 200–300 bp were constructed by Covaris M220 (Covaris, Woburn, MA) and Dynabeads® MyOneTM Streptavidin C1 (Thermofisher) and sequenced on the Illumina NovaSeq sequencing platform at Novogene (Tianjin, China).

Transcriptome sequencing and transcriptomic analysis

For transcriptome sequencing, we collected the whole body of one female adult and one male adult, the laterally leaf-like abdominal expansions of three female individuals at five developmental stages including second-, third-, fifth-, and seventh- instar larvae and eighth- instar (adults). We extracted total RNA using the TRIzol reagent (Thermo Fisher Scientific, USA). Paired-end libraries were constructed using the VAHTS RNA-seq V8 Library Prep Kit (Vazyme, Nanjing, China). The libraries were then sequenced on the Illumina NovaSeq 6000 platform with PE reads of 150 bp. Trimmomatic (RRID:SCR_011848) (version 0.36) [24] was used to remove adaptor sequences and filter low-quality reads from raw reads, with the following parameters: LEADING:3 TRAILING:3 SLIDINGWINDOW:4:15 MINLEN:40. The RNA-seq clean reads was were aligned to the *C. westwoodii* genome by Hisat2 (RRID:SCR_015530) (version 2.2.1) [25] and then assembled using StringTie (RRID:SCR_016323) (version 2.1.7)

[26]. The gene expression levels (Transcript per million; TPM) were quantified by StringTie (RRID:SCR_016323) (version 2.1.7) [26] based on the corresponding transcript annotation and number of reads mapping to genes fragments. To cluster expression profiles over five developmental stages, all the genes were divided into the different clusters by analyzing the average normalized TPM of genes within the Mfuzz sofware (RRID:SCR_000523) (version 2.48.0) [27] and the GO enrichment analysis was performed on the genes in each cluster. The express trend line chart, the heatmap and GO enrichment information were drawn using the ClusterGVis package [28].

Genes differentially expressed in the laterally leaf-like abdominal expansions of female individuals at the five developmental stages were respectively identified as follows. Non-normalized read counts for all detected genes were acquired by StringTie (RRID:SCR_016323) (version 2.1.7) [26] and a reads count table was generated by the Python script “prepDE.py” in the StringTie package. Then the differentially expressed genes (DEGs) were identified by DESeq2 (RRID:SCR_015687) (version 1.20.0) [29] based on negative binomial generalized linear models. Finally, the DEGs in different groups were retained with a $|\log2(\text{fold change})| > 2$ and adjusted p value < 0.05 (using the Benjamini-Hochberg algorithm).

Genome assembly and chromosome construction

The draft contig assembly was generated using nextDenovo (RRID:SCR_025033) (version 2.5.0) [30] with Nanopore reads. To reduce redundancy, haplotigs and overlapping contigs were further removed based on read depth using purge_dups (RRID:SCR_021173) (version 1.2.3) [31]. Next, both the Illumina data and Nanopore reads were further used to polish the assembly using nextPolish (RRID:SCR_025232) (version 1.3.1) [32]. Then, the Hi-C paired-end reads were mapped to the polished assembly iteratively and the paired tags were filtered using a restriction enzyme digesting fragments by JUICER (RRID:SCR_017226) (version 1.6) [33]. 3D-DNA software (RRID:SCR_017227) (version 180922) [34] was used to order and assign contig orientations and generated scaffolds. Finally, contig orientation was corrected and the suspicious fragments were moved into unanchored groups by visual exploration of Hi-C heatmaps manually with JUICEBOX (version 1.11.08) [35].

Quality assessment of genome assembly

The following three methods are used to evaluate the quality of the assembled genome. Firstly, the Illumina reads and Nanopore reads were mapped to the chromosome-level genome assembly using BWA-men (RRID:SCR_010910) (version 0.7.11) [36] and minimap2 (RRID:SCR_018550) (version 5.1) [37]; then the mapping ratio was calculated by SAMTOOLS (RRID:SCR_002105) (version 1.3.1) [38]. Secondly, the assembly indicators such as genome size, Scaffolds number, N50, GC content, and repeat content of the genome were calculated by the homemade scripts. Finally, BUSCO (RRID:SCR_015008) (version 5.2.2) [39] was conducted to assess the genome

completeness based on the Insecta odb10 BUSCO set [40].

Repeat and protein-coding gene prediction

For repeat annotation, we used LTR_FINDER (RRID:SCR_015247) (version 1.05) [41] to identify long termimal repeats retrotransposons and used TRF (RRID:SCR_022193) (version 4.09) [42] software to identify tandem repeats. Next, RepeatMasker (RRID:SCR_012954) (version 4.0.5) [43] was used to find ransposable elements (TEs) by mapping sequences against Repbase TE library at the DNA level and RepeatProteinMask (RRID:SCR_012954) (version 4.0.6) [44] was used to identify TE-relevant proteins at the protein level. Subsequently, we used RepeatMasker (RRID:SCR_012954) (version 4.0.5) [43] through de novo repeat library build by RepeatModeler (RRID:SCR_015027) (version 1.0.4) [45] to de novo predict transposable elements (TEs).

We adopted a combination of homologous prediction, de novo prediction, and transcriptome prediction methods to annotate protein-coding genes. For homology-based predictions, the protein sequences of three termites *(Zootermopsis nevadensis* [GCA_000696155.1] [46], *Cryptotermes secundus* [GCA_002891405.2] [47], *Reticulitermes speratus* [GCA_021605165.1] [48]), one locust (*Schistocerca nitens* [GCA_023898315.2]), two model insects (*Drosophila melanogaster* [GCA_000001215.4] [49], *Tribolium castaneum* [GCA_000002335.3] [50]) from NCBI were aligned to the *C. westwoodii* genome using tblastn (RRID:SCR_011822) (version 2.2.26) [51] with an E-value cutoff of 1e-5 and the obtained BLAST hits were

merged using Solar (RRID:SCR_000850) (version 0.9.6) [52] software. GeneWise (RRID:SCR_015054) (version2.2.0) [53] was used to predicts the complete gene structure based on the corresponding gene regions of each BLAST hit. For transcriptome prediction, the transcriptome of *C. westwoodii* was aligned using Hisat2 (RRID:SCR_015530) (version 2.2.1) [25] and then assembled using StringTie (RRID:SCR_016323) (version 2.1.7) [26]. The assembled transcriptome sequence was mapped to the genome for gene structural prediction using Transdecoder (RRID:SCR_017647) (version 5.5.0) [54] and PASA (RRID:SCR_014656) (version 2.3.3) [55]. For *de novo* prediction, the protein-coding gene sets of *C. secundus* and the assembled transcripts of *C. westwoodii* were used to train ab initio predicting models by Augustus (RRID:SCR_008417) (version 3.4.0) [56]. Based on *C. secundus, D. melanogaster* and transcripts ab initio model, *de novo* prediction was finished by Augustus (RRID:SCR_008417) (version 3.4.0) [56]. A total of 10 *de novo*, homology and transcriptome gene sets were merged to form a comprehensive and non-redundant gene set using EvidenceModeler (RRID:SCR_014659) (version 1.1.1) [57].

Gene function annotation

The predicted protein-coding sequences were to aligned to four databases (TrEMBL (RRID:SCR_002380) [58], SwissProt (RRID:SCR_021164) [59], KEGG (RRID:SCR_012773) [60] and NR [61]) with an E-value cutoff of 1e-5 to obtain functional information using BLASTP (RRID:SCR_001010) (version 2.2.26) [51]. Interproscan (RRID:SCR_005829) (version 5.8.0) [62] software was used to search for

known motifs and domains by mapping protein-coding sequences to Pfam (RRID:SCR_004726) (version27.0), PRINTS (RRID:SCR_003412) (version 42.0), ProDom (RRID:SCR_006969) (version 2006.1) and SMART (RRID:SCR_005026) (version 6.2) databases.

B-chromosome structure and function analysis

To investigate the origin of the B-chromosome in the *C. westwoodii,* The collinearity relationship between two species (*C. westwoodii* and *Dryococelus australis* [GCA_029891345.1] [63]) and within *C. westwoodii* (B-chromosome and other 14 A-chromosomes) was established. According to the genome annotation file of *C. westwoodii* and *D. australis*, the protein sequences of all 15 chromosomes of the *C. westwoodii* were aligned to the protein sequences of 17 chromosomes of the *D. australis* and the protein sequences of the B-chromosome in the *C. westwoodii* were aligned to the protein sequences of other 14 A-chromosomes in the *C. westwoodii* by BLASTP (RRID:SCR_001010) (version 2.2.26) [51]. MCScanX (RRID:SCR_022067) (version 2.2.26) [64] software was searched for collinear blocks based on alignment information and annotated GFF files. The colinearity between two species (*C. westwoodii* and *D. australis*) and within *C. westwoodii* (B-chromosome and other 14-A chromosomes) was displayed by circos (RRID:SCR_011798) (version 0.69-6) [65].

To compare the differences in structure and function between the B-chromosome and other 14 A-chromosomes, each chromosome sequence depth was plotted with a window of 500 bp by the bam file obtained by aligning the Nanopore reads to the

assembly genome of *C. westwoodii* and the proportion of repetitive sequence types for each chromosome was calculated. GO enrichment analysis was performed using annotated genes on the B chromosome, and the background gene set for this analysis was the complete set of annotated genes in *C. westwoodii*. Significantly enriched GO terms (FDR-adjusted *p*-value $\leqslant$ 0.05, Benjamini-Hochberg method) were retained for further interpretation.

Identification of orthologous groups and phylogenetic analysis

To cluster families of protein-coding genes, we extracted protein sequences from *C. westwoodii* and other seven insects including two stick insects (*Timema monikensis* [66], *D. australis* [GCA_029891345.1] [63]), one locust (*S. nitens* [GCA_023898315.2]), one cockroach (*Periplaneta americana* [GCA_025594305.2] [67]), one earwig (*Forficula auricularia* [68]), one aphid (*Aphis gossypii* [GCA_020184165.1] [69]), one model insects (*D. melanogaster* [GCA_000001215.4] [49]). The protein sequences showing redundancy caused by alternative splicing variations or premature codons were removed from the protein-coding genes. OrthoFinder (RRID:SCR_017118) (version 2.5.2) [70] was used to search orthologous groups for protein sequences of eight insect genomes. The protein sequences of 1:1 orthologues in all eight species wer e aligned with MAFFT (RRID:SCR_011811) (version 7.487) [71] software and then non-conservative and unreliable aligned areas were removed using trimAI (RRID:SCR_017334) (version 1.4) [72] with “-gt 0.5”. The filtered protein sequences of 1:1 orthologues were concatenated to generate a Pseudogene sequence. Raxml

(RRID: SCR_006086) (version 8.2.10) [73] software was used to construct a phylogenetic tree of species for the Pseudogene sequence using the "PROTGAMMAWAG" model with 100 bootstrap replicates. The mcmctree program of the PAML (RRID:SCR_014932) (version 4.8) [74] package was used to estimate species divergence times based on 4dTV sites extracted from 1:1 orthologues and five fossil calibration points from the TimeTree (RRID:SCR_021162) database [75] and fossil records [13, 76].

Gene family, positive selection genes and rapid evolution genes

According to orthologous gene clusters and divergence times, café (RRID:SCR_005983) (version 4.2.1) [77] was used to identify the expansion and contraction of gene families for *C. westwoodii* with results from OrthoFinder (RRID:SCR_017118) (version 2.5.2) [70] and the phylogenetic tree with divergence times as inputs.

To analyze positive selection genes and rapid evolution genes, the coding-sequences sequences of 1:1 orthologues in eight species were extracted to align with PRANK (RRID:SCR_017228) (version 170427) [78] and then aligned regions with gaps were was removed using Gblocks (RRID:SCR_015945) (version 0.91b) [79] software with the "-t=c -b5=n". The PAML (RRID:SCR_014932) (version 4.8) [74] software based on the branch-site model and the branch model was used to identify positive selection genes and rapid evolution genes, respectively. Firstly, one ratio model was used to calculate the evolutionary rate ω (*Ka*/*Ks*) of each ortholog in each specie. The branch-

site model was used to detect positive selection signals of genes within *C. westwoodii*. A likelihood ratio test (LRT) was used to compare the alternative hypothesis model that allowed sites to be under positive selection on the foreground branch (*C. westwoodii*) with a null hypothesis model that all sites could to be under purifying or neutral selection. *P-value* of each gene was calculated based on Chi-square statistics and genes with *P-value* less than 0.05 are defined as positive selection genes. Similarly, the branch model was used to detect rapid evolution genes. The LRT with df=1 was performed to compare the fit of the null model that all branches have be assummed to have the same evolutionary rate and the alternative model that allowed the foreground branch to have a different evolutionary rate. Genes with $P<0.05$ and a higher ω value for the foreground than the background branches were identified as rapidly evolving genes. KEGG and GO enrichment analyses were analyzed by DAVID (RRID:SCR_001881) (version 6.8) [80] and KOBAS (RRID:SCR_006350) (version 3.0) [81] for expansion and contraction of gene families, positive selection genes, and rapidly evolving genes. For these enrichment analyses, the entire annotated genes of *C. westwoodii* served as the background gene set. Significant enriched GO and KEGG terms (FDR-adjusted *p*-value $\leqslant$ 0.05, Benjamini-Hochberg method) were retained.

Genome-wide scanning identification of *Cuticle* gene family

Genome-wide protein sequences of *C. westwoodii*, *T. monikensis*, *D. australis and D. melanogaster* were extracted to scan for candidate *Cuticle* genes using hmmscan from HMMER (RRID:SCR_005305) (version 3.3.1) [82] software with the insect cuticle

protein domain (pfam:PF00379.24). To distinguish and assign these candidate *Cuticle* genes to different *Cuticle* gene subfamilies, multiple alignments of the *Cuticle* genes protein sequences were performed using MAFFT (RRID:SCR_011811) (version 7.487) [71], and the poorly aligned regions and partial gaps were removed with trimAI (RRID:SCR_017334) (version 1.4) [72] (gt = 0.5). Then the filtered alignments were used to construct a phylogenetic tree by Raxml (RRID: SCR_006086) (version 8.2.10) [73] software with options "-f a -x 12345 -N 1000 -p 12345 -m PROTGAMMAJTTX". The phylogenetic tree was displayed and edited using FigTree (RRID:SCR_008515) (version 1.4.4) [83]. To further confirm the authenticity of candidate *Cuticle* genes, BLASTP (RRID:SCR_001010) [51] analyses on the NCBI webserver were used to examine functional information.

Results

Genome assembly

A total of 246 Gb ONT long reads (61.5× coverage), 144 Gb Illumina short reads (36× coverage) and 716 Gb Hi-C reads (173× coverage) were generated to assembly a high-quality chromosome-level genome of *C. westwoodii* (**Supplementary Table S1**). Based on K-mer frequency analysis with Illumina short reads, the genome size was estimated to be 4.19 Gb with a heterozygosity rate of 0.7% (**Supplementary Fig. S1; Supplementary Table S2**). We first used ONT long reads for *de novo* assembly to obtain a draft contig-level assembly with a total size of 5.32 Gb, which consists of 5039 contigs (N50 = 3.26 Mb) (**Supplementary Table S3**). Then, after removing the redundant seuences based on read depth of the draft contigs, we got an assembly with

the genome size reduced to 4.09 Gb, comprising 1665 contig (N50 = 8.99 Mb) (**Supplementary Table S3**). After further polishing the haploid assembly twice using Illumina short reads and then scarffolding the contigs using Hi-C reads, we finally built a chromosome-level reference genome of *C. westwoodii*. 98.27% of assembled genome sequences were found to be successfully anchored in 15 pseudochromosome groups including 13 autosomes, one X sex-chromosome and one candidate B-chromosome (**Fig. 2A; Table 1**). The cytogenetic analyses on cell metaphase confirmed the correctness of these anchored chromosomes and revealed the existence of one unpaired chromosome in the genome of this leaf insect (**Fig. 2B**). This chromosome-level assembly had a total length of 4.12 Gb with a scaffold N50 of 256.87 Mb and 98.6% completeness of the BUSCO analysis with the 4.2 % duplicated genes (**Fig. 2C; Table 1; Supplementary Table S3**). In addition, 93.56% and 99.94% of the chromosome-level genome can be covered by Illumina reads (mapping rate: 94.75%) and by Nanopore reads (mapping rate: 99,98%) (**Table 1**). These results indicate that the newly assembled genome of *C. westwoodii* has relatively high completeness and accuracy.

Genome annotation

The annotation results of two types (tandem repeats and transposable elements) of repeat sequences were statistically analyzed, and a total of 2.29 Gb repeat sequences were obtained, accounting for approximately 55.68% of the assembled genome (**Table 1**). Among them, transposable elements (TEs) were made up of DNA transposons (DNA, 14.09%), long terminal repeat sequences (LTRs, 7.38%), long interspersed nuclear elements (LINEs, 5.15%) and short interspersed nuclear elements (SINEs,

3.40%) in the *C. westwoodii* genome (**Supplementary Table S5**).

By integrating 10 gene-sets predicted based on three *ab initio* predicting models (C. secundus, D. melanogaster and transcripts) by Augustus, six homology-based and one transcriptome prediction methods with different weight values for them, a set of the best gene structure was predicted to contain 19,131 protein-coding genes in the *C. westwoodii* genome (**Table 1; Supplementary Table S6**). The average length of genes, CDS, exons and introns were 70,747 bp, 1,366 bp, 203 bp, and 12,153 bp, respectively, while the average number of exons per gene was 6.71 (**Supplementary Table S7**). The BUSCO completeness of the annotated gene-sets was 91.8% with 3.1% duplicated genes. For function annotation, a total of 63.95 % of predicted genes had blast hits in the functional protein databases (**Table 1; Supplementary Table S8**). Furthermore, 7,621 (49.78%) and 8,254 (53.92%) genes were successfully assigned and mapped to Gene Ontology (GO) terms and KEGG pathways, respectively (**Supplementary Table S8**).

The identification of B-chromosome structure and function

Our assembled data and cytogenetic analysis have suggested a candidate B-chromosome in *C. westwoodii* genome (**Fig. 2A–2B**). To investigate the origin and evolution of the B-chromosome, we first compared collinearity relationship between B-chromosome and other 14 A-chromosomes in *C. westwoodii* genome and detected many homologous gene fragments among them **(Fig. 3A**), suggesting that B-chromosome possibly originated from 14 A-chromosomes. We further compared collinearity relationship of chromosomes between *C. westwoodii* and *D. australis* and

found no any collinearity between B chromosome of *C. westwoodii* and the chromosomes of *D. australis* (**Fig. 3B**).

We then investigated the structure characteristics of B-chromsome in *C. westwoodii* genome. Our data showed that the mean depth of B-chromosomes (107×) was nearly twice that of the autosomes (57–61×) (**Fig. 3C; Supplementary Table S18**), indicating the existence of multiple B-chromosomes. Compared to other A-chromosomes, the ratio of repeated sequences of B-chromosome (60.41%) followed that of the X chromosome, and LTR of B-chromosome had the highest proportion (9.53%) (**Fig. 3D; Supplementary Table S16**). A total of 419 protein coding genes were annotated on the B-chromosome **(Supplementary Table S19)**. GO Enrichment analysis indicated that these genes were enriched in such functions as DNA replication and recombination, cell cycle checkpoint signaling, and chromosome condensation (**Fig. 3E; Supplementary Table S17**), which are key processes for genome integrity and chromosome transmission.

Phylogenetic analyses and orthologue identification

Phylogenomic analysis was performed to test the phylogenetic position of *C. westwoodii*. We compared the protein-coding genes of *C. westwoodii* with other seven insects (two stick insects, one locust, one cockroach, one earwig, one aphid, and one fruit fly). Using OrthoFinder, we identified a total of 124281 genes among the eight species, of which 108069 were clustered into 12724 orthogroups. We also counted the

genes of single-copy and multi-copy orthologs, unique genes, unassigned orthologous genes and other genes for each specie (**Fig. 4**). 841 single-copy orthogroups and 3634 multicopy orthogroups were identified among eight species (**Supplementary Table S9**). In the *C. westwoodii* genome, 1299 genes with no orthology relationship were clustered in 588 unique orthologous groups when compared to the other seven species (**Supplementary Table S9**).

To gain an understanding of *C. westwoodii* genomic evolution, we reconstructed a phylogenomic tree of the eight species based on 841 single-copy orthologs (535158 amino acids). The phylogenetic relationships of eight species were well recovered, with all the nodes being strongly supported (UFB/SH-aLRT = 100/100), indicating a good resolution in the phylogram (**Supplementary Fig. 3**). Meanwhile, a calculation of the estimated divergence time bases on the fourfold degenerate synonymous site (4dTV) of the 841 single-copy genes and five fossil calibration points (**Supplementary Table S20**) indicated that the ancestors of *C. westwoodii* and *D. australis* diverged ～49.67 million years ago (Mya) **(Fig. 4).**

Positive selected genes and rapidly evolved genes

We identified positively selected genes and rapid evolved genes of the *C. westwoodii* from 841 single-copy genes among these eight species using branchsite model and branch model. We identified 153 positively selected genes and 354 rapid evolution genes ($P < 0.05$) in the *C. westwoodii*, of which 97 single-copy genes wereshared

(**Supplementary Tables S10–S11**). Gene function annotation showed that these genes were related to regulation of signaling pathways such as Wnt, EGFR, Notch, Hippo, Hedgehog and to epithelial cell polarization, the development and growth of muscle and eye pigment (**Supplementary Tables S10–S11**). Among them, *garnet* [84, 85] and *ruby* [86]**,** related to pigment transport and deposition in compound eyes, were both positively selected and rapid evolution gene. The proteins encoded by these two genes, together with those proteins encoded by genes *orange* and *carmine* gene, form the adaptor protein 3 (AP-3) complex which transports pigment particles into organelles within cells [87].

Gene family expansion and contraction

We used CAFÉ to study the expansions and contractions of gene families during the evolution of the *C. westwoodii*. With the *C. westwoodii* genome, 278 expanded and 288 contracted gene families were identified (**Fig. 4**). Enrichment analysis of GO revealed that expanded gene families in *C. westwoodii* were enriched in structural constituent of cuticle, skin development, eye pigmentation and pigment biosynthetic process, while contracted gene families were enriched in extracellular matrix, detoxification and cuticle development (**Fig. 5A–5B; Supplementary Tables S12–S13**). In addition, the KEGG enrichment results showed that expanded gene families were enriched in glucuronosyltransferase, cytochrome P450 family 2 subfamily D and cytochrome P450 family 2 subfamily J, while contracted gene families were enriched in cytochrome P450 family 4, glutathione S-transferase and carboxylesterase 2 (**Fig. 5C–5D; Supplementary Tables S14–S15**). These digestion-and detoxification-related KEGG classifications, such as cytochrome P450s, glucuronosyltransferase and trypsin have a

direct effect on the digestion of plant cell walls, as well as detoxification and antibacterial properties [88-90].

Differentially expressed genes (DEGs) in the laterally leaf-like abdominal expansions of female individuals at five developmental stages

Transcriptome sequencing of the fifteen samples at five developmental stages (2nd, 3rd, 5th, 7th and 8th (adults) instar) obtained 101.88 Gb of clean data, with the average Q30 of 94.89%, GC content of 47.02%, and mapping ratios of 94.42% (**Supplementary Table S21**). Using the StringTie assembly program, 158199 transcripts were generated from the transcriptome data, of which 93442 transcripts were predicted to have open reading frames by Transdecoder. These 93442 transcripts were divided into 29238 genes including 15588 newly assembled genes.

To identify the DEGs in the laterally leaf-like abdominal expansions of female individuals at five developmental stages (2nd, 3rd, 5th, 7th and 8th (adults) instar), a total of 10 groups (pairwise comparisons) **(Fig. 6A)** were used for analysis and a total of 4554 differentially expressed genes have been identified. Among them, the most DEGs were identified in the group of 2nd instar vs. 8th instar, while the fewest DEGs were detected in group of the 2nd instar vs. 3rd instar (**Supplementary Fig. 4**). Enrichment analysis of GO revealed that the top 10 GO terms of these DEGs are mainly related to structural constituent of chitin-based cuticle and chitin-based cuticle development (**Fig. 6B; Supplementary Table S22**). These 4554 DEGs were divided into seven expression clusters (C1-C7) (**Fig. 6C**; **Supplementary Table S23**). Notably,

cluster C4 (369 genes) related to structural constituent of chitin-based cuticle and chitin-based cuticle development, exhibited a significant expression peak specifically during the 3rd instar **(Fig. 6C; Supplementary Table S24)**. Interestingly, expanded gene families and contracted gene families in *C. westwoodii* genome were also enriched in structural constituent of cuticle and cuticle development (**Fig. 5A–5B**). The findings highlight a central role for *Cuticle* genes in the laterally leaf-like abdominal expansions of female individuals at the five developmental stages at both genome level and expression level. To further investigate the role of *cuticle* genes in morphological development of leaf insect, we identified *Cuticle* genes in *C. westwoodii* by scanning on the genome and transcriptome. Totally, 113 *Cuticle* genes were identified in the genome of the leaf insect (**Fig. 6D**). In these *Cuticle* genes, nearly half of them (53 genes) were identified to be differentially expressed at five developmental stages (**Supplementary tables S25–S26; Supplementary Fig. 5**). Especially, the cuticle gene called *resilin* with 24 copies was significantly expanded in *C. westwoodii* (**Fig. 6D**), and ten copies of *resilin* were significantly differentially expressed at five developmental stages (**Fig. 6E; Supplementary Tables S25–S26**). Among ten differentially expressed copies of *resilin*, five copies were divided into cluster 4 (C4), three copies into cluster 3 (C3), and two copies into cluster 1 (C1) (**Fig. 6C)**. Two copies of *resilin* were significantly differentially upregulated from the 2nd instar to the 3rd instar during the abdominal expanded and swelling obviously. Therefore, *Cuticle* genes, particularly *resilin,* exhibit stage-specific expression patterns during the developmental process of leaf masquerade in *C. westwoodii.*

Discussion

Leaf insects in the family Phylliidae are the best model for studying the evolution of leaf masquerade because their leaf-like body and extended legs. However, the lack of their genomic resources has limited investigation of genetic mechanism of this phenomenon. Here, we assembled a high-quality genome of *C. westwoodii*, representing the first chromosome-level of assembly in family Phylliidae. Compared with previously published genomes of walking sticks of the order Phasmatodea [63, 66, 91-94], the chromosome-level assembly of *C. westwoodii* represents the highest-quality assembly with the second highest N50 value (N50=256.87 Mb) and the lowest number of scaffolds (n=179) (**Supplementary Table S4). **The assembled genome size of *C. westwoodii* is 4.09 Gb, which is the largest one in the published genomes of the order Phasmatodea (**Supplementary Table S4**). The repetitive elements of *C. westwoodii* occupy 55.86% of its total genome, which is within those of the published genomes (49.29%–63.52%) (**Supplementary Table S4**). The gene features (the length of gene, CDS, exons, introns and the number of exons) of assembled *C. westwoodii* genome are similar to those of other four stick insects (**Supplementary Fig. 2**), suggesting the reliability of gene annotation for *C. westwoodii*.

B-chromosome is a kind of extra or redundant or nonessential chromosome and lacks the ability to recombine and pair with autosomes (A-chromosomes) [95]. It is estimated that about 15% of eukaryotic species have B-chromosome, of which plants is major carriers, followed by insects [96, 97]. However, the complete and high-quality assembly

of B-chromosome in insects is still very rare. Interestingly, an unpaired single chromosome (54774726 bp) was assembled in *C. westwoodii* genome **(Fig. 1A)**, and combined with the results of karyotyping, one unpaired single chromosome was identified in female **(Fig. 1B)**, suggesting that B-chromosome have occurred in *C. westwoodii*. The B-chromosome usually contains a large repetitive sequences (DNA repeats and transposons) in essence [98]. Nevertheless, the proportion of repetitive sequences showed no significant difference between B-chromosome and other A-chromosomes in *C. westwoodii*, suggesting that the proportion of repeats sequences is not an indicator to distinguish B-chromosome from A-chromosomes, but the high density distribution area of repetitive and transposable sequences containing multiple candidate centromere regions within B-chromosome may be the evidences for mediating chromosome breakage and fusion to produce B-chromosome [99]. Besides, the B-chromosome in *C. westwoodii* contains 419 genes and the GO enrichment results showed that these genes were enriched in DNA replication and recombination, cell cycle checkpoint signaling and chromosome condensation. In the B chromosomes of two locusts *Abracris flavolineata* [100] and *Eyprepocnemis plorans* [101], genes directly involved in chromosome formation and cell cycle related functions have also been identified. Thess functional genes are not limited to insects; they has also been also found in the mammals red fox and Chinese raccoon dog [102], lizard *Anolis carolinensis* [103] and fishes including *Astyanax mexicanus*, *Astyanax correntinus* and *Astatotilapia latifasciata* [100, 104, 105]. The studies have shown that these genes encoding cell cycle and chromosome-related functions (such as histones, DNA binding

and packaging proteins) may affect B-chromosome precursor DNA generated by transposition, replication, or rearrangement events in the genomic DNA of A-chromosome to form B-chromatin and its reorganization [98-100]. Therefore, these genes may be critical for the survival and transmission of the B-chromosomes at the early evolutionary stage. In this study, we found some genes that have not been reported on the B chromosome, such as glycoside hydrolase family 28 (the plant cell wall digestion enzymes) [106, 107], Gustatory receptor 26b (Gustatory receptor) [108, 109] and cytochrome P450 6j1 (disintoxication) [89] **(Supplementary Table S19)**. This finding suggests that the majority of genes on B-chromosomes across different species are functionally enriched for cell cycle and chromosome condensation, but each specie also can harbor a unique subset of genes with disparate functions.

Regarding the evolutionary origin of B-chromosomes, a concept has been proposed from cytogenetic methods that the B-chromosomes are derivatives of the A chromosomes. In *D. melanogaster*, it was found that the B-chromosome originated from a single A-chromosome [110] and it is speculated that the mitotic error in the chromosome 4 may have triggered the formation of B-chromosome [111]. In contrast, most genes of B-chromosome in *C. westwoodii* were highly homologous to those in A-chromosomes, but were not aligned to any genes in all chromosomes of the *D. australis*. This result is consistent with the results of maize [112, 113], rye [114], and *Phragmites* australis [99, 115], indicating that the B-chromosome may have a mosaic origin based on homologous gene fragments from A-chromosome.

Like other leaf insects in the family Phylliidae, *C. westwoodii* have a unique abdominal structure with extensions on both terga and sterna [116]. Fossil evidences have indicated that the Middle Jurassic stick insect (*Aclistophasma echinulatum*) [117] and the Mesozoic stick insect (*Elasmophasma stictum*) [118] both showed abdominal extensions on terga, which is considered as an initial manifestation of leaf-like extension masquerade in stick insects. In addition, the first fossil leaf insect (*E. messelense*) [119] presented extensions on the abdominal terga and sterna as in modern leaf insects [120]. Furthermore, extant leaf insect *C. westwoodii* not only has abdominal extensions, but also thoracic and legged extensions [116]. These dataindicate that the origin of abdominal extensions predates other modifications in Phasmatodea [117]. Thus, we focused on expansion of the abdomen at five developmental stages to explore the genetic mechanism of leaf-like masquerade by combining genomic and transcriptomic data. Interestingly, our comparative genomics demonstrated expanded and contracted gene families were functionally enriched in structural constituent of cuticle and skin development, which may affect the development of the cuticle and epidermis in *C. westwoodii*. **(Fig. 5A–5B; Supplementary Tables S12–S13).** Furthermore, transcriptomic data indicated that DEGs at different developmental stages were mainly enriched in structural constituent of chitin-based cuticle and chitin-based cuticle development (**Fig. 6B; Supplementary Table S22**). These findings suggest that *Cuticle* genes may play important roles in the evolution of leaf-like abdominal extension at both genome-level and expression-level. Cuticular proteins (CPs) as the

principal components of the insect cuticle, interact with chitin fibres to form a Bouligand-like structure, playing a crucial role in shaping the body morphology during insect development and the construction of external important parts and organs of insect body [121-123]. For an example, in orchid mantis, the cuticle is the major structural constituent of petal-like femoral lobes on the femur of the mid and hind legs and the extensions of ventral femur are regulated by *cuticle* genes through the Wnt signaling [124]. Thus, we further scrutinized *Cuticle* genes in the genome of *C. westwoodii*. Our data showed that 53 of 113 *Cuticle* genes in the genome of *C. westwoodii* differently expressed at five developmental stages.

Especially, the *Cuticle* gene called *resilin* with 24 copies was significantly expanded in *C. westwoodii*, of which ten were significantly differentially expressed in abdominal tissues at five developmental stages. Among ten differentially expressed copies of *resilin*, five copies were divided into cluster 4 (C4) where the 3rd instar can be an important time node for *cuticle* genes. At this stage from the 2nd instar to the 3rd instar, two copies of *resilin* were significantly differentially upregulation while the abdominal extensions presented obviously enlargement a lot and leaf-like body initially formed. *Resilin* is a structural elastic protein widely distributed in insect exoskeletons, playing a crucial role in their movement and ecological adaptability [125, 126]. A resilin-bearing extensor ligament of legs, wing hinge and wings are involved in jumping and flight [127, 128]. Meanwhile, *resilin* mutations decreases the attachment ability of adhesive pads and leads to slip, which is an extremely hostile for survival adaptability

[129]. In addition, resilin-containing exoskeleton structures are also existed in abdominal cuticle of honey-ants works and queen termites, which seems to assist in the extension of abdomen of the queen termites during physogastry and support the expansion and contraction of the abdomen of *Myrmecocystus mexicanus* during storing fluids [130, 131]. These data suggest that *resilin* may promote the abdominal extensions and the formation of leaf-like body.

Data availability

Genomic assembly sequences and the raw sequencing data (including Nanopore long reads, Illumina short reads, Hi-C reads and RNA-seq reads) of *C. westwoodii* in this study have been submitted to the NCBI DataBase and can be accessed with Bioproject number PRJNA1314718 and PRJNA682332.

Additional Files

Supplementary Table S1. The statistics of sequencing data used for the *Cryptophyllium westwoodii* genome assembly.

Supplementary Table S2. Estimation of genome size using kmerfreq software.

Supplementary Table S3. The statistics for the *Cryptophyllium westwoodii* genome assembly based on different kinds of sequencing data.

Supplementary Table S4. The comparison of assembly metrics among *Cryptophyllium westwoodii* and other five stick insects of the order Phasmatodea.

Supplementary Table S5. The statistics of annotated repeat sequences in the

Cryptophyllium westwoodii genome.

Supplementary Table S6. The evaluation of different types of annotated gene sets in EVM under weight values.

Supplementary Table S7. The statistics of protein-coding genes in the *Cryptophyllium westwoodii* genome.

Supplementary Table S8. The statistics of functional annotation of the predicted protein-coding genes the *Cryptophyllium westwoodii* genome.

Supplementary Table S9. The statistics of five categories orthologous in eight species.

Supplementary Table S10. The statistics of positively selected genes of *Cryptophyllium westwoodii*.

Supplementary Table S11. The statistics of rapidly evolving genes of *Cryptophyllium westwoodii*.

Supplementary Table S12. GO enrichment analysis of expanded genes of *Cryptophyllium westwoodii*.

Supplementary Table S13. GO enrichment analysis of contracted genes of *Cryptophyllium westwoodii*.

Supplementary Table S14. KEGG enrichment analysis of expanded genes of *Cryptophyllium westwoodii*.

Supplementary Table S15. KEGG enrichment analysis of contracted genes of *Cryptophyllium westwoodii*.

Supplementary Table S16. The statistics of annotated repeat sequences among 15 chromosomes in the *Cryptophyllium westwoodii* genome.

Supplementary Table S17. GO enrichment terms significantly among the genes on the B-chromosome.

Supplementary Table S18. The statistical data of each chromosome covered by Nanopore reads sequenced from a male individual in the genome of the *Cryptophyllium westwoodii*.

Supplementary Table S19. The statistics of 419 genes on the B-chromosome.

Supplementary Table S20. Information of the calibration nodes used for calculating the divergence time.

Supplementary Table S21. The statistics of quality of RNA-Seq original sequencing data.

Supplementary Table S22. GO enrichment terms among the significantly differentially expressed genes in the laterally leaf-like abdominal expansions of female individuals at five developmental stages.

Supplementary Table S23. The statistics of expression trend clustering among 4554 DEGs at five developmental stages.

Supplementary table S24. GO enrichment terms among the genes in Cluster2, Cluster3 and Cluster4.

Supplementary Table S25. Expression profiles of *Cuticle* genes at the five developmental stages.

Supplementary Table S26. Significantly differentially expressed genes related to *Cuticle* genes at the five developmental stages.

Figure S1. The statistics of k-mer analysis of *Cryptophyllium westwoodii* genome.

The first peak appearing at a depth of 20-fold is a heterozygous peak whereas the third peak appearing at a depth of 74-fold corresponds to a repeated peak. The second peak appearing at a depth of 48-fold is homozygosity, and the predicted genome size is 4199.09 Mb, with repeat sequences accounting for 75.16% and heterozygosity accounting for 0.7%. The x-axis is depth (×), and the y-axis is the proportion which represents the frequency at that depth divided by the total frequency of all the depth.

Figure S2. Comparisons of gene features among the genomes of five species in order Phasmatodea.

Figure S3. Phylogenetic relationship among the eight species inferred by the amino acid sequences of the 841 single-copy genes.

Figure S4. The number of differentially expressed genes (DEGs) in the laterally leaf-like abdominal expansions of female individuals at the five different developmental stages.

Figure S5. The number of differentially expressed *Cuticle* genes in the laterally leaf-like abdominal expansions of female individuals at the five different developmental stages.

Abbreviations

PBS: phosphate buffered saline; BUSCO: Benchmarking Universal Single-Copy Ortholog; TE: transposable element; BLAST: Basic Local Alignment Search Tool; KEGG: Kyoto Encyclopedia of Genes and Genomes; GO: Gene Ontology; NR: non-redundant protein database; Hi-C: high-throughput chromosome conformation capture;

Gb: gigabase; Mb: megabase; LTRs: long terminal repeat sequences; LINEs: long interspersed nuclear elements; SINEs: short interspersed nuclear elements; NCBI: National Center for Biotechnology Information; CDS: coding sequence; Mya: million years ago; DEGs: differentially expressed genes; Wnt: wingless; EGFR: epidermal growth factor receptor.

Author Contributions

X.L. and W.W. conceived and designed the investigation. Z.D. and C.M. conducted the collection, breeding and photography of the insect. G.L. and R.Z. performed nucleic acid extraction. J.L., Y.H., Z.D., C.M., H.G., W.W., and W.N. conducted karyotype experiments. Z.L. and B.Z. assembled the genome. C.M. and J.H. performed genome annotation. C.M., Z.Z., and Y.W. performed transcriptome analyses. C.M. analyzed the data and wrote the draft manuscript. X.L., C.M., and Z.D. revised the manuscript. All the authors read and approved the final manuscript.

Funding

This work was supported by grants from Yunnan Provincial Science and Technology Department (202401BC070017, 202105AC160039) and Chinese Academy of Sciences (CAS "Light of West China" Program to X.L.).

Acknowledgements

We thank Lei Chen, Keng Wang, Yongxing Li, Ru Zhang and Jie Yang of Northwestern Polytechnical University for providing helps in genome annotation. We thank

Guangping Huang of Institute of Zoology, Chinese Academy of Sciences (Beijing, China) for providing suggestions in collecting the laterally leaf-like abdominal expansions. We thank Kunming Cell Bank, Chinese Academy of Sciences (Kunming, China) and the Animal Bank at the Germplasm Bank of Wild Species for providing technical support.

Competing Interests

The authors declared no competing interests.

References

1. Poulton EB. Natural selection the cause of mimetic resemblance and common warning colours. Zoological Journal of the Linnean Society. 1898;26(172):558–612. http://doi.org/10.1111/j.1096-3642.1898.tb01734.x.

2. Wallace AR. Contributions to the theory of natural selection: a series of essays. London, UK: Macmillan & Co; 1871.

3. Wallace AR. Darwinism: an exposition of the theory of natural selection, with some of its applications. Lodon, UK: Macmillan & Co; 1889.

4. Skelhorn J, Rowland HM, Speed MP, et al. Masquerade: camouflage without crypsis. Science. 2010;327(5961):51. http://doi.org/10.1126/science.1181931.

5. O'Hanlon JC. Orchid mantis. Current Biology. 2016;26(4):145–6. http://doi.org/10.1016/j.cub.2015.11.027.

6. O'Hanlon JC, Holwell GI, Herberstein ME. Pollinator deception in the orchid mantis. The American Naturalist. 2014;183(1):126–32. http://doi.org/10.1086/673858.

7. Zhang Z-T, Yu L, Chang H-Z, et al. Nature's disguise: empirical demonstration of dead-leaf masquerade in Kallima butterflies. Zoological Research. 2024;45(6):1201–8. http://doi.org/10.24272/j.issn.2095-8137.2024.025.

8. Shirozu T, Nakanishi A. A revision of the genus Kallima Doubleday (Lepidoptera, Nymphalidae) : I. Generic classification. Lepidoptera Science. 1984;34(3):97–110. http://doi.org/10.18984/lepid.34.3_97.

9. Bank S, Cumming RT, Li Y, et al. A tree of leaves: Phylogeny and historical biogeography of the leaf insects (Phasmatodea: Phylliidae). Communications Biology. 2021;4(1):932. http://doi.org/10.1038/s42003-021-02436-z.
10. Bradler S, Buckley TR. Biodiversity of Phasmatodea. Insect biodiversity: science and society. 2018;2:281–313. http://doi.org/10.1002/9781118945582.ch11.
11. Shi C, Shih C, Chen S, et al. Phasmatodea–stick insects and leaf insects. In: Ren D, Shih CK, Gao T, editors. Rhythms of insect evolution: evidence from the Jurassic and Cretaceous in Northern China. Phasmatodea–stick insects and leaf insects. Hoboken, USA: Wiley-Blackwell; 2019. p. 165–73. http://doi.org/10.5962/bhl.title.122844.
12. Cliquennois N. ordre des Phasmatodea (Phasmes). In: Aberlenc HP, editor. Les Insectes du Monde. Biodiversité. Classification. détermination des familles. Versailles, France: Éditions Quae & Museo; 2020. p. 1848. http://doi.org/https://doi.org/10.5962/bhl.title.122844.
13. Boisseau RP, Bradler S, Emlen DJ. Divergence time and environmental similarity predict the strength of morphological convergence in stick and leaf insects. Proceedings of the National Academy of Sciences. 2025;122(1):e2319485121. http://doi.org/10.1073/pnas.2319485121.
14. Brock PD, Büscher TH, Baker E. Phasmida Species File Online. 2022. https://phasmida.speciesfile.org. Accessed 31 December 2022.
15. Cumming RT, Bank S, Bresseel J, et al. Cryptophyllium, the hidden leaf insects–descriptions of a new leaf insect genus and thirteen species from the former celebicum species group (Phasmatodea, Phylliidae). ZooKeys. 2021;1018:1–179. http://doi.org/10.3897/zookeys.1018.61033. .
16. Wood-Mason J. On new or little-known species of Phasmidae, with a brief preliminary notice of the occurrence of a clasping apparatus in the males throughout the family. Journal of the Asiatic Society of Bengal. 1875;44(2):215–20.
17. Imai HT, TAYLoR RW, Crosland MW, et al. Modes of spontaneous chromosomal mutation and karyotype evolution in ants with reference to the minimum interaction hypothesis. The Japanese journal of genetics. 1988;63(2):159–85. http://doi.org/10.1266/jjg.63.159.
18. Lukhtanov VA, Dantchenko AV. A new butterfly species from south Russia revealed through chromosomal and molecular analysis of the Polyommatus (Agrodiaetus) damonides complex (Lepidoptera, Lycaenidae). Comparative

Cytogenetics. 2017;11(4):769–95. http://doi.org/10.3897/CompCytogen.v11i4.20072.

19. Chen S, Zhou Y, Chen Y, et al. fastp: an ultra-fast all-in-one FASTQ preprocessor. Bioinformatics. 2018;34(17):884–90. http://doi.org/10.1093/bioinformatics/bty560.

20. Xu H, Luo X, Qian J, et al. FastUniq: a fast de novo duplicates removal tool for paired short reads. PloS one. 2012;7(12):e52249. http://doi.org/10.1371/journal.pone.0052249.

21. Liu B, Shi Y, Yuan J, et al. Estimation of genomic characteristics by analyzing k-mer frequency in de novo genome projects. Quantitative Biology. 2013;35(Suppl. 1–3):62–7. http://doi.org/10.1016/S0925-4005(96)02015-1.

22. Belton J-M, McCord RP, Gibcus JH, et al. Hi–C: a comprehensive technique to capture the conformation of genomes. Methods. 2012;58(3):268–76. http://doi.org/10.1016/j.ymeth.2012.05.001.

23. Lieberman-Aiden E, Van Berkum NL, Williams L, et al. Comprehensive mapping of long-range interactions reveals folding principles of the human genome. science. 2009;326(5950):289–93. http://doi.org/10.1126/science.1181369.

24. Bolger AM, Lohse M, Usadel B. Trimmomatic: a flexible trimmer for Illumina sequence data. Bioinformatics. 2014;30(15):2114–20. http://doi.org/10.1093/bioinformatics/btu170.

25. Kim D, Langmead B, Salzberg SL. HISAT: a fast spliced aligner with low memory requirements. Nature methods. 2015;12(4):357–60. http://doi.org/10.1038/nmeth.3317.

26. Pertea M, Pertea GM, Antonescu CM, et al. StringTie enables improved reconstruction of a transcriptome from RNA-seq reads. Nature biotechnology. 2015;33(3):290–5. http://doi.org/10.1038/nbt.3122. .

27. Kumar L, Futschik ME. Mfuzz: a software package for soft clustering of microarray data. Bioinformation. 2007;2(1):5–7. http://doi.org/10.6026/97320630002005.

28. Zhang J. ClusterGVis: one-step to cluster and visualize gene expression matrix. 2020. https://github.com/junjunlab/ClusterGVis. Accessed 9 December 2022.

29. Love MI, Huber W, Anders S. Moderated estimation of fold change and dispersion for RNA-seq data with DESeq2. Genome biology. 2014;15(12):550. http://doi.org/10.1186/s13059-014-0550-8.

30. Hu J, Wang Z, Sun Z, et al. NextDenovo: an efficient error correction and accurate assembly tool for noisy long reads. Genome Biology. 2024;25(1):107.

http://doi.org/10.1186/s13059-024-03252-4.
31. Guan D, McCarthy SA, Wood J, et al. Identifying and removing haplotypic duplication in primary genome assemblies. Bioinformatics. 2020;36(9):2896–8. http://doi.org/10.1093/bioinformatics/btaa025.
32. Hu J, Fan J, Sun Z, et al. NextPolish: a fast and efficient genome polishing tool for long-read assembly. Bioinformatics. 2020;36(7):2253–5. http://doi.org/10.1093/bioinformatics/btz891.
33. Durand NC, Shamim MS, Machol I, et al. Juicer provides a one-click system for analyzing loop-resolution Hi-C experiments. Cell systems. 2016;3(1):95–8. http://doi.org/10.1016/j.cels.2016.07.002.
34. Dudchenko O, Batra SS, Omer AD, et al. De novo assembly of the Aedes aegypti genome using Hi-C yields chromosome-length scaffolds. Science. 2017;356(6333):92–5. http://doi.org/10.1126/science.aal3327.
35. Robinson JT, Turner D, Durand NC, et al. Juicebox. js provides a cloud-based visualization system for Hi-C data. Cell systems. 2018;6(2):256–8. http://doi.org/10.1016/j.cels.2018.01.001.
36. Li H, Durbin R. Fast and accurate short read alignment with Burrows–Wheeler transform. bioinformatics. 2009;25(14):1754–60. http://doi.org/10.1093/bioinformatics/btp324.
37. Li H. Minimap2: pairwise alignment for nucleotide sequences. Bioinformatics. 2018;34(18):3094–100. http://doi.org/10.1093/bioinformatics/bty191.
38. Danecek P, Bonfield JK, Liddle J, et al. Twelve years of SAMtools and BCFtools. Gigascience. 2021;10(2):giab008. http://doi.org/10.1093/gigascience/giab008.
39. Simão FA, Waterhouse RM, Ioannidis P, et al. BUSCO: assessing genome assembly and annotation completeness with single-copy orthologs. Bioinformatics. 2015;31(19):3210–2. http://doi.org/10.1093/bioinformatics/btv351.
40. Manni M, Berkeley MR, Seppey M, et al. BUSCO update: novel and streamlined workflows along with broader and deeper phylogenetic coverage for scoring of eukaryotic, prokaryotic, and viral genomes. Molecular biology and evolution. 2021;38(10):4647–54. http://doi.org/10.1093/molbev/msab199.
41. Xu Z, Wang H. LTR_FINDER: an efficient tool for the prediction of full-length LTR retrotransposons. Nucleic acids research. 2007;35(2):265–8. http://doi.org/10.1093/nar/gkm286.

42. Benson G. Tandem repeats finder: a program to analyze DNA sequences. Nucleic acids research. 1999;27(2):573–80. http://doi.org/10.1093/nar/27.2.573.

43. Bedell JA, Korf I, Gish W. MaskerAid: a performance enhancement to RepeatMasker. Bioinformatics. 2000;16(11):1040–1. http://doi.org/10.1093/bioinformatics/16.11.1040.

44. Chen N. Using Repeat Masker to identify repetitive elements in genomic sequences. Current protocols in bioinformatics. 2004;5(1):4–10. http://doi.org/10.1002/0471250953.bi0410s05.

45. Flynn JM, Hubley R, Goubert C, et al. RepeatModeler2 for automated genomic discovery of transposable element families. Proceedings of the National Academy of Sciences. 2020;117(17):9451–7. http://doi.org/10.1073/pnas.1921046117.

46. Terrapon N, Li C, Robertson HM, et al. Molecular traces of alternative social organization in a termite genome. Nature communications. 2014;5(1):3636. http://doi.org/10.1038/ncomms4636.

47. Harrison MC, Jongepier E, Robertson HM, et al. Hemimetabolous genomes reveal molecular basis of termite eusociality. Nature ecology & evolution. 2018;2(3):557–66. http://doi.org/10.1038/s41559-017-0459-1.

48. Shigenobu S, Hayashi Y, Watanabe D, et al. Genomic and transcriptomic analyses of the subterranean termite Reticulitermes speratus: gene duplication facilitates social evolution. Proceedings of the National Academy of Sciences. 2022;119(3):e2110361119. http://doi.org/10.1073/pnas.2110361119.

49. Langley CH, Crepeau M, Cardeno C, et al. Circumventing heterozygosity: sequencing the amplified genome of a single haploid Drosophila melanogaster embryo. Genetics. 2011;188(2):239–46. http://doi.org/10.1534/genetics.111.127530.

50. Kim HS, Murphy T, Xia J, et al. BeetleBase in 2010: revisions to provide comprehensive genomic information for Tribolium castaneum. Nucleic acids research. 2010;38(suppl_1):437–42. http://doi.org/10.1093/nar/gkp807.

51. Altschul SF, Gish W, Miller W, et al. Basic local alignment search tool. Journal of molecular biology. 1990;215(3):403–10. http://doi.org/10.1016/S0022-2836(05)80360-2.

52. Yu X-J, Zheng H-K, Wang J, et al. Detecting lineage-specific adaptive evolution of brain-expressed genes in human using rhesus macaque as outgroup. Genomics. 2006;88(6):745–51. http://doi.org/10.1016/j.ygeno.2006.05.008.

53. Birney E, Clamp M, Durbin R. GeneWise and genomewise. Genome research. 2004;14(5):988–95. http://doi.org/10.1101/gr.1865504.

54. Duarte GT, Volkova PY, Geras'kin SA. A pipeline for non-model organisms for de novo transcriptome assembly, annotation, and gene ontology analysis using open tools: case study with scots pine. Bio-protocol. 2021;11(3):e3912. http://doi.org/10.21769/BioProtoc.3912.

55. Haas BJ, Delcher AL, Mount SM, et al. Improving the Arabidopsis genome annotation using maximal transcript alignment assemblies. Nucleic acids research. 2003;31(19):5654–66. http://doi.org/10.1093/nar/gkg770.

56. Stanke M, Keller O, Gunduz I, et al. AUGUSTUS: ab initio prediction of alternative transcripts. Nucleic acids research. 2006;34(suppl_2):435–9. http://doi.org/10.1093/nar/gkl200.

57. Haas BJ, Salzberg SL, Zhu W, et al. Automated eukaryotic gene structure annotation using EVidenceModeler and the Program to Assemble Spliced Alignments. Genome biology. 2008;9(1):R7. http://doi.org/10.1186/gb-2008-9-1-r7.

58. O'Donovan C, Martin MJ, Gattiker A, et al. High-quality protein knowledge resource: SWISS-PROT and TrEMBL. Briefings in bioinformatics. 2002;3(3):275–84. http://doi.org/10.1093/bib/3.3.275.

59. Yip YL, Scheib H, Diemand AV, et al. The Swiss-Prot variant page and the ModSNP database: a resource for sequence and structure information on human protein variants. Human mutation. 2004;23(5):464–70. http://doi.org/10.1002/humu.20021.

60. Kanehisa M, Goto S, Kawashima S, et al. The KEGG databases at GenomeNet. Nucleic acids research. 2002;30(1):42–6. http://doi.org/10.1093/nar/30.1.42.

61. Yangyang D, Jianqi LI, Songfeng WU, et al. Integrated nr Database in Protein Annotation System and Its Localization. Computer Engineering. 2006;32(5):71–2. http://doi.org/10.3969/j.issn.1000-3428.2006.05.026.

62. Zdobnov EM, Apweiler R. InterProScan–an integration platform for the signature-recognition methods in InterPro. Bioinformatics. 2001;17(9):847–8. http://doi.org/10.1093/bioinformatics/17.9.847.

63. Stuart OP, Cleave R, Magrath MJ, et al. Genome of the Lord Howe Island stick insect reveals a highly conserved phasmid X chromosome. Genome Biology and Evolution. 2023;15(6):evad104. http://doi.org/10.1093/gbe/evad104.

64. Wang Y, Tang H, DeBarry JD, et al. MCScanX: a toolkit for detection and evolutionary analysis of gene synteny and collinearity. Nucleic acids research. 2012;40(7):e49. http://doi.org/10.1093/nar/gkr1293.

65. Krzywinski M, Schein J, Birol I, et al. Circos: an information aesthetic for comparative genomics. Genome research. 2009;19(9):1639–45. http://doi.org/10.1101/gr.092759.109.

66. Jaron KS, Parker DJ, Anselmetti Y, et al. Convergent consequences of parthenogenesis on stick insect genomes. Science advances. 2022;8(8):eabg3842. http://doi.org/10.1126/sciadv.abg3842.

67. Wang L, Xiong Q, Saelim N, et al. Genome assembly and annotation of Periplaneta americana reveal a comprehensive cockroach allergen profile. Allergy. 2023;78(4):1088–103. http://doi.org/10.1111/all.15531.

68. Bhattarai UR, Katuwal M, Poulin R, et al. Genome assembly and annotation of the European earwig Forficula auricularia (subspecies B). G3. 2022;12(10):jkac199. http://doi.org/10.1093/g3journal/jkac199.

69. Zhang S, Gao X, Wang L, et al. Chromosome-level genome assemblies of two cotton-melon aphid Aphis gossypii biotypes unveil mechanisms of host adaption. Molecular ecology resources. 2022;22(3):1120–34. http://doi.org/10.1111/1755-0998.13521.

70. Emms DM, Kelly S. OrthoFinder: solving fundamental biases in whole genome comparisons dramatically improves orthogroup inference accuracy. Genome biology. 2015;16(1):157. http://doi.org/10.1186/s13059-015-0721-2.

71. Katoh K, Misawa K, Kuma Ki, et al. MAFFT: a novel method for rapid multiple sequence alignment based on fast Fourier transform. Nucleic acids research. 2002;30(14):3059–66. http://doi.org/10.1093/nar/gkf436.

72. Capella-Gutiérrez S, Silla-Martínez JM, Gabaldón T. trimAl: a tool for automated alignment trimming in large-scale phylogenetic analyses. Bioinformatics. 2009;25(15):1972–3. http://doi.org/10.1093/bioinformatics/btp348. .

73. Stamatakis A. RAxML version 8: a tool for phylogenetic analysis and post-analysis of large phylogenies. Bioinformatics. 2014;30(9):1312–3. http://doi.org/10.1093/bioinformatics/btu033.

74. Yang Z. PAML 4: phylogenetic analysis by maximum likelihood. Molecular

biology and evolution. 2007;24(8):1586–91. http://doi.org/10.1093/molbev/msm088.

75. Hedges SB, Dudley J, Kumar S. TimeTree: a public knowledge-base of divergence times among organisms. Bioinformatics. 2006;22(23):2971–2. http://doi.org/10.1093/bioinformatics/btl505.

76. Simon S, Letsch H, Bank S, et al. Old world and new world Phasmatodea: phylogenomics resolve the evolutionary history of stick and leaf insects. Frontiers in Ecology and Evolution. 2019;7:345. http://doi.org/10.3389/fevo.2019.00345.

77. De Bie T, Cristianini N, Demuth JP, et al. CAFE: a computational tool for the study of gene family evolution. Bioinformatics. 2006;22(10):1269–71. http://doi.org/10.1093/bioinformatics/btl097.

78. Löytynoja A. Phylogeny-aware alignment with PRANK. Methods Mol Biol 2014;1079:155–70. http://doi.org/10.1007/978-1-62703-646-7_10.

79. Castresana J. Selection of conserved blocks from multiple alignments for their use in phylogenetic analysis. Molecular biology and evolution. 2000;17(4):540–52. http://doi.org/10.1093/oxfordjournals.molbev.a026334.

80. Dennis Jr G, Sherman BT, Hosack DA, et al. DAVID: database for annotation, visualization, and integrated discovery. Genome biology. 2003;4(9):R60. http://doi.org/10.1186/gb-2003-4-5-p3.

81. Xie C, Mao X, Huang J, et al. KOBAS 2.0: a web server for annotation and identification of enriched pathways and diseases. Nucleic acids research. 2011;39(2):316–22. http://doi.org/10.1093/nar/gkr483.

82. Stanke M, Waack S. Gene prediction with a hidden Markov model and a new intron submodel. Bioinformatics-Oxford. 2003;19(2):215–25. http://doi.org/10.1093/bioinformatics/btg1080.

83. Figtree. Figtree (Version 1.4.4). 2018. http://tree.bio.ed.ac.uk/software/Figtree. Accessed 26 November 2018.

84. Lloyd VK, Sinclair D, Wennberg R, et al. A genetic and molecular characterization of the garnet gene of Drosophila melanogaster. Genome. 1999;42(6):1183–93. http://doi.org/10.1139/g99-088.

85. Ooi CE, Moreira JE, Dell'Angelica EC, et al. Altered expression of a novel adaptin leads to defective pigment granule biogenesis in the Drosophila eye color mutant garnet. The EMBO journal. 1997;16(15):4508–18. http://doi.org/10.1093/emboj/16.15.4508.

86. Kretzschmar D, Poeck B, Roth H, et al. Defective pigment granule biogenesis and

aberrant behavior caused by mutations in the Drosophila AP-3β adaptin gene ruby. Genetics. 2000;155(1):213–23. http://doi.org/10.1093/genetics/155.1.213.

87. Mullins C, Hartnell L, Bonifacino J. Distinct requirements for the AP-3 adaptor complex in pigment granule and synaptic vesicle biogenesis in Drosophila melanogaster. Molecular and General Genetics MGG. 2000;263(6):1003–14. http://doi.org/10.1007/pl00008688.

88. Muhlia-Almazán A, Sánchez-Paz A, García-Carreño FL. Invertebrate trypsins: a review. Journal of Comparative Physiology B. 2008;178(6):655–72. http://doi.org/10.1007/s00360-008-0263-y.

89. Scott JG, Liu N, Wen Z. Insect cytochromes P450: diversity, insecticide resistance and tolerance to plant toxins. Comparative Biochemistry and Physiology Part C: Pharmacology, Toxicology and Endocrinology. 1998;121(1-3):147–55. http://doi.org/10.1016/s0742-8413(98)10035-x.

90. Bock KW. The UDP-glycosyltransferase (UGT) superfamily expressed in humans, insects and plants: Animal-plant arms-race and co-evolution. Biochemical pharmacology. 2016;99:11–7. http://doi.org/10.1016/j.bcp.2015.10.001.

91. Choi SS, Mc Cartney A, Park D, et al. Multiple hybridization events and repeated evolution of homoeologue expression bias in parthenogenetic, polyploid New Zealand stick insects. Molecular Ecology. 2023:e17422. http://doi.org/10.1111/mec.17422.

92. Chen W, Twort VG, Crowhurst RN, et al. Assembling large genomes: analysis of the stick insect (Clitarchus hookeri) genome reveals a high repeat content and sex-biased genes associated with reproduction. Bmc Genomics. 2017;18(1):884. http://doi.org/10.1186/s12864-017-4245-x.

93. Lavanchy G, Brandt A, Bastardot M, et al. Evolution of alternative reproductive systems in Bacillus stick insects. Evolution. 2024;78(6):1109–20. http://doi.org/10.1093/evolut/qpae045.

94. Brand P, Lin W, Johnson BR. The draft genome of the invasive walking stick, Medauroidea extradendata, reveals extensive lineage-specific gene family expansions of cell wall degrading enzymes in phasmatodea. G3: Genes, Genomes, Genetics. 2018;8(5):1403–8. http://doi.org/10.1534/g3.118.200204.

95. Longley AE. Supernumerary chromosomes in Zea mays. J Agric Res. 1927;35:769–84.

96. D'Ambrosio U, Alonso-Lifante MP, Barros K, et al. B-chrom: a database on B-

chromosomes of plants, animals and fungi. New Phytologist. 2017;216(3):635–42. http://doi.org/10.1111/nph.
97. Beukeboom, Leo W. Bewildering Bs: an impression of the 1st B-chromosome conference. Heredity. 1994;73(3):328–36. http://doi.org/10.1038/hdy.1994.140.
98. Ahmad SF, Martins C. The modern view of B chromosomes under the impact of high scale omics analyses. Cells. 2019;8(2):156. http://doi.org/10.3390/cells8020156.
99. Cui J, Wang R, Gu R, et al. Telomere-to-telomere Phragmites australis reference genome assembly with a B chromosome provides insights into its evolution and polysaccharide biosynthesis. Communications Biology. 2025;8(1):73. http://doi.org/10.1038/s42003-025-07532-y.
100. Ahmad SF, Jehangir M, Cardoso AL, et al. B chromosomes of multiple species have intense evolutionary dynamics and accumulated genes related to important biological processes. BMC Genomics. 2020;23(1):656. http://doi.org/10.1186/s12864-020-07072-1.
101. Beatriz.Navarro-Domínguez, Ruiz-Ruano FJ, Cabrero J, et al. Protein-coding genes in B chromosomes of the grasshopper Eyprepocnemis plorans. Scientific reports. 2017;7:45200. http://doi.org/10.1038/srep45200.
102. Alexey M, Svetlana R, Violetta B, et al. Sequencing of supernumerary chromosomes of red fox and raccoon dog confirms a non-random gene acquisition by B chromosomes. Genes. 2018;9(8):405. http://doi.org/10.3390/genes9080405.
103. Kichigin IG, Lisachov AP, Giovannotti M, et al. First report on B chromosome content in a reptilian species: the case of Anolis carolinensis. Molecular genetics and genomics : MGG. 2019;294(1):13–21. http://doi.org/10.1007/s00438-018-1483-9.
104. Jehangir M, Ahmad SF, Cardoso AL, et al. De novo genome assembly of the cichlid fish Astatotilapia latifasciata reveals a higher level of genomic polymorphism and genes related to B chromosomes. Chromosoma. 2019;128(2):81–96. http://doi.org/10.1007/s00412-019-00707-7.
105. Valente GT, Conte MA, Fantinatti BE, et al. Origin and evolution of B chromosomes in the cichlid fish Astatotilapia latifasciata based on integrated genomic analyses. Molecular biology and evolution. 2014;31(8):2061–72. http://doi.org/10.1093/molbev/msu148.
106. Shelomi M, Danchin EGJ, Heckel D, et al. Horizontal gene transfer of

pectinases from bacteria preceded the diversification of stick and leaf insects. Scientific Reports. 2016;6:26388. http://doi.org/10.1038/srep26388.

107. Wu C, Crowhurst RN, Dennis AB, et al. De novo transcriptome analysis of the common New Zealand stick insect Clitarchus hookeri (Phasmatodea) reveals genes involved in olfaction, digestion and sexual reproduction. PLoS One. 2016;11(6):e0157783. http://doi.org/10.1371/journal.pone.0157783.

108. Ahn JE, Amrein H. Opposing chemosensory functions of closely related gustatory receptors. eLife. 2023;12:RP89795. http://doi.org/10.7554/eLife.89795.

109. Miyamoto T, Slone J, Song X, et al. A fructose receptor functions as a nutrient sensor in the Drosophila brain. Cell. 2012;151(5):1113–25. http://doi.org/10.1016/j.cell.2012.10.024.

110. Bauerly E, Hughes SE, Vietti DR, et al. Discovery of supernumerary B chromosomes in Drosophila melanogaster. Genetics. 2014;196(4):1007–16. http://doi.org/10.1534/genetics.113.160556.

111. Hanlon SL, Hawley RS. B chromosomes in the Drosophila genus. Genes. 2018;9(10):470. http://doi.org/10.3390/genes9100470.

112. Liu Q, Liu Y, Yi C, et al. Genome assembly of the maize B chromosome provides insight into its epigenetic characteristics and effects on the host genome. Genome Biology. 2025;26(1):47. http://doi.org/10.1186/s13059-025-03517-6.

113. Blavet N, Yang H, Su H, et al. Sequence of the supernumerary B chromosome of maize provides insight into its drive mechanism and evolution. Proceedings of the National Academy of Sciences. 2021;118(23):e2104254118. http://doi.org/10.1073/pnas.2104254118.

114. Chen J, Bartoš J, Boudichevskaia A, et al. The genetic mechanism of B chromosome drive in rye illuminated by chromosome-scale assembly. Nature Communications. 2024;15(1):9686. http://doi.org/10.1038/s41467-024-53799-w.

115. Wang C, Liu L, Yin M, et al. Chromosome-level genome assemblies reveal genome evolution of an invasive plant Phragmites australis. Communications Biology. 2024;7(1):1007. http://doi.org/10.1038/s42003-024-06660-1.

116. Hennemann FH, Conle OV, Gottardo M, et al. On certain species of the genus Phyllium Illiger, 1798, with proposals for an intra-generic systematization and the descriptions of five new species from the Philippines and Palawan (Phasmatodea: Phylliidae: Phylliinae: Phylliini). Zootaxa. 2009;2322(1):1–83.

http://doi.org/10.11646/zootaxa.2322.1.1.
117. Yang H, Shi C, Engel MS, et al. Early specializations for mimicry and defense in a Jurassic stick insect. National Science Review. 2021;8(1):nwaa056. http://doi.org/10.1093/nsr/nwaa056.
118. Chen S, Yin X, Lin X, et al. Stick insect in Burmese amber reveals an early evolution of lateral lamellae in the Mesozoic. Proceedings of the Royal society B: Biological Sciences. 2018;285(1877):20180425. http://doi.org/10.1098/rspb.2018.0425.
119. Wedmann S, Bradler S, Rust J. The first fossil leaf insect: 47 million years of specialized cryptic morphology and behavior. Proceedings of the National Academy of Sciences. 2007;104(2):565–9. http://doi.org/10.1073/pnas.0606937104.
120. Zompro O, Größer D. A generic revision of the insect order Phasmatodea: The genera of the areolate stick insect family Phylliidae (Walking Leaves)(Insecta, Orthoptera). Spixiana. 2003;26(2):129–41. http://doi.org/10.1103/PhysRevA.36.1929.
121. Tajiri R, Ogawa N, Fujiwara H, et al. Mechanical control of whole body shape by a single cuticular protein Obstructor-E in Drosophila melanogaster. PLoS genetics. 2017;13(1):e1006548. http://doi.org/10.1371/journal.pgen.1006548.
122. Muthukrishnan S, Mun S, Noh MY, et al. Insect cuticular chitin contributes to form and function. Current pharmaceutical design. 2020;26(29):3530–45. http://doi.org/10.2174/1381612826666200523175409.
123. Zhou Y, Badgett MJ, Bowen JH, et al. Distribution of cuticular proteins in different structures of adult Anopheles gambiae. Insect biochemistry and molecular biology. 2016;75:45–57. http://doi.org/10.1016/j.ibmb.2016.05.001.
124. Huang G, Song L, Du X, et al. Evolutionary genomics of camouflage innovation in the orchid mantis. Nature Communications. 2023;14(1):4821. http://doi.org/10.1038/s41467-023-40355-1.
125. Elvin CM, Carr AG, Huson MG, et al. Synthesis and properties of crosslinked recombinant pro-resilin. Nature. 2005;437(7061):999–1002. http://doi.org/10.1038/nature04085.
126. Michels J, Appel E, Gorb SN. Functional diversity of resilin in Arthropoda. Beilstein Journal of Nanotechnology. 2016;7(1):1241–59. http://doi.org/10.3762/bjnano.7.115.
127. Lerch S, Zuber R, Gehring N, et al. Resilin matrix distribution, variability and

function in Drosophila. BMC biology. 2020;18(1):195. http://doi.org/10.1186/s12915-020-00902-4.

128. Rogers SM, Cullen DA, Labonte D, et al. RNAi of the elastomeric protein resilin reduces jump velocity and resilience to damage in locusts. Proceedings of the National Academy of Sciences. 2025;122(1):e2415625121. http://doi.org/10.1073/pnas.2415625121.

129. Dong H, Yan J, Wang X, et al. Mutation in Resilin reveals attachment impairment in Bombyx mori. Insect Science. 2025. http://doi.org/10.1111/1744-7917.70002.

130. Varman AR. Resilin in the abdominal cuticle of workers of the honey-ants. Journal of the Georgia Entomological Society. 1981;16(1):11–3.

131. Varman AR. Resilin in the cuticle of physogastric queen termites. Experientia. 1980;36(5):564. http://doi.org/10.1007/BF01965802.

Figure legends and Tables

Figure 1: A female leaf insect (*Cryptophyllium westwoodii*). Photo by Zhiwei Dong.

Figure 2: Genome description of *Cryptophyllium westwoodii*. (A) Hi-C interaction map produced by 3D-DNA. **(B)** The Karyotype of female and male adults. During meiotic metaphase, 29 chromosomes (one pair X sex-chromosomes, one B-chromosome (denoted by red arrow) and 13 pairs autosomes) were observed in females, and 27 chromosomes (one X sex-chromosome and 13 pairs autosomes) were observed in males. **(C)** Circos plot of chromosome-level genome. Tracks represent the distribution of GC density, gene density and repeat sequences density, respectively. Densities were calculated in 100-kb window.

Figure 3: B chromosome structure and function of *Cryptophyllium westwoodii*. (A) The synteny analysis between B chromosome and other 14 A chromosomes in the genome of *C. westwoodii*. **(B)** The synteny analysis of chromosomes between *C. westwoodii* (Cwe) and *Dryococelus australis* (Dau). **(C)** The Nanopore sequence depth of each chromosome was plotted with a window of 100-kp in a male individual of *C. westwoodii*. **(D)** The statistics of the proportion of repetitive sequence types for each chromosome in *C. westwoodii*. **(E)** GO Enrichment analysis of the protein coding genes on the B chromosome in *C. westwoodii*.

Figure 4: Phylogenetic and evolutionary analyses of *Cryptophyllium westwoodii* genome. In the left panel, blue and red numbers on the branch show the number of expanded and contracted gene families for each clade. The black numbers are divergence times. In the right panel, the numbers of gene families (orthogroups) are shown as barplots. Orthogroups of different categories are in different colors.

Figure 5: Enrichment analysis of gene families of *Cryptophyllium westwoodii*. (A) GO enrichment of expanded gene families. **(B)** GO enrichment of contracted gene families. **(C)** KEGG pathway of expanded gene families. **(D)** KEGG pathway of contracted gene families.

Figure 6: Differentially expressed genes at five different developmental stages of *Cryptophyllium westwoodi*. (A) Habitus of female individuals at the five different developmental stages for transcriptome sequencing. Photos by Zhiwei Dong. **(B)** GO Enrichment analysis of 4554 DEGs in the laterally leaf-like abdominal expansions of female individuals at the five different

developmental stages in **(A)**. **(C)** The gene expression trend clustering analysis of 4454 DEGs. The figure consists of a line chart, heatmap and GO enrichment information. The line chart and heatmap show the gene expression trend and level in each cluster, while the top five significantly GO enrichment term in 3 clusters are shown on the right side of the heatmap. **(D)** Phylogenetic analysis of *Cuticle* gene family among *Drosophila melanogaster* (Dme), *Timema monikensis* (Tms), *Drycocelus australis* (Dau) and *Cryptophyllium westwoodii* (Cwe). **(E)** Heatmaps of *resilin* gene with 24 copies in the laterally leaf-like abdominal expansions at the five developmental stages. F2: second instar larvae, F3: third instar larvae, F5: fifth instar larvae, F6: sixth instar larvae, F8: eighth instar (adults). The red and blue colors in panels **(C), (E)** indicate high and low expression levels, respectively.

Table 1: The statistics of genome assembly and annotation in *Cryptophyllium westwoodii* genome.

Features	*C. westwoodii*
Genome size (Gb)	4.12
Scaffold N50 (Mb)	256.76
Scaffold number	179
Chromosome number	15
Chromosome percent (%)	98.27
GC content (%)	40.51
Complete ratio of BUSCO (%)	98.6
Illumina reads mapping rate (%)	94.75
Nanopore reads mapping rate (%)	96.33
Repeat sequences (%)	55.68
Number of protein-coding genes	19131
Number of functional annotated genes	12235

Figure 1

Click here to access/download;Figure;Figure 1.jpg

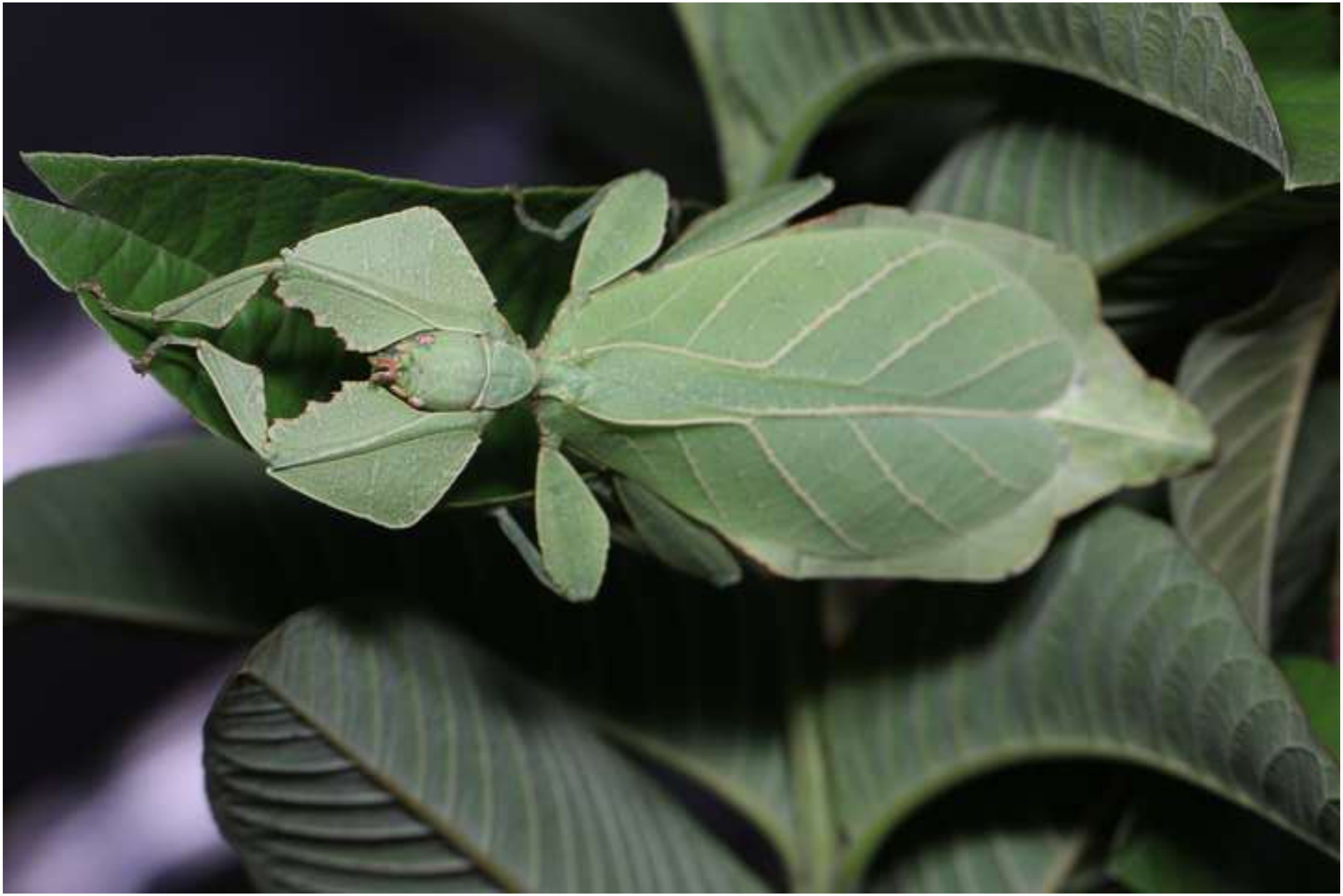

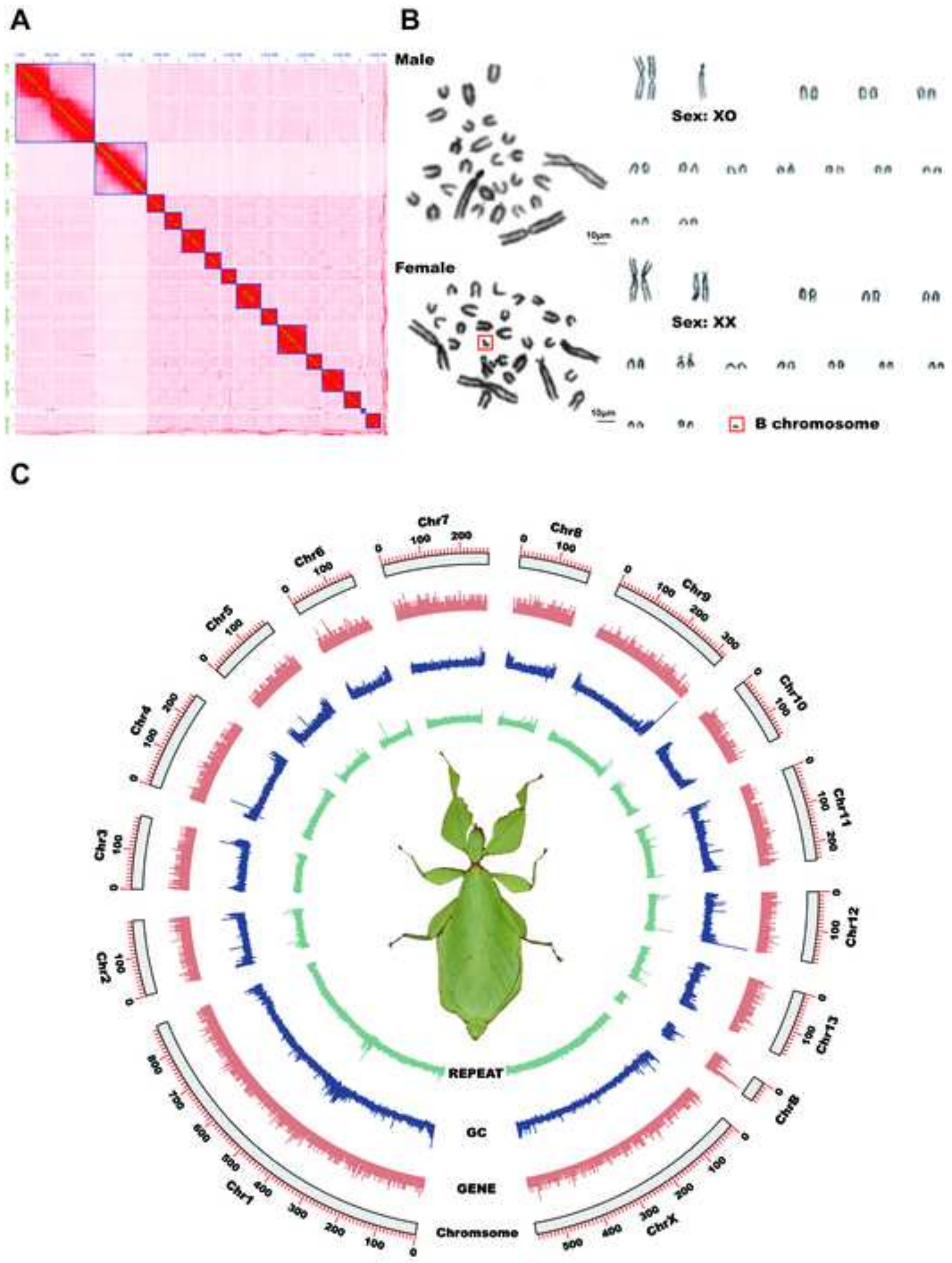
A
B
Male
Sex: XO
10μm
Female
Sex: XX
10μm
B chromosome
C
Chr1
Chr2
Chr3
Chr4
Chr5
Chr6
Chr7
Chr8
Chr9
Chr10
Chr11
Chr12
Chr13
ChrB
ChrX
REPEAT
GC
GENE
Chromsome

Figure 3

Click here to access/download;Figure;Figure 3.tif

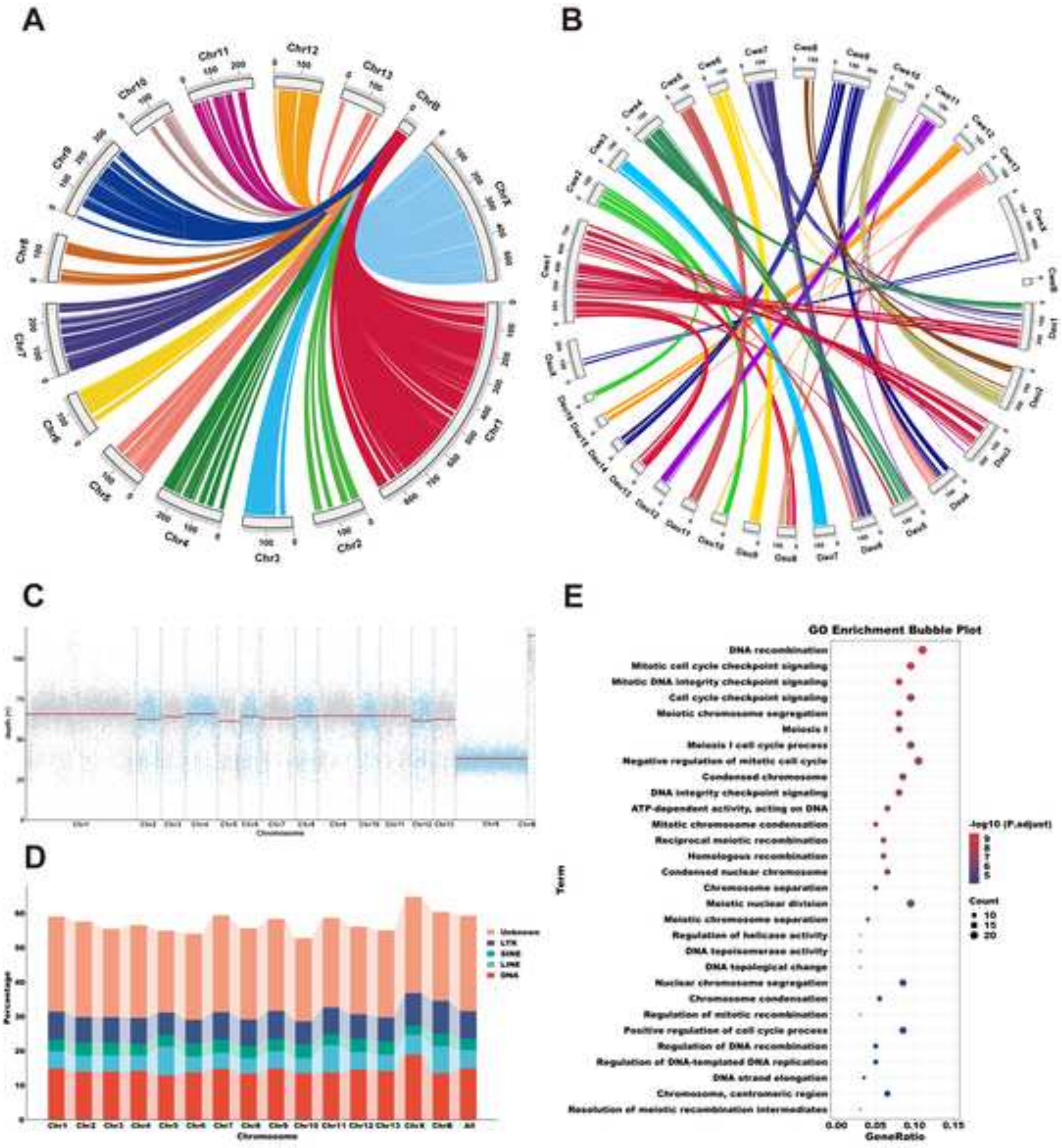

Figure 5

Click here to access/download;Figure;Figure 5.tif

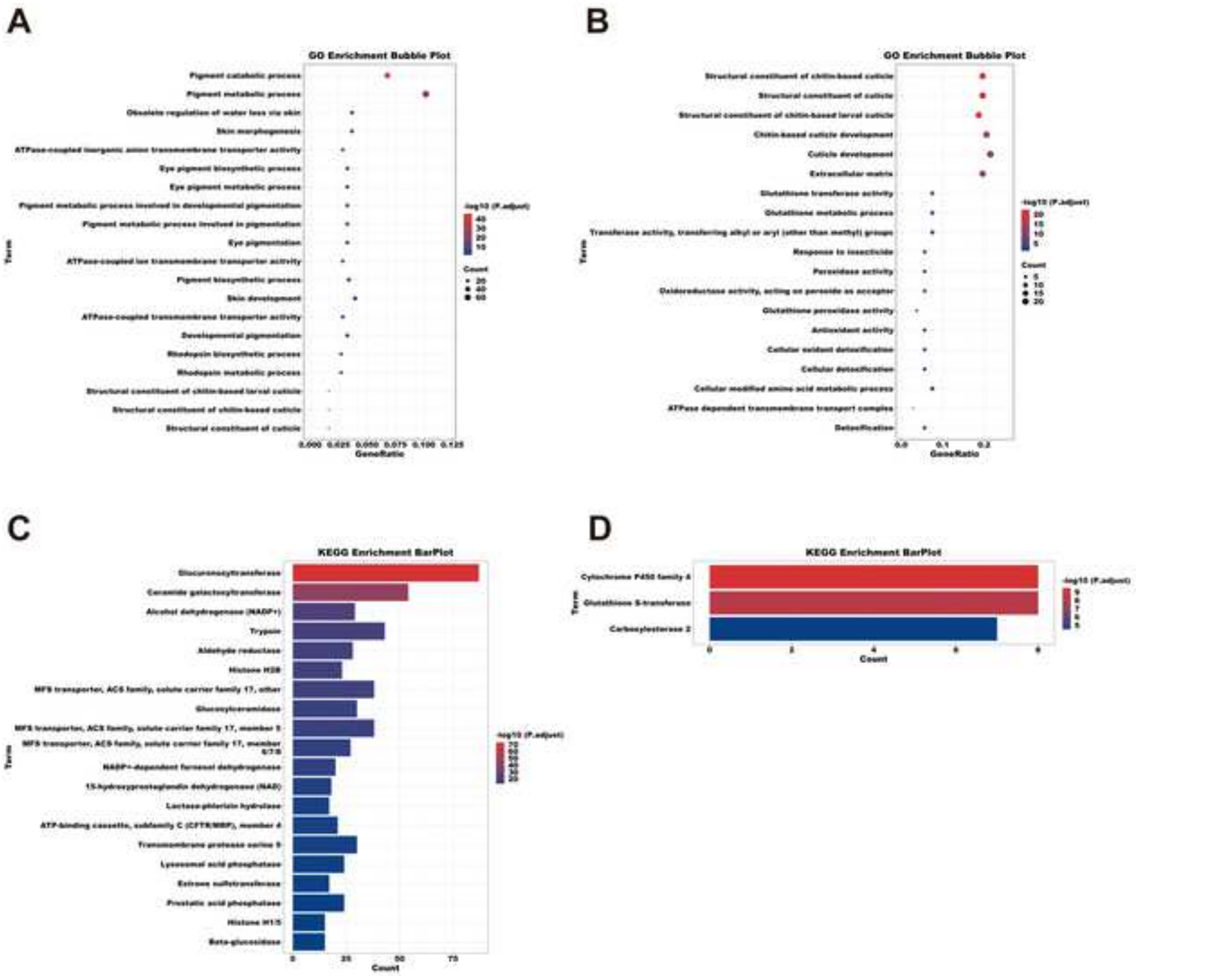

Figure 6 Click here to access/download;Figure;Figure 6.tif

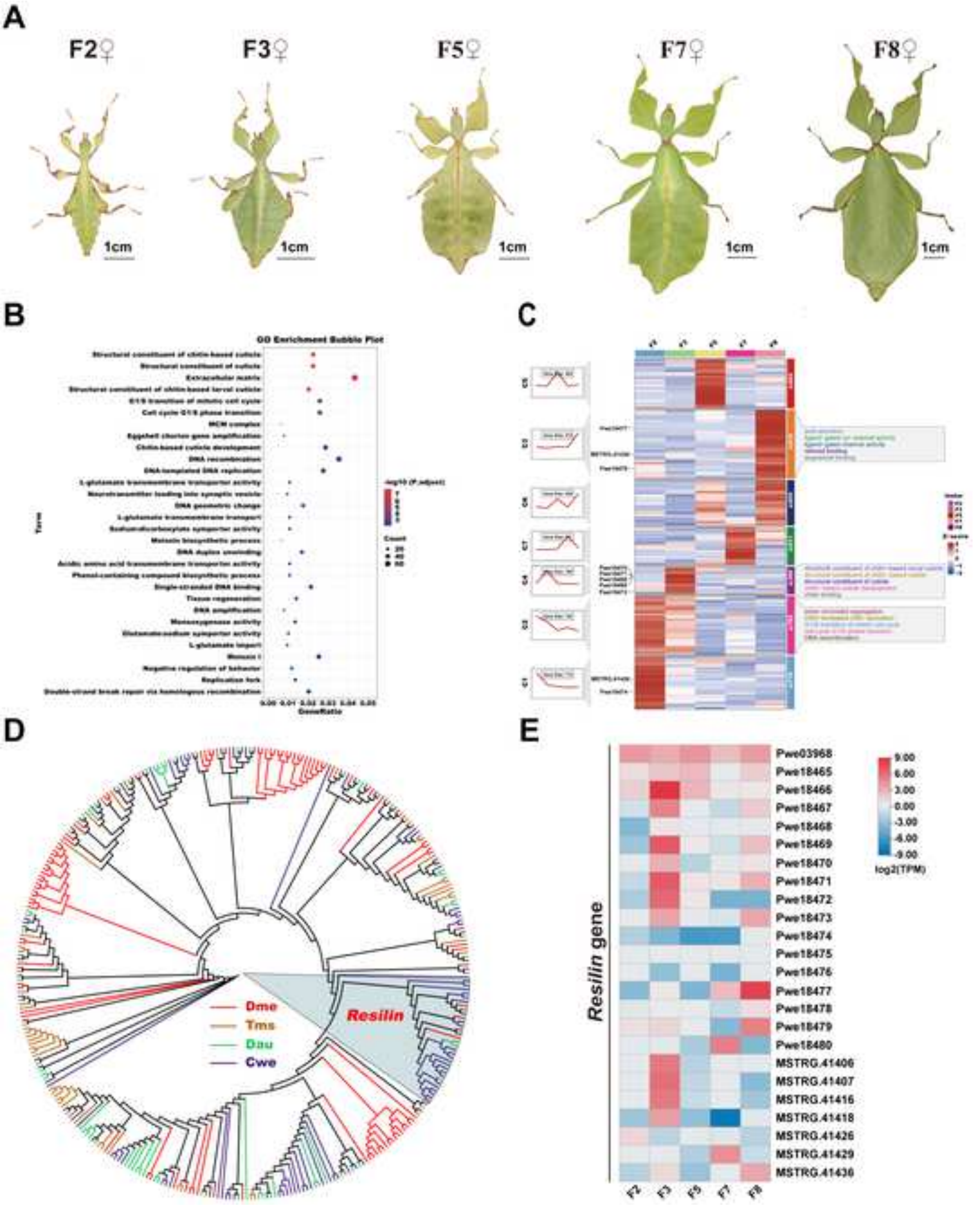

Figure 4

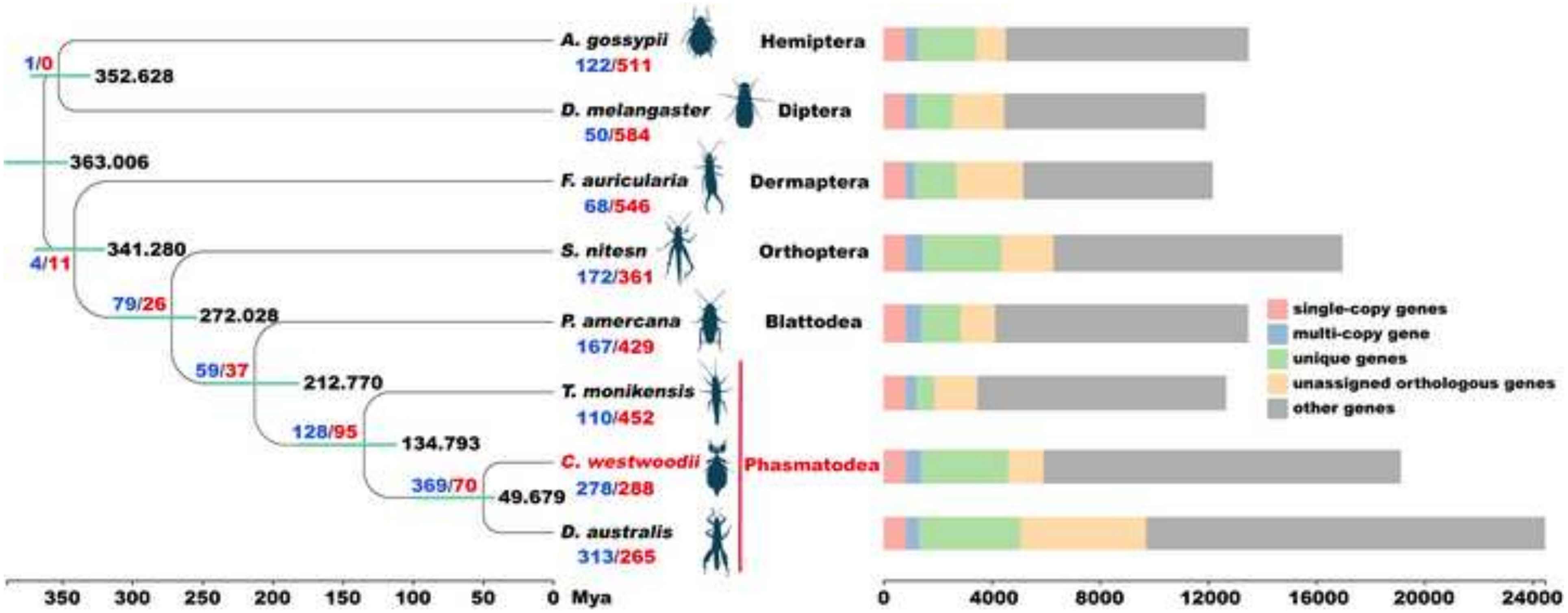

1/0
352.628
363.006
4/11
341.280
79/26
272.028
59/37
212.770
128/95
134.793
369/70
49.679
A. gossypii
122/511
D. melangaster
50/584
F. auricularia
68/546
S. nitesn
172/361
P. amercana
167/429
T. monikensis
110/452
C. westwoodii
278/288
D. australis
313/265
Hemiptera
Diptera
Dermaptera
Orthoptera
Blattodea
Phasmatodea
single-copy genes
multi-copy gene
unique genes
unassigned orthologous genes
other genes
350 300 250 200 150 100 50 0 Mya
0 4000 8000 12000 16000 20000 24000

Click here to access/download
Supplementary Material
Supplementary Figure S1-S5.docx

Supplementary Table S1-S26

Click here to access/download
Supplementary Material
Supplementary Table S1-S26.xlsx

Additional Figure Provided for Review Purposes

Click here to access/download
Supplementary Material
R_Figure_1.tif

Click here to access/download
Supplementary Material
GIGA-D-25-00406-Response letter.docx
