# Supplementary material for: Chromosome-level genome assembly and transcriptomes of the leaf insect Cryptophyllium westwoodii provide insights into the evolution of leaf-like masquerade
Source: Gigascience. 2026 Mar 2;15:giag022. doi: 10.1093/gigascience/giag022 (PMC13108252; doi:10.1093/gigascience/giag022)
Supplement: giag022_Authors_Response_To_Reviewer_Comments_original_submission [file giag022_authors_response_to_reviewer_comments_original_submission.pdf]

Author's Response To Reviewer Comments

Dear Prof. Fan,
We are submitting our revised manuscript entitled “Chromosome-level genome assembly and transcriptomes of the leaf insect Cryptophyllium westwoodii provide insights into the evolution of leaf-like masquerade” (Manuscript ID: GIGA-D-25-00406). We appreciate all the positive comments and valuable suggestions from you and reviewers.

Based on reviewers’ comments and editor’s instructions, we have endeavored to improve our manuscript mainly as follows. (1) We have added a discussion comparing the gene content of the leaf insect B-chromosome to its counterparts in other insects and animals as suggested by reviewer#1. (2) We have extended of the methods used for on the functional enrichment analysis and added a bit more detail about the background gene set and the Benjamini-Hochberg false discovery rate (FDR) correction to the p-value as suggested by reviewer#1. (3) We have tried to incorporate three additional species (Medauroidea extradentata, Clitarchus hookeri, and Bacillus rossius redtenbacheri) into phylogenetic analyses as suggested by reviewer#2. Our result show that the addition of these species don’t affect the topology of tree. To maintain the focus on the core functional genomics objectives, we retained the original in-depth comparative framework centered on Timema monikensis, Dryococelus australis, and C. westwoodii. And we only discussed above species in the Discussion section. (4) We have modified keywords based on the comment of reviewer#2. Additionally, we checked the whole manuscript carefully, corrected typos and improved details of the language and writing. All the changes were highlighted in yellow in the revised version.

We provide detailed point-by-point responses to the reviewers’ comments as follows.

Thank you very much for your consideration.

Xueyan Li, Ph.D
State Key Laboratory of Genetic Evolution & Animal Models, Kunming Institute of Zoology, Chinese Academy of Sciences (CAS), Kunming, Yunnan 650223, China
Email: lixy@mail.kiz.ac.cn, Tel: 86-871-68125339, Fax: 86-871-68125338

Wen Wang, Ph.D
New Cornerstone Investigator
New Cornerstone Science Laboratory
School of Ecology and Environment, Northwestern Polytechnical University, Xi’an 710072, China
Adjunct Professor of State Key Laboratory of Genetic Evolution & Animal Models, KIZ, CAS, Kunming 650223, China
Email: wenwang@nwpu.edu.cn; wwang@mail.kiz.ac.cn

Point-by-Point Responses
Reviewer #1: Dear Dr. Thomas Buckley
The authors have produced a high quality genome assembly. This is a welcome addition to insect genomics. Lead insects are a very interesting taxon and this genome will facilitate a lot of biology.
The finding of the B-chromosome is particularly interesting. This has been known in stick insects previously, but its gene content has not been examined. This is a novel report in that regard.

The analyses are sound, but I have a few questions about functional enrichment and testing.
R: We sincerely thank you for the positive comments on this work. Based on the reviewer’s suggestion, we improved this manuscript as shown in the following.

The finding of the cuticle gene expansion and differential expression is also very interesting. The data at this point suggests a connection between this gene family and the leaf insect form, but this is only a correlation. And there are very few stick insect genomes available. I recommend toning down the statement in the abstract and making it clear this is still a hypothesis.

R: Thank you very much for your suggestion. As suggested, we have revised the statement in the abstract to make it more reasonable: “These findings suggest that cuticle genes, particularly resilin, may contribute to the leaf masquerade morphology in C. westwoodii, implying their possible role in the evolution of this adaptive trait” (Page 2, Lines 13-15).

Finally, I recommend referring to the order as "Phasmatodea" as this is what most researchers use nowadays (e.g., Catalogue of Life). Phasmida is used in older texts, a couple of web sites and a small number of authors.
R: Thank you very much. We have corrected the order name to “Phasmatodea” in the revised manuscript, except for the cited literature

Line 16-18. This sentence needs rewriting for clarity.
R: Thank you very much for the comment. In revised manuscript, we have rewritten this sentence to make it clear: “Very interestingly, among approximately 3500 species of Phasmatodea (representing 21 families), the remarkable adaptation of simulating leaves is almost exclusively limited to the family Phylliidae, which contains just over 100 species, whereas the majority of stick insects mimic branches [9, 12-14]” (Page 3, Lines 16-19).

Line 21. Phyllidae should start with "P" as it is a proper noun.
R: Thank you very much for pointing this. We have corrected it as “Phyllidae” in revised manuscript: “Cryptophyllium westwoodii (Fig. 1) is one of the representative species of the Phylliidae and disguises a nearly impeccable leaf masquerade with a leaf-like venation pattern and lobe-like extensions on the abdomen and legs [15, 16]” (Page 3, Line 22; Page 4, Lines 1-2).

Page 4, line 9. "Combined"
R: Thank you for pointing this. We have corrected it as “Combined” in revised manuscript:. “Combined with karyotyping, we assembled 13 autosomes, one X sex-chromosome and one candidate B-chromosome of this species.” (Page 4, Lines 11-12).

Page 4, line 20. "….which is erected based on eggs…" Not sure of meaning here.
R: Thank you for these comments. In revised manuscript, we have rewritten this sentence to make it clear: “C. westwoodii is from a population reared in Kunming, Yunnan, China with Rubus sp. as host plant in our lab, and this population was established from eggs originally collected from Muang Fuang, Nang Ha, Laos in 2017 [15].” (Page 4, Lines 21-22; Page 5, Line 1).

Page 4, line 21. Individual rather than "larva".
R: Thank you for the comment. We have corrected it as “individual” in revised manuscript: “One individual of the first-instar was collected for sequencing on the Illumina platform for genome survey and correct errors.” (Page5, Lines 1-2).

Page 4, line 22. "…sequencing on the Illumina platform…"
R: Thank you very much. We have corrected it as suggested: “One individual of the first-instar was collected for sequencing on the Illumina platform for genome survey and correct errors.” (Page5, Lines 1-3).

Page 6, line 3. No need to refer to this technology as "next-generation".
R: Thank you very much. We modified it as suggested: “For Illumina sequencing, genomic DNA was isolated from whole body of one larva individual using TreliefTM Animal Genomic DNA Kit (TsingKe, China).” (Page 6, Line 6).

Page 6, line 13. Refer to this as "Oxford Nanopore long read sequencing". This is the underlying technology, where as PromethION is the machine.
R: Thank you for the comment. We modified it as suggested: “For Oxford Nanopore long read sequencing, genomic DNA from another male adult was isolated to construct long DNA fragment libraries (NextOmics, China).” (Page 6, Line 16).

It probably would have been better to use tissue from the same individual as was sequenced with ONT for

the polishing.
R: Thank you for your insightful and constructive comment regarding the use of polishing data. We fully agree with you that the optimal strategy for polishing a de novo genome assembly is to use short-read data from the exact same individual as the long-read (ONT) data. In our study, an individual was initially sequenced using short-read technology to perform genome survey. But unfortunately, the DNA yield from this individual was not enough for subsequent Oxford Nanopore long read sequencing. Thus, we collected another individual from the same batch with a highly similar genetic background for Oxford Nanopore long read sequencing.
To ensure the reliability and accuracy of our final assembly, we performed rigorous, multi-faceted quality assessments. The results (Table 1) indicate the assembly is high-quality (completeness, High contiguity, and consistency) and reliable for subsequent analyses. :
• Completeness: The final assembly shows high BUSCO completeness with 98.27%, indicating comprehensive gene space representation.
• High Contiguity: The assembly has a fragment N50 length of 256.76 Mb, demonstrating excellent continuity and a significant reduction in fragmentation.
• Consistency: The Illumina reads used for polishing mapped back to our final assembly with high mapping rates (94.75%), confirming the internal consistency of the consensus sequence.
In summary, based on the above evaluation of genome quality, even though our short-read and the long-read (ONT) data were not derived from the same individual, the impact on the genome assembly results is minimal. We appreciate the reviewer for understanding this.
Table 1: The statistics of genome assembly and annotation in Cryptophyllium westwoodii genome.

Page 7, line. What is meant exactly by "abdominal tissues". This is a very large part of the body with many tissue types. Be specific about exactly what was sampled. This is important for interpreting the gene expression results and for replication.
R: We sincerely thank the reviewer for raising this important point regarding the specificity of our tissue sampling. We agree that precise anatomical description is crucial for the interpretation of gene expression data. In this study, we specifically dissected and sequenced the highly modified part of abdomen, i.e. the laterally leaf-like abdominal expansions, which are the key morphological innovation underlying the leaf masquerade in this species. We have modified the expression into “the laterally leaf-like abdominal expansions”, instead of “abdominal tissues” in the main text, the legends of pictures and charts in the revised manuscript.

Page 9, line 9. "JUICEBOX" not "JUICERBox".
R: Thank you for pointing this. We have corrected it in revised manuscript: “Finally, contig orientation was corrected and the suspicious fragments were moved into unanchored groups by visual exploration of Hi-C heatmaps manually with JUICEBOX (version 1.11.08) [35].” (Page 9, Lines 10-12).

Page 12, line 22. Give a bit more detail on the functional enrichment analysis. What was the background gene set used for the enrichment test?
R: Thank you for these comments. We would like to clarify that we did perform FDR correction in our original analysis and added a bit more detail about the background gene set and the Benjamini-Hochberg false discovery rate (FDR) correction to the p-value: “GO enrichment analysis was performed using annotated genes on the B chromosome, and the background gene set for this analysis was the complete set of annotated genes in C. westwoodii. Significantly enriched GO terms (FDR-adjusted p-value ≤ 0.05, Benjamini-Hochberg method) were retained for further interpretation.” (Page 13, Lines 2-6).

Page 15, line 1. I think you need a false discovery rate correction here. Also, in this section what was the background gene set for functional enrichment tests?
R: Thank you for these comments. We would like to clarify that we did perform FDR correction in our original analysis and added a bit more detail about the background gene set and the Benjamini-Hochberg false discovery rate (FDR) correction to the p-value: “KEGG and GO enrichment analyses were analyzed by DAVID (RRID:SCR_001881) (version 6.8) [80] and KOBAS (RRID:SCR_006350) (version 3.0) [81] for expansion and contraction of gene families, positive selection genes, and rapidly evolving genes. For these enrichment analyses, the entire annotated genes of C. westwoodii served as the background gene set. Significant enriched GO and KEGG terms (FDR-adjusted p-value ≤ 0.05, Benjamini-Hochberg method) were retained.” (Page15, Lines 11-17).

Page 20, line 8. Diverged from Drycocelus lineage?
R: We thank the reviewer for this question and apologize for the lack of clarity in our original manuscript. We have improved it as "the ancestors of C. westwoodii and D. australis diverged ~49.67 million years ago (Mya) (Fig. 4)." (Page 20, Lines 15-16).

Page 21, lines 10-12. This is a conclusion and needs to be moved to "Discussion" section. Nevertheless, I'm not convinced you can ascribe these gene family expansions to the leaf insect body form as there are so many biological differences between leaf and stick insects. Furthermore, you are only comparing one leaf insect and one stick insect genome. I think you can propose this as a hypothesis that needs testing with more data.
R: Thank you much for these comments. We agree with all points raised and have revised the manuscript accordingly to better reflect the preliminary nature of this finding and to speculate that it is related to the development of the cuticle and epidermis. As suggested, we moved the sentence into "Discussion" section and improved them as the followings: "Interestingly, our comparative genomics demonstrated expanded and contracted gene families were functionally enriched in structural constituent of cuticle and skin development, which may affect the development of the cuticle and epidermis in C. westwoodii." (Page 27, Lines 14-17).

Page 22, line 22. This is a conclusion and needs to move to the Discussion section. Also,
R: We thank the reviewer for these comments. We agree that the original phrasing of line 22 was overly conclusive for the Results section. We have opted to rephrase the sentence to better reflect a summary of our findings at this stage in result Part as the following: "The findings highlight a central role for Cuticle genes in the abdomen tissue of female individuals at the five developmental stages at both genome level and expression level." (Page 23, Lines 5-8). At the same time, we also made a conclusion in the discussion section of original manuscript: "These findings suggest that Cuticle genes may play important roles in the evolution of leaf-like abdominal extension at both genome-level and expression-level." (Page 27, Lines 20-22).
We thank the reviewer for this suggestion (line 15-18). We fully agree with the principle of moving explanatory and detailed explanations to the "Discussion" section. Based on your feedback, we have reorganized lines 15-18 and related paragraphs to ensure the purity of the "Results" section: "Notably, cluster C4 (369 genes) related to structural constituent of chitin-based cuticle and chitin-based cuticle development, exhibited a significant expression peak specifically during the 3rd instar (Fig. 6C; Supplementary Table S24)." (Page 23, Lines 1-3). In the "Discussion" section, we have discussed this part of the content: "Among ten differentially expressed copies of resilin, five copies were divided into cluster 4 (C4) where the 3rd instar can be an important time node for cuticle genes. At this stage from the 2nd instar to the 3rd instar, two copies of resilin were significantly differentially upregulation while the abdominal extensions presented obviously enlargement a lot and leaf-like body initially formed." (Page 28, Lines 13-17).

Pages 25-26. The discussion on the B chromosome is interesting. How does the gene content of the leaf insect B-chromosome differ from other insects (if known) or animals?
R: Thank you very much for your positive feedback and suggestive comments. Based on your comment, we have analyzed the functions of genes on B-chromosome in different animals, which is summarized in an additional table (Supplementary Table S19). The analysis show that that the gene content of the B chromosome in the leaf-stick insect exhibits dual characteristics: on the one hand, it is conserved in core functions compared to other animals, and on the other hand, it carries unique genes with disparate functions. We now have added the related description in Discussion section as the following: "In the B chromosomes of two locusts Abracris flavolineata [100] and Eyprepocnemis plorans [101], genes directly involved in chromosome formation and cell cycle related functions have also been identified. Thess functional genes are not limited to insects; they has also been also found in the mammals red fox and Chinese raccoon dog [102], lizard Anolis carolinensis [103] and fishes including Astyanax mexicanus, Astyanax correntinus and Astatotilapia latifasciata [100, 104, 105]." (Page 25, Lines 15-21) and " In this study, we found some genes that have not been reported on the B chromosome, such as glycoside hydrolase family 28 (the plant cell wall digestion enzymes) [106, 107], Gustatory receptor 26b (Gustatory receptor) [108, 109] and cytochrome P450 6j1 (disintoxication) [89] (Supplementary Table S19). This finding suggests that the majority of genes on B-chromosomes across different species are functionally

enriched for cell cycle and chromosome condensation, but each specie also can harbor a unique subset of genes with disparate functions" (Page 26, Lines 5-11) and added an additional table (Supplementary Table S19) to analyze the functions of genes on B-chromosome.

Reviewer #2:
Here, the authors generated and now present the first genome of a leaf insect (Phasmatodea: Phylliidae), which is indeed a worthwhile endeavor and provides valuable genetic resources for numerous future studies on the development and evolution of these charismatic creatures. While publication of this kind of results needs to be descriptive to a certain point, the authors do their best to provide intriguing information when presenting their results, among them the assumption that the cuticle gene resilin plays a crucial role in evolution of leaf masquerade. The methodical approaches all appear to be state-of-the-art with necessary and nice figures provided.
There are a few shortcomings nevertheless. Besides minor typos etc. (phasmida must be Phasmida; E. messelensis must be E. messelense; keywords must NOT repeat words from the title!), there are also a few major aspects that need to be addressed.
R: We sincerely thank the reviewer for the positive comments and constructive criticisms. We have improved the manuscript based on your invaluable suggestions as shown in the following.
We have corrected "phasmida" into the proper order name "Phasmatodea", "E. messelensis" into "E. messelense" in the whole manuscript: "In addition, the first fossil leaf insect (E. messelense) [119] presented extensions on the abdominal terga and sterna as in modern leaf insects [120]" (Page 27, Lines 7-9).
We have also revised the list of keywords to ensure no repeat words from the title. The new keywords in revised version are "Phylliidae, Cryptophyllium westwoodii, high quality genome, comparative genomics, Cuticle genes, leaf-resembling morphology" (Page 2, Lines 19-20).

Lines 7-9: "The assembled genome size of C. westwoodii is 4.09 Gb, which is the largest one in the published genomes of the order Phasmida …"
This statement is not correct: Clitarchus hookeri, a stick insect from New Zealand, has a larger genome with 4.2 Gb published by Wu et al. (BMC Genomics (2017) 18:884 (DOI 10.1186/s12864-017-4245-x "Assembling large genomes: analysis of the stick insect (Clitarchus hookeri) genome reveals a high repeat content and sexbiased genes associated with reproduction ") a paper, which has obviously been overlooked by the authors and needs to be thoroughly discussed and probably included in the analyses provided here.
R: We thank the reviewer for the valuable comment. We carefully checked the reference Wu et al., 2017 (on Clitarchus hookeri), and cited it in our revised manuscript as reference [92] as shown in the following: "Compared with previously published genomes of walking sticks of the order Phasmatodea [63, 66, 91-94], the chromosome-level assembly of C. westwoodii represents the highest-quality assembly with the second highest N50 value (N50=256.87 Mb) and the lowest number of scaffolds (n=179) (Supplementary Table S4)." (Page 24, Lines 6-10).. It is noted that the initial survey assembly by Wu et al. (2017) estimated a size of 4.2 Gb for Clitarchus hookeri, but a chromosome-level genome assembly for C. hookeri later published by Choi et al (Molecular Ecology (2023):17422. DOI: 10.1111/mec.17422 "Multiple hybridization events and repeated evolution of homoeologue expression bias in parthenogenetic, polyploid New Zealand stick insects", which we had already cited in our original manuscript as reference [91]) has revised the genome size down to approximately 2.8 Gb. This refinement of Clitarchus hookeri genome resulted from improved genome assembly technologies which resolve complex repetitive regions more accurately. Therefore, our conclusion that the C. westwoodii genome (4.09 Gb) is the largest currently published in Phasmatodea is appropriate when referencing the latest and highest-quality assemblies. We have already compiled information on each published genome of walking sticks of the order Phasmatodea in the original manuscript, please refer to Supplementary Table S4.
Supplementary Table S4. The comparison of assembly metrics among Cryptophyllium westwoodii and other five stick insects of the order Phasmatodea. Statistical indicators include genome size, Scaffold N50 length and number, GC cnontent, Repeat content and BUSCO rate.

There is also another genome paper not mentioned/evaluated: Kraus et al. PLoS One. 2009 Sep 29;4(9): e7223. doi: 10.1371/journal.pone.0007223 "The Genome of the Stick Insect Medauroidea extradentata Is Strongly Methylated within Genes and Repetitive DNAs" but I am not sure how relevant this paper might be for the current study.
R: We sincerely thank the reviewer for the comment. We carefully read the paper of Kraus et al. (2009) on

the methylome of Medauroidea extradentata, and appreciate the important contribution of this work to the genomic characterization of this species. Nevertheless, considering our current study focused on the genome assembly and annotation, rather than on DNA methylation patterns, we didn't cite this aricle, but we have cited the more recent genome assembly of M. extradentata by Brand et al. G3: Genes, Genomes, Genetics. 2018;8(5):1403–1408 "The draft genome of the invasive walking stick, Medauroidea extradendata, reveals extensive lineage-specific gene family expansions of cell wall degrading enzymes in phasmatodea" as reference [94] in our main text as follows: "Compared with previously published genomes of walking sticks of the order Phasmatodea [63, 66, 91-94], the chromosome-level assembly of C. westwoodii represents the highest-quality assembly with the second highest N50 value (N50=256.87 Mb) and the lowest number of scaffolds (n=179) (Supplementary Table S4)." in our initial manuscript (Page24, Line 6-10). This paper focus on genome structure and gene family evolution, which is more immediately relevant to the current study. To compare the quality of the genome we assembled, we have already compiled information on each published genome of walking sticks of the order Phasmatodea including M. extradentata in the original manuscript, please refer to Supplementary Table S4.
Supplementary Table S4. The comparison of assembly metrics among Cryptophyllium westwoodii and other five stick insects of the order Phasmatodea. Statistical indicators include genome size, Scaffold N50 length and number, GC cnontent, Repeat content and BUSCO rate.

Beyond those studies, I think that most relevant papers are referenced. Not so the leaf insect paper by Bank and colleagues ("A tree of leaves: Phylogeny and historical biogeography of the leaf insects (Phasmatodea: Phylliidae" Communications Biology 2021, doi.org/10.1038/s42003-021-02436-z) which should be consulted whether it bears further valuable information on these insects.
R: We thank the reviewer for the suggestion. In the revised version of our manuscript, we have now cited this paper in the Introduction section as reference [9]: "Very interestingly, among approximately 3500 species of Phasmatodea (representing 21 families), the remarkable adaptation of simulating leaves is almost exclusively limited to the family Phylliidae, which contains just over 100 species, whereas the majority of stick insects mimic branches [9, 12-14]" (Page 3, Lines 16-19) and "A more fascinating example of masquerade is from leaf and stick insects in the Order Phasmatodea, which mainly distribute in tropical regions and temperate regions [9-10]." (Page 3, Lines 12-13).

I summary, when the studies omitted only need to be discussed in the present manuscript, it can be published after minor revision. If the data from these studies need to be included in the analyses, a major revision is definitely needed. The phylogeny presented is nearly meaningless given the few taxa included and would benefit from inclusion of Clitarchus and Medauroidea for sure.
R: We sincerely thank you for providing all these constructive comments for our manuscipt. We have carefully considered all of your points and have revised the manuscript accordingly. Our point-by-point responses are detailed below. Regarding the omitted studies, we have already compiled information on each published genome of walking sticks of the order Phasmatodea including M. extradentata, C. hookeri and Bacillus rossius redtenbacheri in the original manuscript. These information are used to compare the quality of genome assembly in Discussion section: "Compared with previously published genomes of walking sticks of the order Phasmatodea[63, 66, 91-94], the chromosome-level assembly of C. westwoodii represents the highest-quality assembly with the second highest N50 value (N50=256.87 Mb) and the lowest number of scaffolds (n=179) (Supplementary Table S4). The assembled genome size of C. westwoodii is 4.09 Gb, which is the largest one in the published genomes of the order Phasmatodea (Supplementary Table S4)." (Page 24, Lines 9-15)."
Supplementary Table S4. The comparison of assembly metrics among Cryptophyllium westwoodii and other five stick insects of the order Phasmatodea. Statistical indicators include genome size, Scaffold N50 length and number, GC cnontent, Repeat content and BUSCO rate.

In order to check the phylogeny with inclusion of Clitarchus and Medauroidea, we added three species (M. extradentata, C. hookeri and B. rossius redtenbacheri) to perform phylogenetic analysis again. We reconstructed a phylogenomic tree of 11 species based on 841 single-copy orthologs. The phylogenetic relationships of 11 species were well recovered, with all the nodes being strongly supported (UFB/SH-aLRT = 100/100) (R_Figure_1), and show the same topology as that of Figure 4.
Considering that inclusion of three species didn't effect the topology of tree, and that the primary objective of this study was not to discuss phylogenetic relationships, so we only discussed those omitted species in the Discussion section as mentioned above. We have retained our original, focused analytical framework

for the core genomic comparisons. This approach allows us to maintain a clear and in-depth focus on the functional genomic questions that are the central thrust of our manuscript, without shifting its scope. We hope these revisions thoughtfully address the reviewer's point by acknowledging the limitation, providing the necessary context, and clarifying the intent of our analysis, all while preserving the focused narrative and conclusions of our study.
R_Figure_1. Phylogenetic relationship among the eleven species inferred by the amino acid sequences of the 258 single-copy genes.
Figure 4: Phylogenetic and evolutionary analyses of Cryptophyllium westwoodii genome. In the left panel, blue and red numbers on the branch show the number of expanded and contracted gene families for each clade. The black numbers are divergence times. In the right panel, the numbers of gene families (orthogroups) are shown as barplots. Orthogroups of different categories are in different colors.

Editorial Manager®

Cookie Preference Center

We use cookies which are necessary to make our site work. We may also use additional cookies to analyze, improve and personalize our content and your digital experience. For more information, see our Cookie Policy.

You may choose not to allow some types of cookies. However, blocking some types may impact your experience of our site and the services we are able to offer. See the different category headings below to find out more or change your settings.

Allow all

Manage Consent Preferences

Strictly Necessary Cookies

Always active

These cookies are necessary for the website to function and cannot be switched off in our systems. They are usually only set in response to actions made by you which amount to a request for services, such as setting your privacy preferences, logging in or filling in forms. You can set your browser to block or alert you about these cookies, but some parts of the site will not then work.

Functional Cookies

These cookies enable the website to provide enhanced functionality and personalisation. They may be set by us or by third party providers whose services we have added to our pages. If you do not allow these cookies then some or all of these services may not function properly.

Performance Cookies

These cookies allow us to count visits and traffic sources so we can measure and improve the performance of our site. They help us to know which pages are the most and least popular and see how visitors move around the site.

Targeting Cookies

These cookies may be set through our site by our advertising partners. They may be used by those companies to build a profile of your interests and show you relevant adverts on other sites. If you do not allow these cookies, you will experience less targeted advertising.

Cookie List

Clear

Apply Cancel

Consent Leg.Interest

Confirm my choices

Power
