# Supplementary material for: Chromosome-level genome assembly and transcriptomes of the leaf insect Cryptophyllium westwoodii provide insights into the evolution of leaf-like masquerade
Source: Gigascience. 2026 Mar 2;15:giag022. doi: 10.1093/gigascience/giag022 (PMC13108252; doi:10.1093/gigascience/giag022)
Supplement: giag022_Reviewer_1_Report_original_submission — Reviewer 1 -- 10/23/2025 [file giag022_reviewer_1_report_original_submission.pdf]

Reviewer Report

Title: Chromosome-level genome assembly and transcriptomes of the leaf insect Cryptophyllium westwoodii provide insights into the evolution of leaf-like masquerade.

Version: Original Submission **Date:** 10/23/2025

Reviewer name: Reviewer 1

Reviewer Comments to Author:

The authors have produced a high quality genome assembly. This is a welcome addition to insect genomics. Lead insects are a very interesting taxon and this genome will facilitate a lot of biology.
The finding of the B-chromosome is particularly interesting. This has been known in stick insects previously, but its gene content has not been examined. This is a novel report in that regard.
The analyses are sound, but I have a few questions about functional enrichment and testing.
The finding of the cuticle gene expansion and differential expression is also very interesting. The data at this point suggests a connection between this gene family and the leaf insect form, but this is only a correlation. And there are very few stick insect genomes available. I recommend toning down the statement in the abstract and making it clear this is still a hypothesis
Finally, I recommend referring to the order as "Phasmatodea" as this is what most researchers use nowadays (e.g., Catalogue of Life). Phasmida is used in older texts, a couple of web sites and a small number of authors.
Line 16-18. This sentence needs rewriting for clarity.
Line 21 . Phyllidae should start with "P" as it is a proper noun.
Page 4, line 9. "Combined"
Page 4, line 20. "….which is erected based on eggs…" Not sure of meaning here.
Page 4, line 21. Individual rather than "larva".
Page 4, line 22. "…sequencing on the Illumina platform…"
Page 6, line 3. No need to refer to this technology as "next-generation".
Page 6, line 13. Refer to this as "Oxford Nanopore long read sequencing". This is the underlying technology, where as PromethION is the machine.
It probably would have been better to use tissue from the same individual as was sequenced with ONT for the polishing.
Page 7, line. What is meant exactly by "abdominal tissues". This is a very large part of the body with many tissue types. Be specific about exactly what was sampled. This is important for interpreting the gene expression results and for replication.
Page 9, line 9. "JUICEBOX" not "JUICERBox".
Page 12, line 22. Give a bit more detail on the functional enrichment analysis. What was the background gene set used for the enrichment test?
Page 15, line 1. I think you need a false discovery rate correction here. Also, inn this section what was the background gene set for functional enrichment tests?
Page 20, line 8. Diverged from Drycocelus lineage?

Page 21, lines 10-12. This is a conclusion and needs to be moved to "Discussion" section. Nevertheless, I'm not convinced you can ascribe these gene family expansions to the leaf insect body form as there are so many biological differences between leaf and stick insects. Furthermore, you are only comparing one leaf insect and one stick insect genome. I think you can propose this as a hypothesis that needs testing with more data.
Page 22, line 22. This is a conclusion and needs to move to the Discussion section. Also, line 15-18, move this to Discussion section.
Pages 25-26. The discussion on the B chromosome is interesting. How does the gene content of the leaf insect B-chromosome differ from other insects (if known) or animals?

Methods

Are the methods appropriate to the aims of the study, are they well described, and are necessary controls included? Choose an item.

Conclusions

Are the conclusions adequately supported by the data shown? Choose an item.

Reporting Standards

Does the manuscript adhere to the journal's guidelines on minimum standards of reporting? Choose an item.

Choose an item.

Statistics

Are you able to assess all statistics in the manuscript, including the appropriateness of statistical tests used? Choose an item.

Quality of Written English

Please indicate the quality of language in the manuscript: Choose an item.

Declaration of Competing Interests

Please complete a declaration of competing interests, considering the following questions:

- Have you in the past five years received reimbursements, fees, funding, or salary from an organisation that may in any way gain or lose financially from the publication of this manuscript, either now or in the future?
- Do you hold any stocks or shares in an organisation that may in any way gain or lose financially from the publication of this manuscript, either now or in the future?

- Do you hold or are you currently applying for any patents relating to the content of the manuscript?
- Have you received reimbursements, fees, funding, or salary from an organization that holds or has applied for patents relating to the content of the manuscript?
- Do you have any other financial competing interests?
- Do you have any non-financial competing interests in relation to this paper?

If you can answer no to all of the above, write 'I declare that I have no competing interests' below. If your reply is yes to any, please give details below.

I declare that I have no competing interests

I agree to the open peer review policy of the journal. I understand that my name will be included on my report to the authors and, if the manuscript is accepted for publication, my named report including any attachments I upload will be posted on the website along with the authors' responses. I agree for my report to be made available under an Open Access Creative Commons CC-BY license (http://creativecommons.org/licenses/by/4.0/). I understand that any comments which I do not wish to be included in my named report can be included as confidential comments to the editors, which will not be published.

Choose an item.

To further support our reviewers, we have joined with Publons, where you can gain additional credit to further highlight your hard work (see: https://publons.com/journal/530/gigascience). On publication of this paper, your review will be automatically added to Publons, you can then choose whether or not to claim your Publons credit. I understand this statement.

Yes Choose an item.
