# Supplementary material for: Chromosome-level genome assembly and transcriptomes of the leaf insect Cryptophyllium westwoodii provide insights into the evolution of leaf-like masquerade
Source: Gigascience. 2026 Mar 2;15:giag022. doi: 10.1093/gigascience/giag022 (PMC13108252; doi:10.1093/gigascience/giag022)
Supplement: giag022_Reviewer_2_Report_original_submission — Reviewer 2 -- 11/24/2025 [file giag022_reviewer_2_report_original_submission.pdf]

Reviewer Report

Title: Chromosome-level genome assembly and transcriptomes of the leaf insect Cryptophyllium westwoodii provide insights into the evolution of leaf-like masquerade.

Version: Original Submission **Date:** 11/24/2025

Reviewer name: Reviewer 2

Reviewer Comments to Author:

Here, the authors generated and now present the first genome of a leaf insect (Phasmatodea: Phylliidae), which is indeed a worthwhile endeavor and provides valuable genetic resources for numerous future studies on the development and evolution of these charismatic creatures. While publication of this kind of results needs to be descriptive to a certain point, the authors do their best to provide intriguing information when presenting their results, among them the assumption that the cuticle gene resilin plays a crucial role in evolution of leaf masquerade. The methodical approaches all appear to be state-of-the-art with necessary and nice figures provided. There are a few shortcomings nevertheless. Besides minor typos etc. (phasmida must be Phasmida; E. messelensis must be E. messelense; keywords must NOT repeat words from the title!), there are also a few major aspects that need to be addressed.
Lines 7-9: "The assembled genome size of C. westwoodii is 4.09 Gb, which is the largest one in the published genomes of the order Phasmida …"
This statement is not correct: Clitarchus hookeri, a stick insect from New Zealand, has a larger genome with 4.2 Gb published by Wu et al. (BMC Genomics (2017) 18:884 (DOI 10.1186/s12864-017-4245-x "Assembling large genomes: analysis of the stick insect (Clitarchus hookeri) genome reveals a high repeat content and sexbiased genes associated with reproduction ") a paper, which has obviously been overlooked by the authors and needs to be thoroughly discussed and probably included in the analyses provided here.
There is also another genome paper not mentioned/evaluated: Kraus et al. PLoS One. 2009 Sep 29;4(9):e7223. doi: 10.1371/journal.pone.0007223 "The Genome of the Stick Insect Medauroidea extradentata Is Strongly Methylated within Genes and Repetitive DNAs" but I am not sure how relevant this paper might be for the current study.
Beyond those studies, I think that most relevant papers are referenced. Not so the leaf insect paper by Bank and colleagues ("A tree of leaves: Phylogeny and historical biogeography of the leaf insects (Phasmatodea: Phylliidae" Communications Biology 2021, doi.org/10.1038/s42003-021-02436-z) which should be consulted whether it bears further valuable information on these insects.
I summary, when the studies omitted only need to be discussed in the present manuscript, it can be published after minor revision. If the data from these studies need to included in the analyses, a major revision is definitely needed. The phylogeny presented is nearly meaningless given the few taxa included and would benefit from inclusion of Clitarchus and Medauroidea for sure.

Methods

Are the methods appropriate to the aims of the study, are they well described, and are necessary controls included? Choose an item.

Conclusions

Are the conclusions adequately supported by the data shown? Choose an item.

Reporting Standards

Does the manuscript adhere to the journal's guidelines on minimum standards of reporting? Choose an item.

Choose an item.

Statistics

Are you able to assess all statistics in the manuscript, including the appropriateness of statistical tests used? Choose an item.

Quality of Written English

Please indicate the quality of language in the manuscript: Choose an item.

Declaration of Competing Interests

Please complete a declaration of competing interests, considering the following questions:

- Have you in the past five years received reimbursements, fees, funding, or salary from an organisation that may in any way gain or lose financially from the publication of this manuscript, either now or in the future?
- Do you hold any stocks or shares in an organisation that may in any way gain or lose financially from the publication of this manuscript, either now or in the future?
- Do you hold or are you currently applying for any patents relating to the content of the manuscript?
- Have you received reimbursements, fees, funding, or salary from an organization that holds or has applied for patents relating to the content of the manuscript?
- Do you have any other financial competing interests?
- Do you have any non-financial competing interests in relation to this paper?

If you can answer no to all of the above, write 'I declare that I have no competing interests' below. If your reply is yes to any, please give details below.

I declare that I have no competing interest.

I agree to the open peer review policy of the journal. I understand that my name will be included on my report to the authors and, if the manuscript is accepted for publication, my named report including any attachments I upload will be posted on the website along with the authors' responses. I agree for my report to be made available under an Open Access Creative Commons CC-BY license (http://creativecommons.org/licenses/by/4.0/). I understand that any comments which I do not wish to be included in my named report can be included as confidential comments to the editors, which will not be published.

Choose an item.

To further support our reviewers, we have joined with Publons, where you can gain additional credit to further highlight your hard work (see: https://publons.com/journal/530/gigascience). On publication of this paper, your review will be automatically added to Publons, you can then choose whether or not to claim your Publons credit. I understand this statement.

Yes Choose an item.
